# Catalytic C–H Trifluoromethylation of Arenes and Heteroarenes via Visible Light Photoexcitation of a Co(III)–CF_3_ Complex

**DOI:** 10.1021/acscatal.3c03832

**Published:** 2023-10-09

**Authors:** Christopher S. Kuehner, Andrew G. Hill, Caleb F. Harris, Christian A. Owens, John Bacsa, Jake D. Soper

**Affiliations:** † School of Chemistry and Biochemistry, Georgia Institute of Technology, Atlanta, Georgia 30332-0400, United States; ‡ X-ray Crystallography Center, Department of Chemistry, Emory University, 1515 Dickey Drive, Atlanta, Georgia 30322, United States

**Keywords:** trifluoromethylation, photocatalysis, redox-active ligand, base metal, cobalt

## Abstract

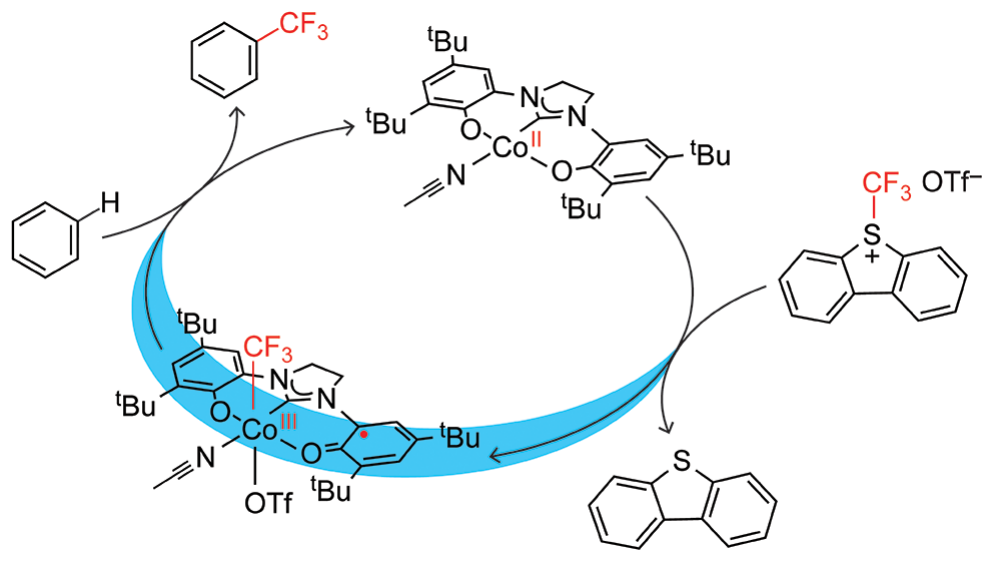

A cobalt photocatalyst for direct trifluoromethylation of (hetero)arene C(sp^2^)–H bonds is described and shown to operate via visible light activation of a Co–CF_3_ intermediate, which functions as a combined chromophore and organometallic reaction center. Chemical oxidations of previously reported (OCO)Co complexes containing a redox-active [OCO] pincer ligand afford a Co–CF_3_ complex two oxidation states above Co(II). Computational and spectroscopic studies are consistent with formulation of the product as [(OCO^•^)Co^III^(CF_3_)(THF)(OTf)] (**II**) containing an open-shell [OCO^•^]^1–^ radical ligand bound to a *S* = 0 Co(III) center. **II** is thermodynamically stable, but exposure to blue (440 nm) light induces Co–CF_3_ bond homolysis and release of ^•^CF_3_, which is trapped by radical acceptors including TEMPO^•^, (hetero)arenes, or the radical [OCO^•^] ligand in **II**. The latter comprises a competitive degradation pathway, which is overcome under catalytic conditions by using excess substrate. Accordingly, generation of **II** from the reaction of [(OCO)Co^II^L] (**III**) (L = THF, MeCN) with Umemoto’s dibenzothiophenium trifluoromethylating reagent (**1**) followed by photolytic Co–CF_3_ bond activation completes a photoredox catalytic cycle for C–H (hetero)arene trifluoromethylation utilizing visible light. Electronic structure and photophysical studies, including time-dependent density functional theory (TDDFT) calculations, suggest that Co–CF_3_ bond homolysis at **II** occurs via an ligand-to-metal charge-transfer (LMCT) (OCO^0^)Co^II^(CF_3_) state, revealing ligand redox activity as a critical design feature and establishing design principles for the use of base metal chromophores for selectivity in photoredox bond activations occurring via free radical intermediates.

## Introduction

The capacity of the trifluoromethyl (CF_3_) group to confer enhanced metabolic stability, bioavailability, lipophilicity, and potency to organic small molecules drives continued efforts to develop new methods for the selective incorporation of C–CF_3_ bonds in pharmaceuticals and agrochemicals.^1−6^ Methods to prepare CF_3_-containing molecules are versatile and robust and include nucleophilic,^7,8^ electrophilic,^9,10^ and radical^11^ CF_3_ transfer processes. A recent emphasis on direct C–H trifluoromethylation has prompted a revisitation of radical alkylations, which can install the CF_3_ functional group in unactivated arenes and heteroarenes.^11,12^ Minisci-type radical functionalization of heteroarenes is not new,^13^ but the past decade has seen a “renaissance” in redox methods for catalytic C–H trifluoromethylation, which include the development of photoredox methods for generation of free ^•^CF_3_.^12,14−19^ The ^•^CF_3_ radical is a strong electrophile, so regioselectivity in these systems, or lack thereof, is determined by the stereoelectronics of the substrate.

Recent advances in selective C–H functionalization by organometallic catalysts suggest a path to selectivity in C–H fluoroalkylations. However, most state-of-the-art methods for C–C coupling cannot reliably be extended to C–CF_3_ bond formation due to the intrinsic properties of the M–CF_3_ intermediates. Whereas early transition metal M–CF_3_ bonds readily undergo α-fluoride abstraction to generate difluoromethyl carbene complexes,^20−22^ M–CF_3_ bonds to low-valent later 3d metals are often thermodynamically robust and kinetically inert.^23^ Accordingly, recent successes in the development of metal-mediated trifluoromethylations activate M–CF_3_ bonds via formation of, for instance, high formal oxidation state complexes, which are prone to C–CF_3_ reductive elimination.^24−26^ In this context, photoredox activation of organometallic catalysts, termed multimetallic or metallaphotoredox catalysis,^19,27^ has received considerable attention for C–C bond formation, including radical alkylations from homolysis of Co(III)–alkyl complexes.^28^ Typically, these processes separate the light-harvesting species from the transition metal catalyst. The role of the chromophore—most commonly a polypyridyl Ru or Ir complex—is to directly activate the metal complex via excited-state oxidation or reduction or to generate a nonmetal free radical coupling partner, which is subsequently trapped at a transition metal center in a catalytic cycle for C–C or C–X coupling.^19^ Although the benefits of these approaches are many, including the capacity to utilize nontraditional reaction partners in cross coupling, bimetallic excited-state reactivity limits the use of base metals in photoredox catalysis.^29−33^ Moreover, separating the chromophore from the bond-forming reaction adds a layer of complexity when metal-mediated selectivity is pursued and opens paths to side reactions from transient free radicals.

Photoinduced M–L bond activations are a staple of organometallic synthesis, and the capacity of cobaloxime organocobalt(III) complexes to generate alkyl radicals via facile Co–C homolysis has been known and exploited for synthesis applications for decades.^28^ Such “visible light induced homolysis” (VLIH) has been proposed as a generalizable alternative to utilize 3d transition metals in photocatalytic applications.^34^

**Figure 1:**
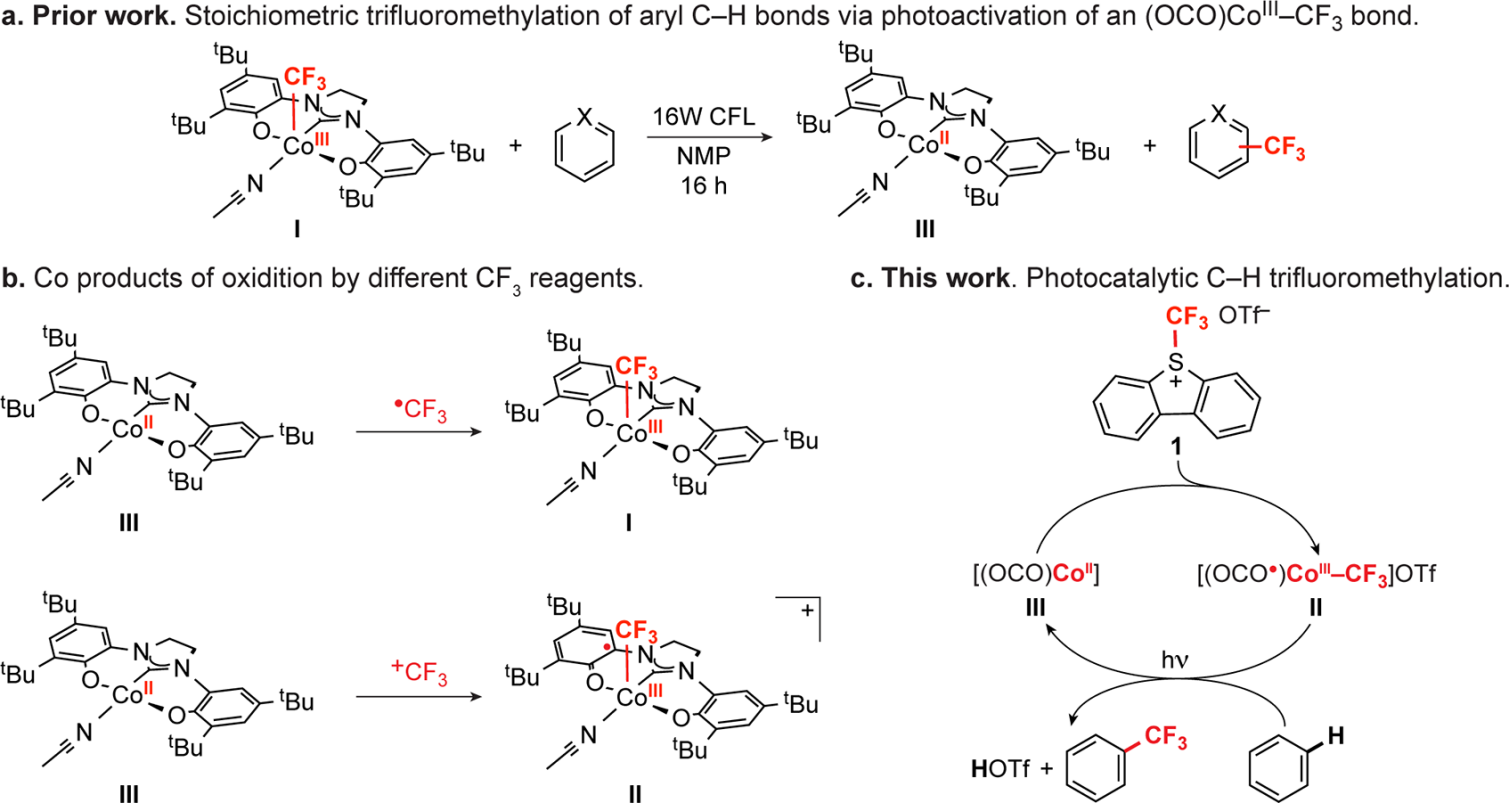
(a) Stoichiometric trifluoromethylation of heteroarene C–H bonds by photoinduced Co^III^–CF_3_ bond activation.^35^ (b) Comparison of oxidation products of Co(II) oxidation produced by formal addition of ^•^CF_3_ vs ^+^CF_3_. (c) Proposed (OCO)Co^II^/(OCO^•^)Co^III^(CF_3_) catalysis cycle for trifluoromethylation of benzene utilizing a ^+^CF_3_ reagent and visible light activation of **II**.

We reported a [(OCO)Co^III^(CF_3_)(MeCN)] (**I**) compound supported by a pincer-type [OCO] ligand that trifluoromethylates unactivated (hetero)arenes upon irradiation by a broad-spectrum compact fluorescent light (CFL) (Figure 1a).^35^ The redox-active [OCO] is an essential feature of the observed reactivity.^36^ It provides access to a low energy ligand-to-metal charge-transfer (LMCT) [(OCO^•^)Co^II^(CF_3_)(MeCN)] redox isomer, which populates a Co–CF_3_ σ* MO, reducing the Co–C bond order to 0.5 and facilitating the release of a persistent ^•^CF_3_ radical, which attacks electron rich arenes. The [(OCO)Co^II^(MeCN)] byproduct subsequently oxidizes the resulting cyclohexadienyl intermediate to afford the products of arene C–H trifluoromethylation without a sacrificial or substrate-derived oxidant. Extensions to catalysis were challenged by deactivation of the (OCO)Co^III^(CF_3_) core upon binding a sixth ligand, precluding the use of donor solvents, and competitive photodegradation of the silver salts used to install the CF_3_ functionality.

Our search for alternative sources of CF_3_ led to electrophilic CF_3_ reagents, such as Umemoto’s *S*-(trifluoromethyl)dibenzothiophenium triflate (**1**), which has been extensively studied for trifluoromethylation.^9,37−40^ Their shelf stability and ease of handling have made them oftentimes preferred over cheaper, alternative CF_3_ sources such as ICF_3_ and HCF_3_,^41^ and over the past decade, these reagents have seen extensive use in catalytic trifluoromethylations. In reactions with these reagents, it is frequently suggested that the transition metal acts as an outer-sphere, one-electron reductant, thus generating ^•^CF_3_ without M–CF_3_ intermediates.^4,42,43^ Addition of a ^+^CF_3_ equivalent to a transition metal is a formal 2e^–^ oxidation of the metal center, which demands a 2e^–^ redox capacity at the metal center. This is exemplified by work of Sanford and co-workers, wherein formal ^+^CF_3_ transfer generates a Ni^IV^–CF_3_ complex, which is active for trifluoromethylation of unactivated arenes.^44^

By analogy, addition of ^+^CF_3_ to our (OCO)Co^II^ complex affords an (OCO)Co(CF_3_) species that is two redox levels above Co(II), and one above the (OCO)Co^III^(CF_3_) complex that was previously demonstrated to be photoactive for trifluoromethylation (Figure 1b). Given the propensity of the redox-active [OCO] ligand to support Co in four formal oxidation states,^36^ we speculated that net ^+^CF_3_ addition to an (OCO)Co^II^ species would afford an (OCO^•^)Co^III^(CF_3_) product and open avenues for catalysis based on a 2e^–^ redox cycle (Figure 1c), but it was unclear whether (OCO^•^)Co^III^(CF_3_) would retain the features necessary for photoactivation of the Co–CF_3_ bond.

Reported herein is a Co catalyzed photoredox method for efficient trifluoromethylation of unactivated arene and heteroarene C–H bonds using **1** and visible light. Mechanistic studies and stoichiometric reactions provide strong evidence of a 2e^–^ redox cycle involving net ^+^CF_3_ addition to a Co(II) complex to generate a photoactive (OCO^•^)Co^III^(CF_3_) species. Combining the chromophore and organometallic reaction center in (OCO^•^)Co^III^(CF_3_) establishes key design principles for selectivity in photoredox radical C–H (fluoro)alkylations.

## Results

### Preparation and Electronic Structure of [(OCO)Co(CF_3_)(THF)OTf] (**II**)

**Figure 2:**
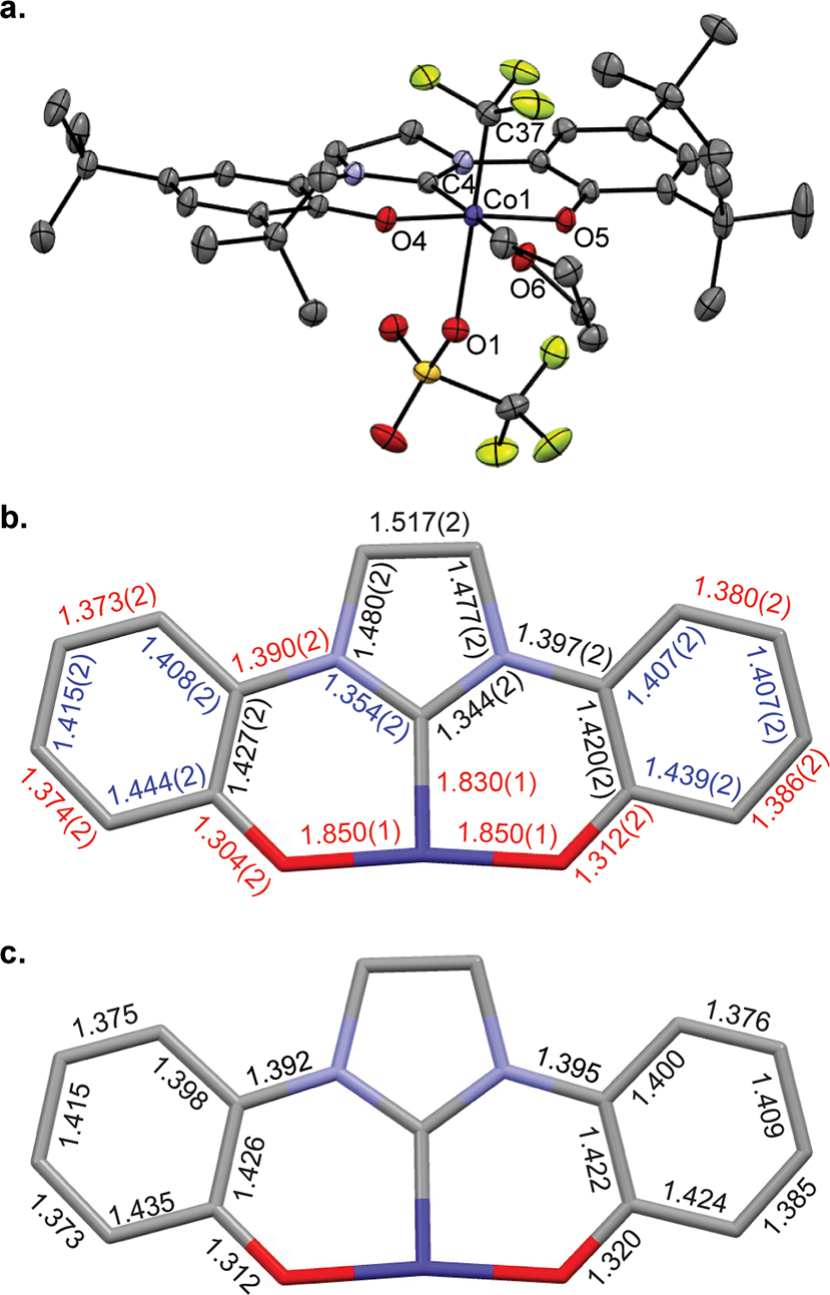
(a) ORTEP plot of [(OCO^•^)Co^III^(THF)(CF_3_)OTf] (**II**). Ellipsoids are drawn at 50% probability. Hydrogen atoms and noncoordinated solvent molecules are omitted for clarity. (b) Schematic of selected bond lengths (Å) for **II**. Bond length changes greater than 0.01 Å versus [(OCO)Co^III^(CF_3_)(MeCN)_2_] (**I**) are indicated by colored labels: red indicates bond contraction; blue indicates bond elongation. (c) Arithmetic mean of the fully reduced [OCO]^2–^ ligand and the doubly oxidized charge neutral [OCO]^0^ form in [(OCO)Co^II^(THF)_3_]^2+^.^36^ Metric data for the isolated [OCO]^2–^ and [OCO]^0^ ligands are reproduced in Figure S30.

Treating a burgundy CH_2_Cl_2_ solution of [(OCO)Co^III^(CF_3_)(MeCN)_*n*_] (**I**) (*n* = 1, 2; [OCO] = 1,3-bis(3,5-di-*tert*-butyl-2-hydroxyphenyl)imidazoline) with 1 equiv of AgOTf gave an immediate color change to olive-green, from which **II** was isolated in 80% yield (eq 1). A sample of the THF adduct of **II** suitable for analysis by X-ray diffraction was prepared by slow evaporation of a concentrated THF:HMDSO (1:1) solution at 25 °C. The structure of **II** is shown in Figure 2a. The Co center is six-coordinate, with the [OCO] pincer ligand occupying three *meridional* sites. An equatorial THF ligand is *trans* to the pincer carbene, and *trans* disposed CF_3_ and OTf ligands complete the quasi-octahedral coordination sphere. Oxidation of **I** to **II** occurs with minor contractions (ca. 0.02 Å) of the Co–O bonds to the phenoxides and the Co–C bond to the carbene (from 1.8428(13) Å in **I** to 1.8300(10) Å in **II**). The Co–CF_3_ bond length is statistically indistinguishable from **I** (1.914(2) Å in **II** vs 1.918(1) Å in **I**). The [OCO] ligand bond metrics for **II** are collected in Figure 2b. As compared to **I**, the ligand metric data show a pronounced quinoid-type pattern of four elongated and two contracted C–C bonds in both phenoxides along with contracted C–O and C–N bonds. These changes are consistent with those expected upon ligand oxidation. Moreover, the [OCO] ligand bond lengths in **II** are nearly identical with the arithmetic mean of those in isolated complexes containing the ligand in its fully reduced [OCO]^2–^ and doubly oxidized charge neutral [OCO]^0^ oxidation states (Figure 2c).^36^ In total, the X-ray data are entirely consistent with formulation of the [OCO] ligand in **II** as a monoanionic [OCO^•^]^−^ free radical with the charge distributed symmetrically across the ligand framework. Accordingly, the sum of the solid-state data suggests that **II** is best formulated as [(OCO^•^)Co^III^(CF_3_)(THF)OTf].
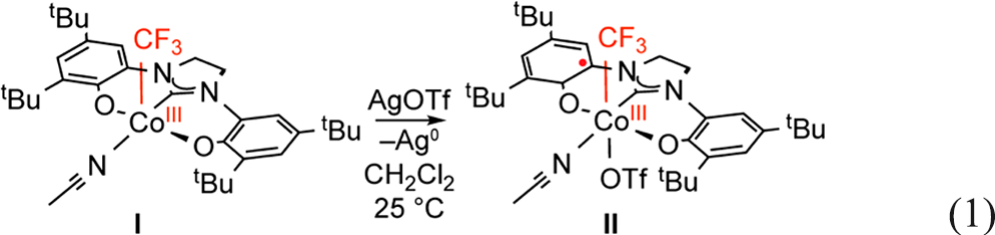
1

Consistent with this assignment, the solution magnetic moment (μ_eff_) of **II** of 1.92 in THF-*d*_8_ is slightly higher than the spin-only value for an *S* = 1/2 complex. Plausible electronic structures include a low-spin Co(III) center with a single unpaired electron on the [OCO^•^]^−^ ligand or an intermediate spin *S* = 1 Co(III) center antiferromagnetically coupled to an [OCO^•^]^−^ ligand radical. An alternative Co(II) formation with a doubly oxidized [OCO]^0^ ligand is inconsistent with the X-ray metric data and the thermal stability of the Co–CF_3_ bond (*vide infra*).

**Figure 3:**
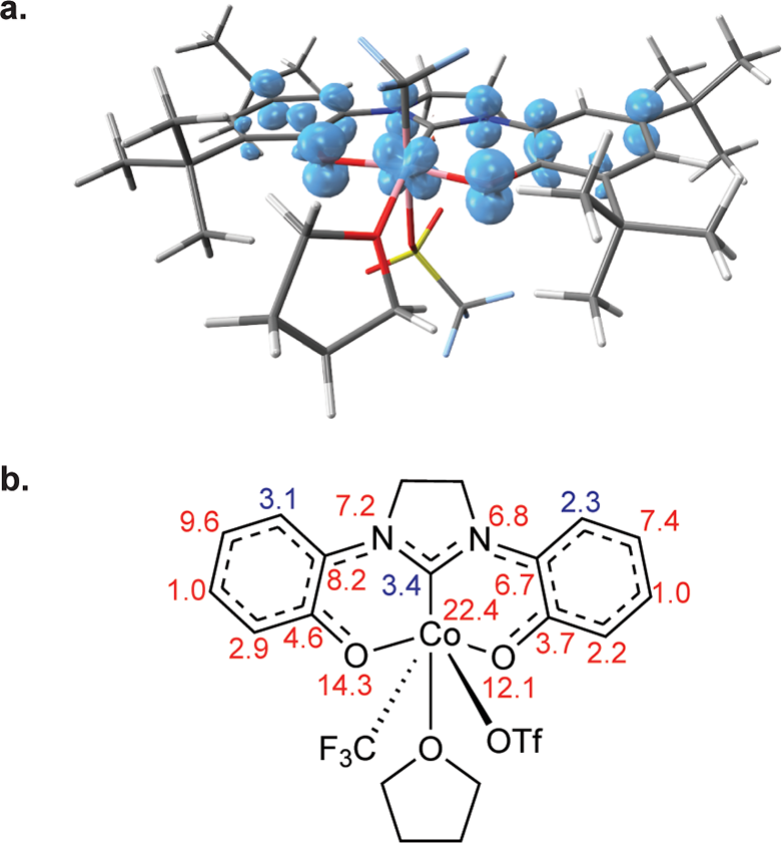
(a) Spin density plot of **II** (*S* = 1/2) generated with IQMol (isosurface value 0.004). (b) Spin density per atom for **II**. Spin down density is shown in blue.

To distinguish these possibilities, the electronic structure of **II** was computed with unrestricted DFT calculations (BP86, def2-TZVP) starting in the experimentally determined doublet state. The bond lengths of the optimized geometry in the doublet state were compared to those of the single-crystal X-ray structure and found to have a mean absolute error of 0.013 Å. In the (OCO)Co^III^(CF_3_) fragment, a maximum bond length deviation of 0.006 Å was observed, suggesting that the spin state, functional, and basis set (BP86, def2-TZVP) used for geometry optimization accurately capture the bond distances in the complex. The spin density per atom in the optimized geometry was also calculated. Complex **II** converged as a doublet (⟨*s*^2^⟩ = 0.7533) with 22.4% of the total spin density being located at cobalt (Figure 3). The balance of the spin density is delocalized over both phenoxide arms of the OCO ligand, mainly on the oxygen atoms (total of 26.4%). A small amount of spin-down density on the NHC and two aryl carbons can be attributed to spin polarization. A quartet state (⟨*s*^2^⟩ = 3.7996) is computed to be +33 kcal mol^–1^ higher in energy than the doublet state, making its involvement highly unlikely. UCO analysis of α and β orbitals showed only one orbital with an overlap integral less than 0.999: the singly occupied molecular orbital distributed across the Co center and the [OCO] ligand.^45,46^ The computed structure of **II** is therefore most consistent with an [(OCO^•^)Co^III^(CF_3_)(THF)OTf] assignment with a low-spin Co(III) center and a monooxidized [OCO^•^]^−^ ligand radical, corroborating experimental observations and computation of very similar systems.^35,36^ Partial delocalization of the unpaired spin onto the Co center reflects significant covalency in the metal–ligand bonding or minor contributors to the ground state, which would not be readily evident in common spectroscopic methods.

### Absorption Spectrum and Photochemistry of [(OCO^•^)Co^III^(CF_3_)(THF)OTf] (**II**)

**Figure 4:**
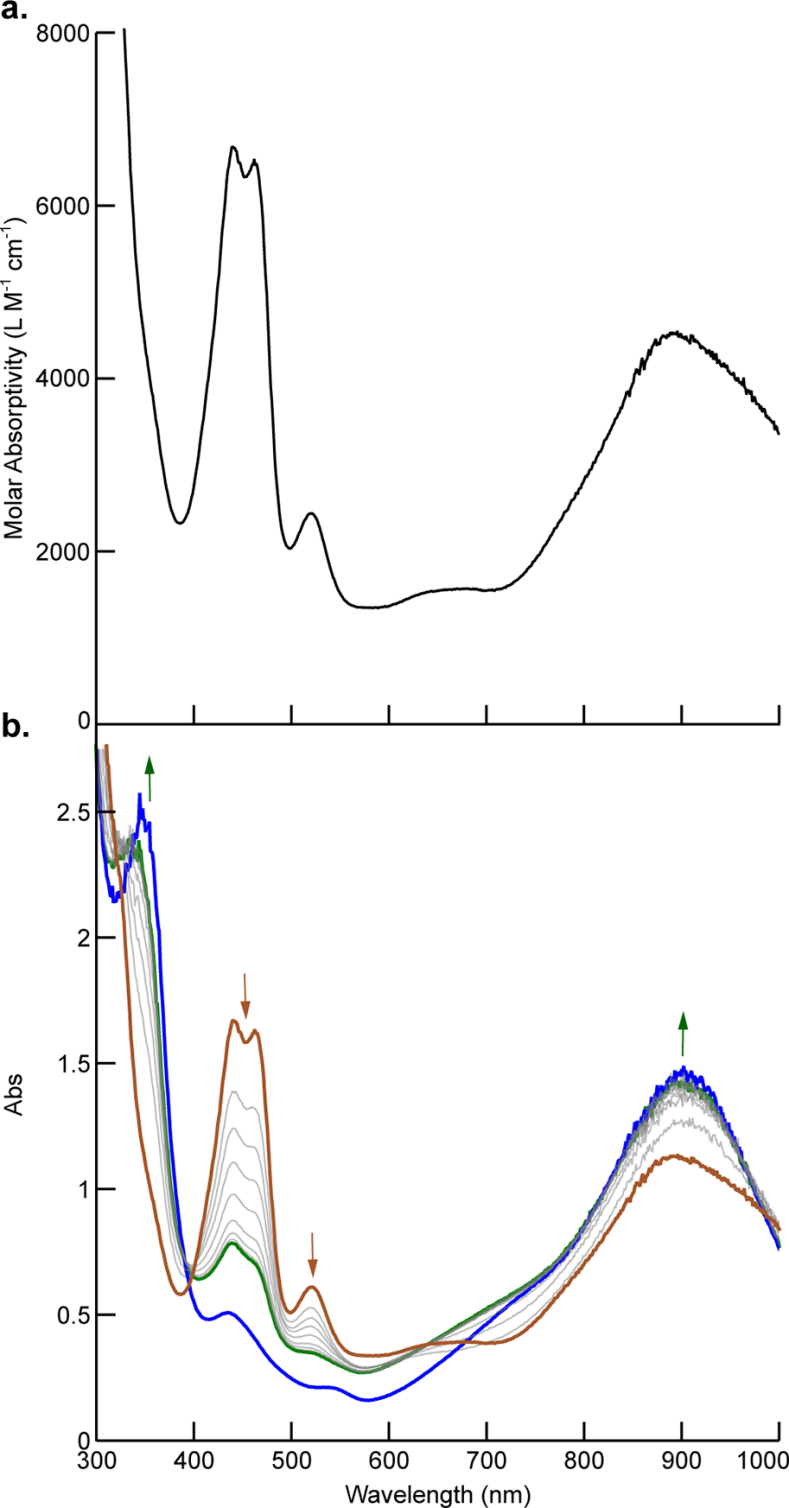
UV–vis absorption spectra of (a) 0.25 mmol of **II** in CH_2_Cl_2_ at 25 °C and (b) upon exposure to a Kessil KSPR 160L-440 LED lamp. Spectra are shown at *t* = 0 (brown line) and 0.5 h intervals to *t* = 4 h (green line). A spectrum of isolated [(OCO)Co^III^(THF)_2_]OTf (**IV**) in CH_2_Cl_2_ (blue line) is shown for comparison.

**Figure 5:**
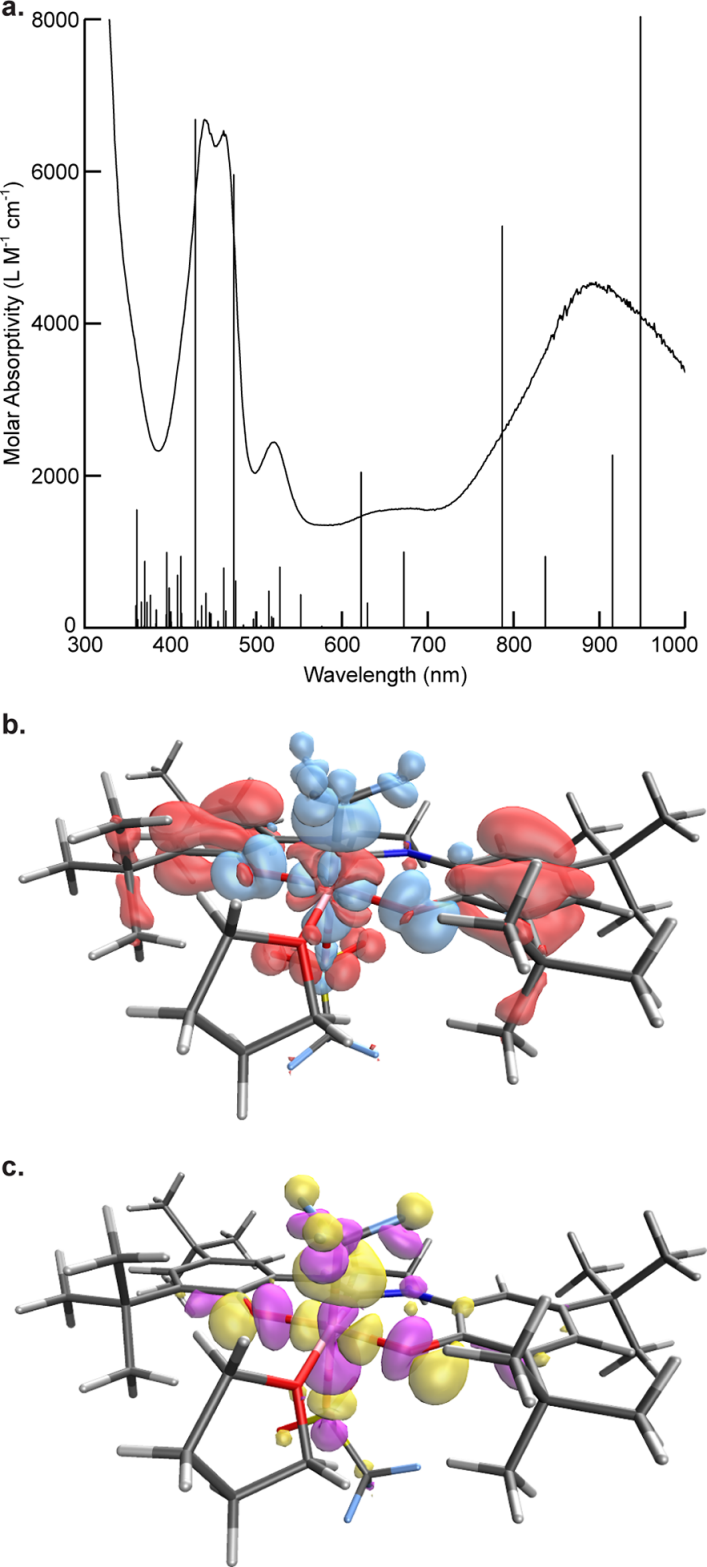
(a) Overlay of simulated and experimental UV–vis absorption spectra of **II**. (b) Difference density CEJ plot of the calculated transition at 447 nm plotted in IQMol, isovalue 0.001. Positive density is shown in blue, and negative density is shown in red. (c) Calculated LUMO plotted in IQMol, isovalue 0.030.

The UV–vis spectrum of **II** in CH_2_Cl_2_ (Figure 4a) exhibits four CT bands at 438, 460, 518, and 889 nm (ε = 2400–6600 M^–1^ cm^–1^). Excited states were examined by time-dependent density functional theory (TDDFT) calculations (BP86, def2-TZVP) from the doublet ground state. The calculated and experimental UV–vis spectra of complex **II** are in strong agreement (Figure 5a). The difference density CEJ plot of the transition at 447 nm (Figure 5b) shows strong ligand-to-metal charge-transfer (LMCT) character and results in 89% population of the calculated LUMO (Figure 5b), which has significant antibonding character between the Co center and the CF_3_ ligand, suggesting photoexcitation of **II** with 447 nm light should significantly destabilize the Co–CF_3_ bond toward homolysis.

Consistent with this hypothesis, photolysis of a 0.25 mmol solution of **II** in CH_2_Cl_2_ using a Kessil KSPR 160L-440 LED lamp resulted in a measurable decrease in the intensity of the CT bands at 438, 460, and 518 nm within minutes, with a concomitant increase in intensity and a red shifting of the band at 890 to 902 nm and appearance of a new band at 337 nm (Figure 4b). Isosbestic points at 400 and 625 nm are consistent with clean conversion to a single product or mixture of products without formation of observable intermediates. The spectrum after 4 h of continuous photolysis closely matched that of isolated [(OCO)Co^III^(THF)_2_]OTf (**IV**).^36^ Photoinduced homolysis of the Co^II^–CF_3_ bond occurs at an LMCT excited state of **II** to afford an excited-state intravalence isomer of **IV**. The net conversion of **II** to **IV** is balanced by the loss of ^•^CF_3_. The fate of the ^•^CF_3_ under these conditions was not determined, but CF_3_Cl is most likely from reaction with the CH_2_Cl_2_ solvent. The UV–vis spectrum of **II** in CH_2_Cl_2_ was unchanged over 7 h in the dark at 50 °C, suggesting that Co–CF_3_ homolysis occurs from a photoexcited state and not thermolysis from heating by the light source (Figure S2).

### Photoinduced ^•^CF_3_ Transfer from [(OCO^•^)Co^III^(CF_3_)(THF)OTf] (**II**)

**Scheme 1:**
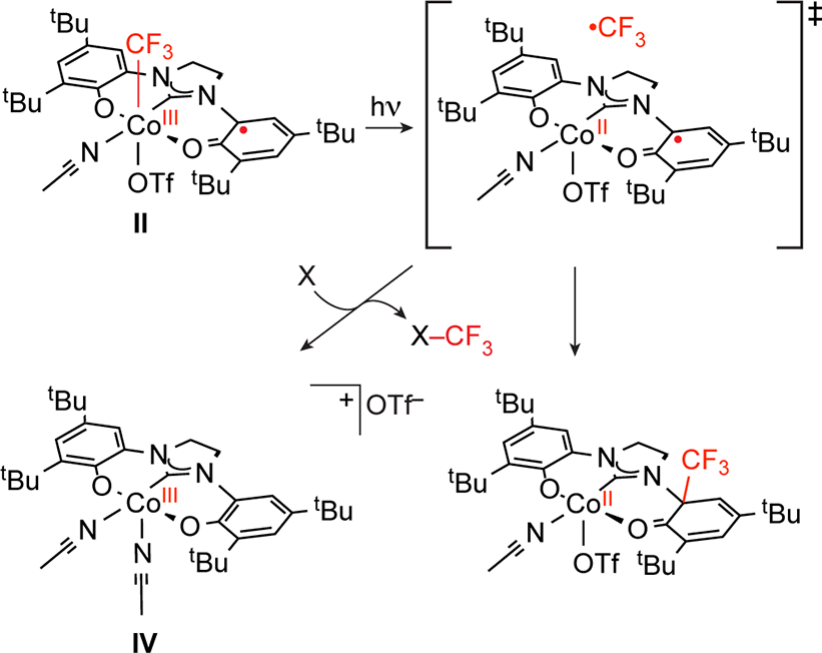
Reaction Pathways for Trifluoromethylation Following Photolysis of **II** X = TEMPO^•^ or (hetero)arene.

Exposure of a 7.5 mM CH_2_Cl_2_ solution of **II** containing 10 equiv of 2,2,6,6-tetramethylpiperdine-1-oxyl (TEMPO^•^) to a 16 W compact fluorescent lamp (CFL) afforded TEMPO–CF_3_ in 12% yield after 16 h, as determined by ^19^F NMR spectroscopy, along with unidentified CF_3_-containing byproducts. Analysis of the reaction mixture by GC–MS revealed a second product with a molecular weight of 579 *m*/*z*, consistent with CF_3_ addition to the [OCO] ligand and demetalation. Whereas increasing the concentration of TEMPO^•^ to 15 equiv at the same concentration of **II** resulted in an increase in yield of TEMPO–CF_3_ to 24%, increasing both the TEMPO^•^ and **II** concentration by 60% resulted in a 40% decrease in TEMPO–CF_3_ yield (7%). These observations are consistent with a bifurcated reaction wherein generation of free ^•^CF_3_ gives access to two competing reactions (Scheme 1). Accordingly, increasing the TEMPO^•^ concentration relative to [**II**] increases the probability of ^•^CF_3_ trapping by TEMPO^•^ and TEMPO–CF_3_ formation; increased [Co] increases the probability of unproductive trapping at the ligand backbone and deactivation of the complex.

**Figure 6:**
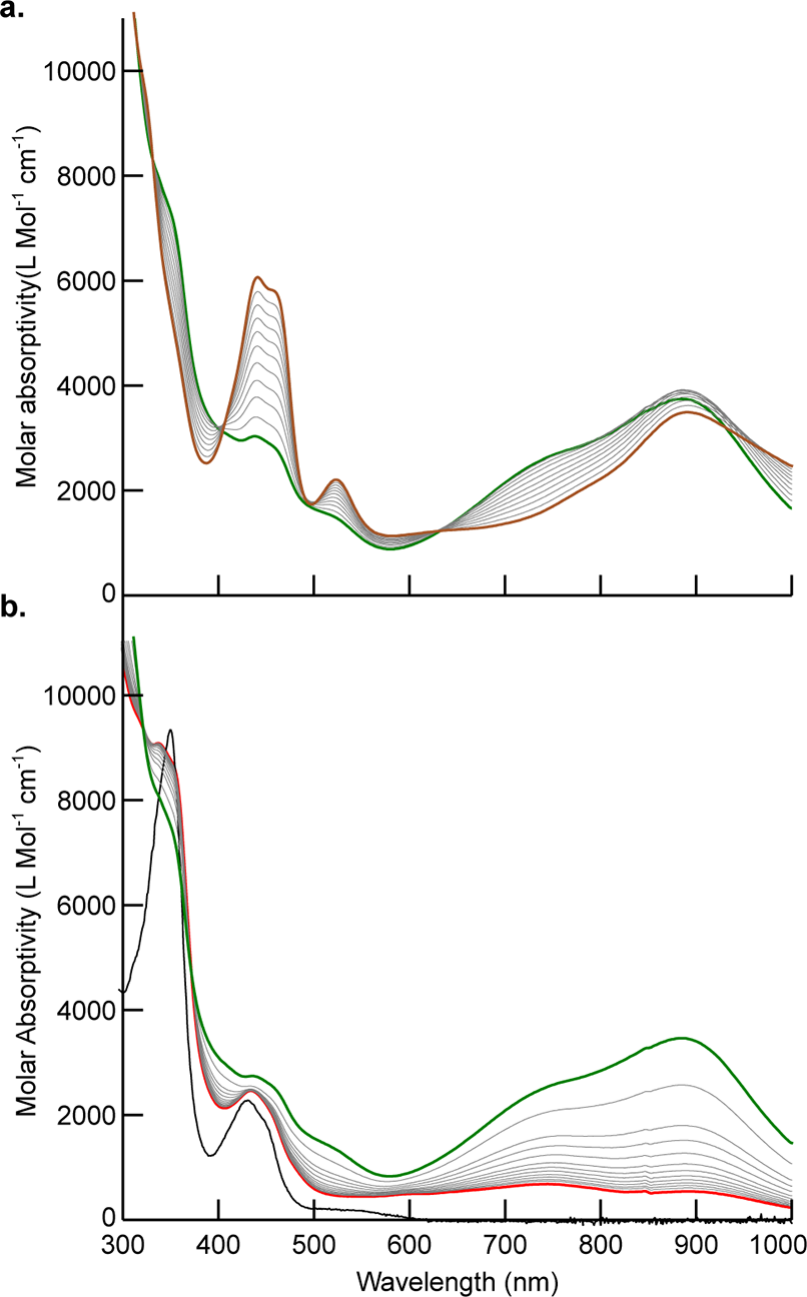
Absorption spectra of a 0.23 mmol solution of **II** in C_6_H_6_ with exposure to a Kessil KSPR 160L-440 LED lamp. (a) Spectra acquired at 5 min intervals from *t* = 0 (brown) to *t* = 55 min (green). (b) Spectra acquired at 5 min intervals from *t* = 55 m intervals (green) to *t* = 120 m (red). A spectrum of isolated [(OCO)Co^II^(THF)] (**III**) in C_6_H_6_ (black) is shown for comparison.

Photolysis of a 0.025 mM CH_2_Cl_2_ solution of **II** using a Kessil KSPR 160L-440 LED lamp in the presence of 5 equiv of C_6_H_6_ afforded α,α,α-trifluorotoluene in 10% yield after 6 h, as determined by ^19^F NMR spectroscopy. Consistent with the partitioning experiments described above, performing the reaction in neat C_6_H_6_ increased the yield of α,α,α-trifluorotoluene to 58% in 6 h. Monitoring the reaction by UV–vis spectroscopy shows that the reaction occurs in two sequential phases. Consumption of **II** initially generates a spectrum that closely resembles **IV** with two quasi-isosbestic points at 637 and 405 nm (Figure 6a).^36^ Continued photolysis results in bleaching of the intermediate peaks at 889, 732, and 443 nm with concomitant growth of a feature at 357 nm, which is diagnostic of [(OCO)Co^II^(THF)] (**III**) (Figure 6b). The net conversion of **II** + C_6_H_6_ to **III** + α,α,α-trifluorotoluene is balanced by loss of 1 equiv of triflic acid (HOTf) (eq 2). HOTf formation, in the form of a triflate salt, is evident in catalytic reactions as a singlet at −79 ppm in the ^19^F NMR spectrum of reactions performed in J. Young NMR tubes (*vide infra*).^47^ Its appearance in stoichiometric reactions, however, is frequently obscured, presumably by an interaction with paramagnetic **III** or a rapid exchange process. Accordingly, addition of 1.0 equiv of Hünig’s *N*,*N*-diisopropylethylamine (^i^Pr_2_EtN) base to reaction mixtures of **II** + C_6_H_6_ resolves the HOTf byproduct as [^i^Pr_2_EtNH]OTf, which is manifested in the ^19^F NMR spectrum as a sharp singlet at −79 ppm upon photolysis (Figures S9–S10).
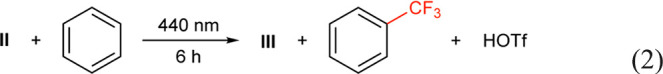
2

### Photocatalytic Arene C–H Trifluoromethylation

Addition of a ^+^CF_3_ fragment to the Co center in **III** affords **II** via a 2e^–^ process analogous to oxidative addition (Figure 1b). Accordingly, reactions with Umemoto’s *S*-(trifluoromethyl)dibenzothiophenium triflate (**1**) were pursued with the aim of closing a catalytic cycle for photocatalytic arene C–H trifluoromethylation.

A combination of **III** (5 mol %) with a 1:1 mixture of C_6_H_6_ and Umemoto’s *S*-(trifluoromethyl)dibenzothiophenium triflate (**1**) in MeCN afforded α,α,α-trifluorotoluene in 18% yield after 6 h of continuous irradiation using a 440 nm blue LED lamp (Table 1, entry 4). Major byproducts included trifluoromethylated dibenzothiophenes, which result from attack of the promiscuous ^•^CF_3_ radical on the heteroarene product of ^+^CF_3_ removal from **1**.^48^ Accordingly, increasing the ratio of C_6_H_6_ to **1** to 5:1 gave a 3-fold increase in yield of α,α,α-trifluorotoluene and significantly depressed the competitive dibenzothiophene trifluoromethylation (Table 1, entry 6). A maximum yield of α,α,α-trifluorotoluene was observed with a 10-fold excess of the C_6_H_6_ substrate (Table 1, entries 7 and 8). Control experiments performed without cobalt gave a maximum yield of 35% (Table 1, entries 1 and 2), but in all cases, the yield of α,α,α-trifluorotoluene is significantly increased by the addition of **III**. The use of CoCl_2_ in place of **III** gave a statistically insignificant increase in the measured yield relative to that under the metal-free conditions (Table 1, entry 3). Reactions performed in the dark with and without **III** gave no measurable α,α,α-trifluorotoluene (Table 1, entries 9 and 10).

**Table 1:**
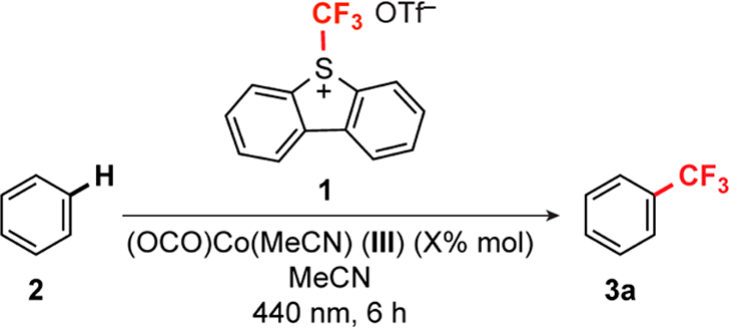
Optimization of Reaction Conditions

entry	catalyst (mol %)	**2** (equiv vs **1**)	yield (%)^a^
1	none	1	20
2	none	10	35
3	CoCl_2_ (5)	10	42
4	**III** (5)	1	18
5	**III** (5)	3	43
6	**III** (5)	5	58
7	**III** (5)	10	73
8	**III** (5)	20	73
9^b^	none	10	0
10^b^	**III** (5)	10	<1
11	**III** (5)	10	74

a Yields were determined by integration of ^19^F NMR resonances using C_6_F_6_ as an internal standard based on **1** as the limiting reagent, as described in the Supporting Information.

b Reaction performed in the dark.

**Figure 7:**
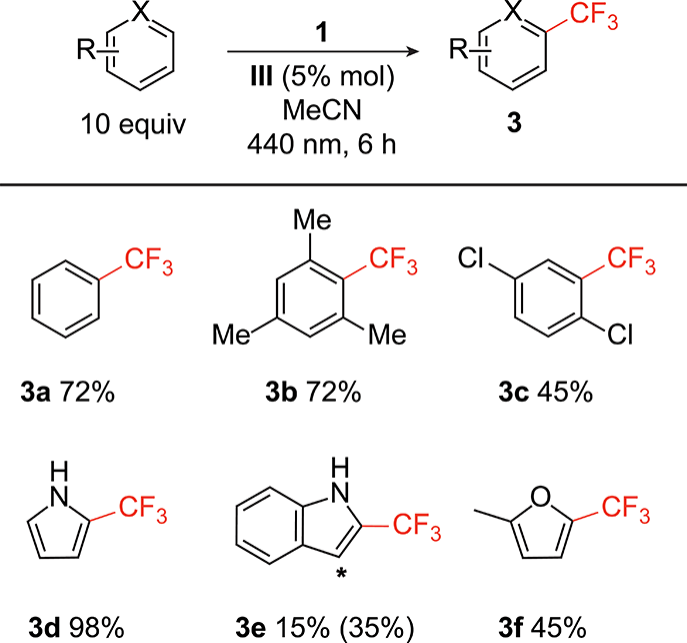
Photocatalytic trifluoromethylations of arenes and heteroarenes using **1**. Yields were determined by integration of ^19^F NMR resonances, as described in the Supporting Information. In cases where multiple isomers are formed, the overall yield is listed in parentheses.

The scope of the C–H trifluoromethylation was evaluated using six different (hetero)arene substrates (Figure 7). There was no significant change in yield when the arene ring was made more electron rich (**3b**); there was a significant decrease in yield when the ring was more electron poor (**3c**). Pyrrole (**3d**) exhibited high yield and excellent selectivity, with the only isomer formed being trifluoromethylation at the 2-position of the ring. Indole (**3e**) showed moderate reactivity but poor selectivity with an overall 35% yield and a 1:1 ratio between the 2- and 3-positions of the indole backbone.

**Scheme 2:**
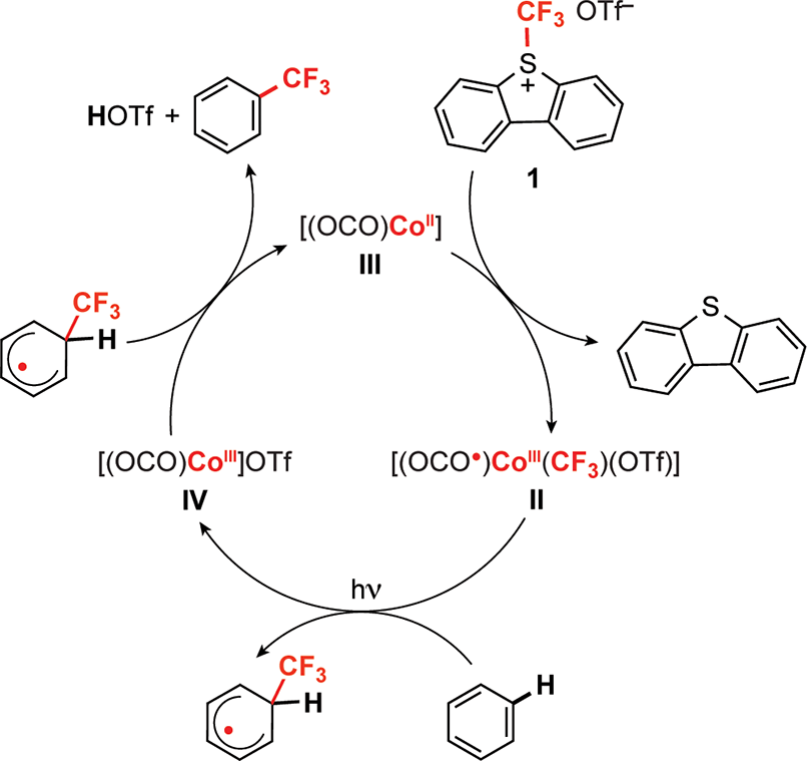
Experimentally Demonstrated Steps for Photoredox Trifluoromethylation of (Hetero)arenes Using **II** or **III** via Intermediate **IV**

A series of reactions was performed to probe the nature of the active catalyst. Treatment of a dark orange solution of **III** in CH_2_Cl_2_ with 1 equiv of **1** in the absence of light gave an immediate color change to dark olive-green. Monitoring the reaction by UV–vis spectroscopy showed the complete disappearance of diagnostic bands for **III** at 355 and 438 nm and the commensurate appearance of a spectrum with features at 451, 518, and 890 nm (Figure S3), which matches a 1:1 mixture of **II** and **IV**. The 50% yield of **II** in the stoichiometric reaction apparently results from the photodegradation of **II** during the synthesis, suggesting the combination of **III** + **1** is a path to photoactive **II** and providing entry to functional catalysis. Accordingly, mixing **III** with 20 equiv of **1** in a MeCN solution containing 200 equiv of C_6_H_6_ in the dark gave full consumption of the diagnostic CT bands for **III** and 80% conversion to **II** (Figure S4). Finally, irradiation of an acetonitrile solution containing **II** (5 mol %), **1**, and 10 equiv of C_6_H_6_ with 440 nm blue LED light gave α,α,α-trifluorotoluene in 74% yield over 6 h (Table 1, entry 11), suggesting that **III** and **II** give entry to the same catalytic cycle and **II** is an active intermediate for trifluoromethylation (Scheme 2).

## Discussion

The capacity of (OCO)Co(CF_3_) complexes to generate ^•^CF_3_ radical upon photoexcitation is predicated on the population of an MO that is primarily Co–CF_3_ σ* in nature. Accordingly, our previous successes in visible light-induced stoichiometric arene C–H trifluoromethylations using **I** utilized low energy LMCT from a ligand centered HOMO on the reduced form of the redox active [OCO]^2–^ ligand to a d_*z*^2^_-centered LUMO to weaken the Co–CF_3_ bond by reduction of the formal bond order to 0.5 and promote Co–CF_3_ homolysis.^35^ In theory, the 1e^–^ oxidation of **I** to a formal Co(IV) center in **II** should critically perturb multiple essential elements of this delicate framework. In practice, the redox plasticity afforded by the [OCO] ligand provides a robust mechanism to retain key design features and extend the stoichiometric photochemistry to a functional 2e^–^ photoredox catalytic cycle.

The Co cores of complexes **I** and **II** remain functionally unchanged upon oxidation. As in **I**, structural and computational data suggest that **II** is best formulated as a low-spin Co(III) center, and the computed LUMO is primarily Co–CF_3_ σ* antibonding. Accordingly, the Co^III^–CF_3_ bond in **II** is thermodynamically robust—it is indefinitely stable in the dark—but is photochemically activated by population of the lowest-lying unoccupied metal-centered orbital. The bias for a low-spin Co(III) center in the (OCO)Co(CF_3_) core drives formation of the [OCO^•^]^−^ ligand oxidation state in **II**, which was not observed in previous reports of closely related electron transfer series.^36^

Ligand-centered oxidation affects the photophysical properties of **II**. Both **I** and **II** feature [OCO] ligand-centered HOMOs of π symmetry, which are the loci of the oxidations in the photochemically active LMCT excited states. Electrostatic principles suggest that oxidation of the [OCO^•^]^−^ ligand in **II** should be significantly more challenging than that in the fully reduced [OCO]^2–^ ligand in **I**. This is evident in the absorption CT band that engenders the photochemical behavior, which is shifted from 738 nm in **I** to 438 nm in **II**. This shift likely also reflects differences in the coordination environment about cobalt. Square pyramidal **I** is deactivated by binding a sixth ligand *trans* to CF_3_ because the energy of the σ* d_*z*^2^_ MO is raised above d_*x*^2^__–*y*^2^_.^35^ The solid-state structure of **II** contains a ^–^OTf ligand in the sixth site, giving **II** quasi-octahedral geometry. Simple MO arguments would expect ^–^OTf coordination to similarly raise the energy of the photoactive d_*z*^2^_ LUMO, but this is insufficient to deactivate **II**. Computational data suggest that a d_*z*^2^_-like orbital is still the primary contributor to the LUMO in **II**. The possibility of equilibrium ^–^OTf dissociation generating a photochemically active five-coordinate species cannot be ruled out under the catalysis conditions, but the fact that **II** retains its photosensitivity in the MeCN solvent argues against deactivation in octahedral geometries. This makes **II** more versatile by giving access to a wider range of solvents and opening avenues for the trifluoromethylation of substrates that can act as strong σ donors to Co, as described below.

As a stoichiometric source of the free ^•^CF_3_ radical, **II** suffers in comparison to **I**. Under analogous conditions, the yield of α,α,α-trifluoromethyltoluene from C_6_H_6_ is reduced from >99% to 58%. The origin of this disparity is apparently an enhanced propensity of **II** to trap ^•^CF_3_ via C–C coupling to the ligand backbone, which subsequently induces demetalation and formation of unidentified Co byproducts (Scheme 1). No evidence for ligand centered ^•^CF_3_ radical coupling has been observed in the photolysis of **I**. It is tempting to ascribe this partitioning to the presence of unpaired spin on the [OCO^•^]^−^ ligand making **II** a more effective radical trap, but radical character on the arene is not a prerequisite for radical coupling, and the relative kinetics of the C–C bond forming reactions in the excited states are entirely unknown at this time. This flaw is not fatal, however. Competitive trapping at the ligand can be disfavored by lowering the concentration of **II** and increasing the relative concentration of the organic acceptor. That is, the conditions one would typically pursue in catalysis are exactly those required for high-yielding reactions with organic substrates.

**Scheme 3:**
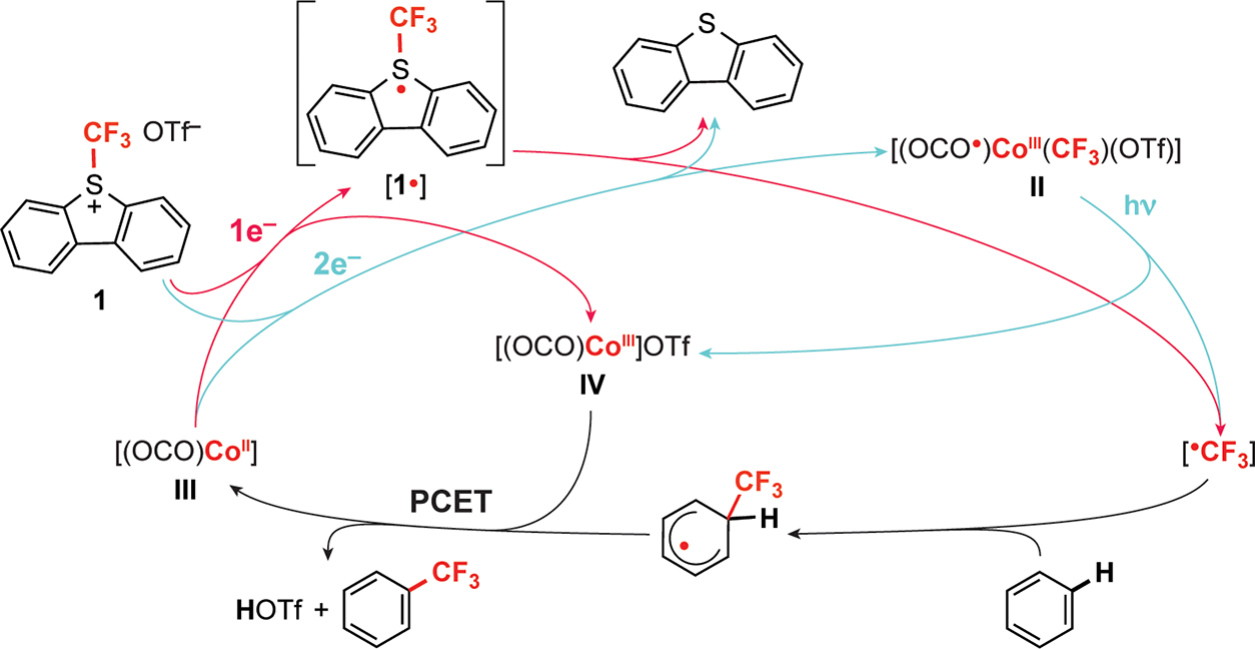
Proposed Photocatalysis Mechanisms of Arene Trifluoromethylation Using **III**

Clean conversion of **III** to **II** using **1** establishes all of the steps required for functional photocatalysis, as illustrated in Scheme 2. The use of **1** here demands a multielectron redox capacity that would typically limit the use of cobalt, as the thermodynamically preferred oxidation +2 and +3 states are incompatible with the formal 2e^–^ redox change that occurs upon ^+^CF_3_ addition to the metal center. The redox active [OCO] ligand sidesteps this issue by coupling 1e^–^ redox at cobalt with 1e^–^ oxidation at the ligand, as shown in the blue pathway in Scheme 3. The net 2e^–^ reaction can occur without accumulation of 1e^–^ intermediates because of strong electronic coupling and covalency in the Co–OCO bonding.^36^

Given its well-established propensity to participate in radical ^•^CF_3_ transfer, the use of **1** as a CF_3_ source merits additional discussion. Although **1** was initially conceived as a source of ^+^CF_3_, radical mechanisms have been frequently invoked.^49^ These suggest that initial 1e^–^ transfer generates a reduced form of Umemoto’s regent (1^•^ in Scheme 3), which is itself a source of ^•^CF_3_ radical. Accordingly, single electron transfer (SET) and radical chain mechanisms have been suggested for trifluoromethylations using **1** with common photoreductants or reducing metals in their ground state.^43,44,48,50−52^ Ground state initial electron transfer (ET), illustrated by the red 1e^–^ path in Scheme 3, is unlikely here based on the redox potentials of the reactants. Whereas **1** is reduced at −0.75 V vs Fc^+^/Fc,^51,52^ oxidation of **III** occurs at −0.32 V vs Fc^+^/Fc,^36^ implying initial outer-sphere 1e^–^ transfer is uphill by over 400 mV. Moreover, reaction of **III** with **1** in the dark in the presence of C_6_H_6_ gives exclusively **II**; ruling out 1^•^ as a viable route to trifluoromethylated arene in the absence of light.

Direct photoactivation of **1** has been reported and suggested to occur via *in situ* generation of π–π complexes with arenes, which are competent visible light chromophores.^53,54^ This may account for the significant background reactivity observed herein. To the best of our knowledge, the excited-state reduction potential of **1** is unknown. Accordingly, the red 1e^–^ pathway cannot be rigorously excluded as a contributor to the observed catalysis under constant photolysis.

Irradiation of **II** with visible light induces facile Co–CF_3_ bond homolysis to generate a persistent ^•^CF_3_ electrophile capable of attacking (hetero)arenes (Scheme 3). Rearomatization of the initially formed cyclohexadienyl radical requires net loss of H^•^. Formation of **III** in these reactions implies a net 2e^–^ reduction of the cobalt center, and UV–vis data are consistent with this occurring by two consecutive 1e^–^ steps via Co(III) intermediate **IV**. The role of **IV** is an outer-sphere 1e^–^ oxidant rather than an H^•^ acceptor. The net proton-coupled electron transfer (PCET) reaction is balanced by loss of H^+^ in the form of HOTf, which apparently generates dibenzothiophenium triflate under the catalysis conditions.^55^

Throughout this paper, the ligand *trans* to the carbene in the [OCO] ligand is largely ignored because it is a spectator. Work on these and closely related ET series have revealed a capacity to bind a range of O- and N-donor substrates, including furans, pyridines, and nitriles in this position.^35,36^ The photochemistry of **II** is seemingly unaffected by ligand substitution at this site. Accordingly, the selectivity reported herein is determined by the persistent ^•^CF_3_ radical and the arene substrates themselves. Trifluoromethylation of the [OCO] ligand demonstrates the capacity of an inner-sphere radical acceptor to function as a competitive trap, presumably via geminate recombination, suggesting that an oriented substrate should be similarly susceptible to aryl trifluoromethylation within the solvent cage.

## Conclusion

The novelty of the catalysis reported herein is not the organic products, the use of light to generate ^•^CF_3_ radical, or even the application of organocobalt(III) complexes for trifluoromethylation but rather extrapolation of the catalyst design principles for photolytic C–H arene trifluoromethylation to functional catalysis and the avenues for selective photoredox catalysis that result. Redox noninnocence of the [OCO] ligand is a central and essential feature of every step of the catalysis cycle. First, by imparting a multielectron redox capacity at Co, it permits the use of CF_3_ sources that demand formal 2e^–^ oxidation of the metal center to generate the active organometallic intermediate while circumventing high energy Co(IV) or Co(I) species. Second, the ligand redox flexibility preserves the low spin Co^III^–CF_3_ core across multiple formal oxidation states, which makes the complexes thermodynamically inert but photochemically labile. Finally, the capacity of the [OCO] framework to be oxidized at modest potentials provides a reservoir of accessible ligand-centered electrons for generation of the photochemically active LMCT state with low energy light. The result is a coordination complex that functions as a combined chromophore and organometallic reaction center for a visible light photoredox catalysis cycle, obviating the requirement for long excited-state lifetimes which often limit the use of base metals in photoredox catalysis. The VLIH design principles elaborated in this system are broadly transferable; our ongoing work is focused on extensions to other inexpensive sources of CF_3_, structural modifications to tune the absorption properties and photochemistry of the organometallic chromophores, and selectivity in radical C–H trifluoroalkylations of heteroarenes that derives from spatial proximity of the C–H bonds to the incipient ^•^CF_3_ rather than the substrate electronics.

## Experimental Section

### General Considerations

Unless otherwise mentioned, all operations were carried out under anaerobic conditions using standard vacuum line techniques or in an inert-atmosphere glovebox under nitrogen. All NMR spectra were recorded on a Varian Mercury 400 spectrometer and chemical shifts are reported in parts per million (ppm) relative to tetramethylsilane (TMS), with the residual solvent peak as an internal reference.^56^ All ^19^F chemical shifts are reported in ppm relative to CFCl_3_ with hexafluorobenzene as an internal standard. Solution magnetic moments were obtained by the Evans’ NMR method.^57,58^ UV–vis absorption spectra were acquired by using a Hitachi 4150 spectrophotometer. Unless otherwise noted, all electronic absorption spectra were recorded at ambient temperature in a 1 cm quartz cell. All mass spectra were recorded at the Georgia Institute of Technology Bioanalytical Mass Spectrometry Facility. Electrospray ionization mass spectrometry (ESI-MS) was carried out with acetonitrile solutions using a Micromass Quattro LC spectrometer. Elemental analyses were performed by Atlantic Microlab, Inc., Norcross, GA. All analyses were performed in duplicate, and the reported compositions are the averages of the two runs. Full details of X-ray data collection and refinement are provided in the Supporting Information.

### Materials and Methods

Anhydrous acetonitrile (MeCN), dichloromethane (CH_2_Cl_2_), benzene, tetrahydrofuran (THF), pentane, and toluene solvents for air- and moisture-sensitive manipulations were purchased from Sigma-Aldrich and further dried by passage through columns of activated alumina, degassed by at least three freeze–pump–thaw cycles, and stored under N_2_ prior to use. Hexamethyldisiloxane (HMDSO) was degassed by at least three freeze–pump–thaw cycles and stored over activated 4 Å molecular sieves under N_2_ prior to use. Methanol (Drisolv) was purchased from EMD Millapore and was used as received. Deuterated solvents were purchased from Cambridge Isotope Laboratories. Acetonitrile-*d*_3_, DCM-*d*_2_, and THF-*d*_8_ were placed an in oven-dried sealable flask and degassed by freeze–pump–thaw cycles and then stored over activated 4 Å molecular sieves under N_2_ prior to use. AgCF_3_ was prepared according to a published procedure.^59^ Silver fluoride (Strem), TMSCF_3_ (Oakwood), Umemoto’s reagent (Sigma), and hexafluorobenzene (Sigma) were all used as received. [(OCO)Co(THF)],^36^ [(OCO)Co(THF)_2_]OTf (**IV**),^36^ and [(OCO)Co(CF_3_)(MeCN)] (**I**)^35^ were prepared by published procedures.

#### Synthesis of [(OCO)CoCF_3_(THF)OTf] (**II**)

A 20 mL scintillation vial was charged with [(OCO)Co(CF_3_)(MeCN)] (**I**) (150 mg, 0.232 mmol) in DCM (5 mL). To this solution, a suspension of AgOTf (63 mg, 0.25 mmol) in DCM (2 mL) was added dropwise, and an immediate color change from burgundy red to dark green occurred. After stirring for 3 h, the reaction as was filtered through a 2 mm pad of Celite and washed with DCM until the washings were no longer colored. The filtrate was concentrated *in vacuo* affording a dark olive-green colored solid **II** (180 mg, 0.226 mmol, 98%). X-ray suitable crystals were grown from a concentrated 1:1 (THF:HMDSO) solution that was evaporated over 2 days, resulting in dark-green plates. Satisfactory elemental analysis required the inclusion of one H_2_O, which has been observed in structures obtained from wet solvents; the reported analysis is for [(OCO)Co(CF_3_)(MeCN)(OH_2_)]OTf. Anal. Calc. for C_35_H_49_CoF_6_N_3_O_6_S: C, 51.72; H, 6.08; N, 5.17; Found: C, 52.10; H, 6.22; N, 4.82. HR-ESI-MS (*m*/*z*): 604.2663 [M-THF-OTf]^+^. UV–vis (DCM) λ_max_, nm (ε, M^–1^cm^–1^): 442 (6672), 461 (6475), 523 (2411), 895 (4550).

#### C–H Trifluoromethylation of (Hetero)Aryls

In a representative procedure, a borosilicate NMR tube with a J. Young valve was charged with **1** (80.5 mg, 0.2 mmol), C_6_H_6_ (180 μL, 2 mmol, 10 equiv), **III** (5 mg, 0.01 mmol, 5% mol), and CD_3_CN (1 mL). The tube was sealed and placed ∼3 in. from a Kessil KSPR 160L-440 LED lamp for 6 h. Hexafluorobenzene (11.5 μL, 0.1 mmol) was added as an internal standard, and yields were measured by integration against the ^19^F resonances for the CF_3_ containing products. The NMR spectra matched those previously reported.^14,44,50,60−62^

### Computational Studies

DFT calculations were performed using ORCA 4.2.1^63,64^ using the BP86^65,66^ functional and def2-TZVP^67,68^ basis set (default grid) on the full model. TD-DFT calculations were performed at the same level of theory using the output coordinates from the geometry optimization as input. Spin density, difference density, and molecular orbital plots were generated using IQmol (http://www.iqmol.org/) and IboView (http://www.iboview.org).

## Supporting Information Available

Details of photolysis apparatus; UV–vis absorption data for thermal stability study of **II** and selected reactions of **III** with **1**; ^19^F NMR spectra for stoichiometric photolysis reactions of **II** and catalytic reactions with **1** and/or **III** and substrates; ESI–MS data for **II** and a photolysis reaction of **II** with TEMPO^•^; selected X-ray crystallographic data for **II**, **III**, and [(OCO^0^)Co^II^(THF)_3_](PF_6_)_2_; crystal data and structure refinement for **II**; DFT Orca input files and orbitals and TDDFT transitions for **II** (PDF)
Complete X-ray structure report for **II** (CIF)

## Supplementary Material

cs3c03832_si_001.pdf

cs3c03832_si_002.cif

## References

[ref1] Hagmann W. K. The many roles for fluorine in medicinal chemistry. J. Med. Chem. 2008, 51, 4359–4369. 10.1021/jm800219f. 18570365

[ref2] Purser S.; Moore P. R.; Swallow S.; Gouverneur V. Fluorine in medicinal chemistry. Chem. Soc. Rev. 2008, 37, 320–330. 10.1039/B610213C. 18197348

[ref3] Jeschke P. The unique role of halogen substituents in the design of modern agrochemicals. Pest Management Science 2010, 66, 10–27. 10.1002/ps.1829. 19701961

[ref4] Wang J.; Sanchez-Rosello M.; Acena J. L.; del Pozo C.; Sorochinsky A. E.; Fustero S.; Soloshonok V. A.; Liu H. Fluorine in Pharmaceutical Industry: Fluorine-Containing Drugs Introduced to the Market in the Last Decade (2001–2011). Chem. Rev. 2014, 114, 2432–2506. 10.1021/cr4002879. 24299176

[ref5] Meanwell N. A. Fluorine and Fluorinated Motifs in the Design and Application of Bioisosteres for Drug Design. J. Med. Chem. 2018, 61, 5822–5880. 10.1021/acs.jmedchem.7b01788. 29400967

[ref6] Intermaggio N. E.; Millet A.; Davis D. L.; MacMillan D. W. C. Deoxytrifluoromethylation of Alcohols. J. Am. Chem. Soc. 2022, 144, 11961–11968. 10.1021/jacs.2c04807. 35786873 PMC9676087

[ref7] Prakash G. K. S.; Yudin A. K. Perfluoroalkylation with organosilicon reagents. Chem. Rev. 1997, 97, 757–786. 10.1021/cr9408991. 11848888

[ref8] Liu X.; Xu C.; Wang M.; Liu Q. Trifluoromethyltrimethylsilane: Nucleophilic Trifluoromethylation and Beyond. Chem. Rev. 2015, 115, 683–730. 10.1021/cr400473a. 24754488

[ref9] Umemoto T. Electrophilic perfluoroalkylating agents. Chem. Rev. 1996, 96, 1757–1777. 10.1021/cr941149u. 11848810

[ref10] Charpentier J.; Fruh N.; Togni A. Electrophilic Trifluoromethylation by Use of Hypervalent Iodine Reagents. Chem. Rev. 2015, 115, 650–682. 10.1021/cr500223h. 25152082

[ref11] Studer A. A ″Renaissance″ in Radical Trifluoromethylation. Angew. Chem. Int. Ed 2012, 51, 8950–8958. 10.1002/anie.201202624. 22890985

[ref12] Gurry M.; Aldabbagh F. A new era for homolytic aromatic substitution: replacing Bu3SnH with efficient light-induced chain reactions. Org. Biomol. Chem. 2016, 14, 3849–62. 10.1039/C6OB00370B. 27056571

[ref13] Duncton M. A. J. Minisci reactions: Versatile CH-functionalizations for medicinal chemists. Medchemcomm 2011, 2, 1135–1161. 10.1039/c1md00134e.

[ref14] Nagib D. A.; MacMillan D. W. C. Trifluoromethylation of arenes and heteroarenes by means of photoredox catalysis. Nature 2011, 480, 224–228. 10.1038/nature10647. 22158245 PMC3310175

[ref15] Ji Y. N.; Brueckl T.; Baxter R. D.; Fujiwara Y.; Seiple I. B.; Su S.; Blackmond D. G.; Baran P. S. Innate C-H trifluoromethylation of heterocycles. Proc. Natl. Acad. Sci. U.S.A. 2011, 108, 14411–14415. 10.1073/pnas.1109059108. 21844378 PMC3167544

[ref16] Koike T.; Akita M. Trifluoromethylation by Visible-Light-Driven Photoredox Catalysis. Top. Catal. 2014, 57, 967–974. 10.1007/s11244-014-0259-7.

[ref17] Romero N. A.; Nicewicz D. A. Organic Photoredox Catalysis. Chem. Rev. 2016, 116, 10075–10166. 10.1021/acs.chemrev.6b00057. 27285582

[ref18] Michelin C.; Hoffmann N. Photosensitization and Photocatalysis—Perspectives in Organic Synthesis. ACS Catal. 2018, 8, 12046–12055. 10.1021/acscatal.8b03050.

[ref19] Shaw M. H.; Twilton J.; MacMillan D. W. C. Photoredox Catalysis in Organic Chemistry. J. Org. Chem. 2016, 81, 6898–6926. 10.1021/acs.joc.6b01449. 27477076 PMC4994065

[ref20] Hughes R. P. Organo-transition metal compounds containing perfluorinated ligands. In Advances in organometallic chemistry; Elsevier: 1990; Vol. 31, pp 183–267; 10.1016/S0065-3055(08)60511-0.

[ref21] Huang D. J.; Koren P. R.; Folting K.; Davidson E. R.; Caulton K. G. Facile and reversible cleavage of C-F bonds. Contrasting thermodynamic selectivity for Ru-CF2H vs F-Os=CFH. J. Am. Chem. Soc. 2000, 122, 8916–8931. 10.1021/ja001646u.

[ref22] Huang D.; Caulton K. G. New Entries to and New Reactions of Fluorocarbon Ligands. J. Am. Chem. Soc. 1997, 119, 3185–3186. 10.1021/ja963903u.

[ref23] Tomashenko O. A.; Grushin V. V. Aromatic Trifluoromethylation with Metal Complexes. Chem. Rev. 2011, 111, 4475–4521. 10.1021/cr1004293. 21456523

[ref24] Bour J. R.; Camasso N. M.; Sanford M. S. Oxidation of Ni(II) to Ni(IV) with Aryl Electrophiles Enables Ni-Mediated Aryl-CF3 Coupling. J. Am. Chem. Soc. 2015, 137, 8034–8037. 10.1021/jacs.5b04892. 26079544

[ref25] Liu L.; Xi Z. Organocopper(III) Compounds with Well-defined Structures Undergo Reductive Elimination to Form C—C or C-Heteroatom Bonds. Chin. J. Chem. 2018, 36, 1213–1221. 10.1002/cjoc.201800365.

[ref26] Liu S.; Liu H.; Liu S.; Lu Z.; Lu C.; Leng X.; Lan Y.; Shen Q. C(sp3)-CF3 Reductive Elimination from a Five-Coordinate Neutral Copper(III) Complex. J. Am. Chem. Soc. 2020, 142, 9785–9791. 10.1021/jacs.0c03304. 32365294

[ref27] Ackerman-Biegasiewicz L. K. G.; Kariofillis S. K.; Weix D. J. Multimetallic-Catalyzed C-C Bond-Forming Reactions: From Serendipity to Strategy. J. Am. Chem. Soc. 2023, 145, 6596–6614. 10.1021/jacs.2c08615. 36913663 PMC10163949

[ref28] Demarteau J.; Debuigne A.; Detrembleur C. Organocobalt Complexes as Sources of Carbon-Centered Radicals for Organic and Polymer Chemistries. Chem. Rev. 2019, 119, 6906–6955. 10.1021/acs.chemrev.8b00715. 30964644

[ref29] McCusker J. K. Electronic structure in the transition metal block and its implications for light harvesting. Science 2019, 363, 484–488. 10.1126/science.aav9104. 30705184

[ref30] Arias-Rotondo D. M.; McCusker J. K. The photophysics of photoredox catalysis: a roadmap for catalyst design. Chem. Soc. Rev. 2016, 45, 5803–5820. 10.1039/C6CS00526H. 27711624

[ref31] Wenger O. S. Is Iron the New Ruthenium?. Chem.—Eur. J. 2019, 25, 6043–6052. 10.1002/chem.201806148. 30615242

[ref32] Larsen C. B.; Braun J. D.; Lozada I. B.; Kunnus K.; Biasin E.; Kolodziej C.; Burda C.; Cordones A. A.; Gaffney K. J.; Herbert D. E. Reduction of Electron Repulsion in Highly Covalent Fe-Amido Complexes Counteracts the Impact of a Weak Ligand Field on Excited-State Ordering. J. Am. Chem. Soc. 2021, 143, 20645–20656. 10.1021/jacs.1c06429. 34851636

[ref33] Wenger O. S. Photoactive Complexes with Earth-Abundant Metals. J. Am. Chem. Soc. 2018, 140, 13522–13533. 10.1021/jacs.8b08822. 30351136

[ref34] Abderrazak Y.; Bhattacharyya A.; Reiser O. Visible-Light-Induced Homolysis of Earth-Abundant Metal-Substrate Complexes: A Complementary Activation Strategy in Photoredox Catalysis. Angew. Chem. Int. Ed 2021, 60, 21100–21115. 10.1002/anie.202100270. PMC8519011 33599363

[ref35] Harris C. F.; Kuehner C. S.; Bacsa J.; Soper J. D. Photoinduced Cobalt(III)-Trifluoromethyl Bond Activation Enables Arene C-H Trifluoromethylation. Angew. Chem. Int. Ed 2018, 57, 1311–1315. 10.1002/anie.201711693. 29240988

[ref36] Harris C. F.; Bayless M. B.; van Leest N. P.; Bruch Q. J.; Livesay B. N.; Bacsa J.; Hardcastle K. I.; Shores M. P.; de Bruin B.; Soper J. D. Redox-Active Bis(phenolate) N-Heterocyclic Carbene [OCO] Pincer Ligands Support Cobalt Electron Transfer Series Spanning Four Oxidation States. Inorg. Chem. 2017, 56, 12421–12435. 10.1021/acs.inorgchem.7b01906. 28968088

[ref37] Yang J. J.; Kirchmeier R. L.; Shreeve J. M. New electrophilic trifluoromethylating agents. J. Org. Chem. 1998, 63, 2656–2660. 10.1021/jo972213l. 11672133

[ref38] Eisenberger P.; Gischig S.; Togni A. Novel 10-I-3 hypervalent iodine-based compounds for electrophilic trifluoromethylation. Chem.—Eur. J. 2006, 12, 2579–2586. 10.1002/chem.200501052. 16402401

[ref39] Kieltsch I.; Eisenberger P.; Togni A. Mild electrophilic trifluoromethylation of carbon- and sulfur-centered nucleophiles by a hypervalent iodine(III)-CF3 reagent. Angew. Chem. Int. Ed 2007, 46, 754–757. 10.1002/anie.200603497. 17154193

[ref40] Matsnev A.; Noritake S.; Nomura Y.; Tokunaga E.; Nakamura S.; Shibata N. Efficient Access to Extended Yagupolskii-Umemoto-Type Reagents: Triflic Acid Catalyzed Intramolecular Cyclization of ortho-Ethynylaryltrifluoromethylsulfanes. Angew. Chem. Int. Ed 2010, 49, 572–576. 10.1002/anie.200905225. 20014375

[ref41] Liang T.; Neumann C. N.; Ritter T. Introduction of Fluorine and Fluorine-Containing Functional Groups. Angew. Chem. Int. Ed 2013, 52, 8214–8264. 10.1002/anie.201206566. 23873766

[ref42] Beatty J. W.; Douglas J. J.; Cole K. P.; Stephenson C. R. J. A scalable and operationally simple radical trifluoromethylation. Nat. Commun. 2015, 6, 7919 10.1038/ncomms8919. 26258541 PMC4533119

[ref43] Jacquet J.; Blanchard S.; Derat E.; Desage-El Murr M.; Fensterbank L. Redox-ligand sustains controlled generation of CF3 radicals by well-defined copper complex. Chemical Science 2016, 7, 2030–2036. 10.1039/C5SC03636D. 29899928 PMC5968567

[ref44] Meucci E. A.; Nguyen S. N.; Camasso N. M.; Chong E.; Ariafard A.; Canty A. J.; Sanford M. S. Nickel(IV)-Catalyzed C-H Trifluoromethylation of (Hetero)arenes. J. Am. Chem. Soc. 2019, 141, 12872–12879. 10.1021/jacs.9b06383. 31379153 PMC7542544

[ref45] King H. F.; Stanton R. E.; Kim H.; Wyatt R. E.; Parr R. G. Corresponding Orbitals and the Nonorthogonality Problem in Molecular Quantum Mechanics. J. Chem. Phys. 1967, 47, 1936–1941. 10.1063/1.1712221.

[ref46] Neese F. Definition of corresponding orbitals and the diradical character in broken symmetry DFT calculations on spin coupled systems. J. Phys. Chem. Solids 2004, 65, 781–785. 10.1016/j.jpcs.2003.11.015.

[ref47] Dang T. T.; Boeck F.; Hintermann L. Hidden Bronsted Acid Catalysis: Pathways of Accidental or Deliberate Generation of Triflic Acid from Metal Triflates. J. Org. Chem. 2011, 76, 9353–9361. 10.1021/jo201631x. 22010906

[ref48] Wang B.; Xiong D.-C.; Ye X.-S. Direct C-H Trifluoromethylation of Glycals by Photoredox Catalysis. Org. Lett. 2015, 17, 5698–5701. 10.1021/acs.orglett.5b03016. 26562610

[ref49] Wang S.-M.; Han J.-B.; Zhang C.-P.; Qin H.-L.; Xiao J.-C. An overview of reductive trifluoromethylation reactions using electrophilic ‘+CF3′ reagents. Tetrahedron 2015, 71, 7949–7976. 10.1016/j.tet.2015.06.056.

[ref50] Deolka S.; Govindarajan R.; Khaskin E.; Fayzullin R. R.; Roy M. C.; Khusnutdinova J. R. Photoinduced Trifluoromethylation of Arenes and Heteroarenes Catalyzed by High-Valent Nickel Complexes. Angew. Chem. Int. Ed 2021, 60, 24620–24629. 10.1002/anie.202109953. 34477296

[ref51] Koike T.; Akita M. Fine Design of Photoredox Systems for Catalytic Fluoromethylation of Carbon-Carbon Multiple Bonds. Acc. Chem. Res. 2016, 49, 1937–1945. 10.1021/acs.accounts.6b00268. 27564676

[ref52] Mizuta S.; Verhoog S.; Wang X.; Shibata N.; Gouverneur V.; Médebielle M. Redox chemistry of trifluoromethyl sulfonium salts as CF3 radical sources. J. Fluorine Chem. 2013, 155, 124–131. 10.1016/j.jfluchem.2013.07.006.

[ref53] Spell M. L.; Deveaux K.; Bresnahan C. G.; Bernard B. L.; Sheffield W.; Kumar R.; Ragains J. R. A Visible-Light-Promoted O-Glycosylation with a Thioglycoside Donor. Angew. Chem., Int. Ed. 2016, 55, 6515–6519. 10.1002/anie.201601566. 27086646

[ref54] Egami H.; Ito Y.; Ide T.; Masuda S.; Hamashima Y. Simple Photo-Induced Trifluoromethylation of Aromatic Rings. Synthesis 2018, 50, 2948–2953. 10.1055/s-0037-1609759.

[ref55] Al-Degs Y. S.; Al-Ghouti M. A. Influence of diesel acidification on dibenzothiophene removal: A new desulfurization practice. Sep. Purif. Technol. 2015, 139, 1–4. 10.1016/j.seppur.2014.10.027.

[ref56] Fulmer G. R.; Miller A. J. M.; Sherden N. H.; Gottlieb H. E.; Nudelman A.; Stoltz B. M.; Bercaw J. E.; Goldberg K. I. NMR Chemical Shifts of Trace Impurities: Common Laboratory Solvents, Organics, and Gases in Deuterated Solvents Relevant to the Organometallic Chemist. Organometallics 2010, 29, 2176–2179. 10.1021/om100106e.

[ref57] Evans D. F. The Determination of the Paramagnetic Susceptibility of Substances in Solution by Nuclear Magnetic Resonance. J. Chem. Soc. 1959, 2003–2005. 10.1039/jr9590002003.

[ref58] Piguet C. Paramagnetic susceptibility by NMR: The ’’solvent correction’’ removed for large paramagnetic molecules. J. Chem. Educ. 1997, 74, 815–816. 10.1021/ed074p815.

[ref59] Tyrra W. E. Oxidative perfluoroorganylation methods in group 12–16 chemistry - The reactions of haloperfluoroorganics and In and InBr, a convenient new route to AgRf (R-f = CF3, C6F5) and reactions of AgRf with group 12–16 elements. J. Fluorine Chem. 2001, 112, 149–152. 10.1016/S0022-1139(01)00484-5.

[ref60] Tanabe Y.; Matsuo N.; Ohno N. Direct Perfluoroalkylation Including Trifluoromethylation of Aromatics with Perfluoro Carboxylic-Acids Mediated by Xenon Difluoride. J. Org. Chem. 1988, 53, 4582–4585. 10.1021/jo00254a033.

[ref61] Ong J.; Loke J. W. L.; Koh H. L.; Fan W. Y. Proflavine-catalysed trifluoromethylation of α,β-unsaturated carbonyls. Molecular Catalysis 2022, 530, 112587 10.1016/j.mcat.2022.112587.

[ref62] Singh K.; Singh R.; Hazari A. S.; Adhikari D. Bimodal photocatalytic behaviour of a zinc beta-diketiminate: application to trifluoromethylation reactions. Chem. Commun. 2022, 58, 4384–4387. 10.1039/D2CC00397J. 35297908

[ref63] Neese F. The ORCA program System. Wiley Interdisciplinary Reviews: Computational Molecular Science 2012, 2, 73–78. 10.1002/wcms.81.

[ref64] Neese F. Software update: the ORCA program system, version 4.0. Wiley Interdisciplinary Reviews: Computational Molecular Science 2018, 8, e1327 10.1002/wcms.1327.

[ref65] Perdew J. P. Density-Functional Approximation for the Correlation-Energy of the Inhomogeneous Electron-Gas. Phys. Rev. B 1986, 33, 8822–8824. 10.1103/PhysRevB.33.8822. 9938299

[ref66] Becke A. D. Density-Functional Exchange-Energy Approximation with Correct Asymptotic-Behavior. Phys. Rev. A 1988, 38, 3098–3100. 10.1103/PhysRevA.38.3098. 9900728

[ref67] Weigend F.; Haser M.; Patzelt H.; Ahlrichs R. RI-MP2: optimized auxiliary basis sets and demonstration of efficiency. Chem. Phys. Lett. 1998, 294, 143–152. 10.1016/S0009-2614(98)00862-8.

[ref68] Weigend F.; Ahlrichs R. Balanced basis sets of split valence, triple zeta valence and quadruple zeta valence quality for H to Rn: Design and assessment of accuracy. Phys. Chem. Chem. Phys. 2005, 7, 3297–3305. 10.1039/b508541a. 16240044

