# Supplementary material for: Catalytic C–H Trifluoromethylation of Arenes and Heteroarenes via Visible Light Photoexcitation of a Co(III)–CF3 Complex
Source: ACS Catal. 2023 Oct 9;13(20):13607–17. doi: 10.1021/acscatal.3c03832 (PMC10594583; doi:10.1021/acscatal.3c03832)
Supplement: Supplementary file 1 — cs3c03832_si_001.pdf [file cs3c03832_si_001.pdf]

Supporting Information for:

Catalytic C–H Trifluoromethylation of Arenes and Heteroarenes via Visible Light Photoexcitation of a Co(III)–CF_3 Complex

Christopher S. Kuehner[a], Andrew G. Hill[a], Caleb F. Harris[a], Christian A. Owens,[a] John Bacsa[a,b], and Jake D. Soper*[,a]

[a]School of Chemistry and Biochemistry, Georgia Institute of Technology, Atlanta, Georgia 30332-0400, United States

[b]X-ray Crystallography Center, Department of Chemistry, Emory University, 1515 Dickey Drive, Atlanta, Georgia 30322, United States

Email: jake.soper@gatech.edu

Table of Contents

Experimental Details

**Figure S1**	Standard reactor setup for C–H trifluoromethylation of aryls	**S4**

UV-vis Data **S5-S7**

**Figure S2**	Absorption spectra for [(OCO•)Co^{III}(CF_3)(THF)OTf] (**II**) in CH_2Cl_2 heated to 50 °C.	**S5**
**Figure S3**	Absorption spectrum for the stoichiometric reaction of Umemoto's S-(trifluoromethyl)dibenzothiophenium triflate (**1**) and (OCO)Co^{II}(THF) (**III**) in CH_2Cl_2.	**S6**
**Figure S4**	Absorption spectra of the optimized catalytic conditions containing **III**, **1**, and C_6H_6 in CH_3CN.	**S7**

Stoichiometric C–H Trifluoromethylation NMR Data **S8-S13**

**Figure S5**	^{19}F NMR spectrum of **II** with 10 eq. TEMPO• in 1 mL CH_2Cl_2 after 6 h exposure to 440 nm light.	**S8**
**Figure S6**	^{19}F NMR spectrum of **II** with 10 eq. TEMPO• in 2 mL CH_2Cl_2 after 6 h exposure to 440 nm light.	**S9**
**Figure S7**	^{19}F NMR spectrum of **II** with 15 eq. TEMPO• in 1 mL CH_2Cl_2 after 6 h exposure to 440 nm light.	**S10**
**Figure S8**	^{19}F NMR spectrum of **II** with 5 eq. C_6H_6 in 1 mL CH_2Cl_2 after 6 h exposure to 440 nm light.	**S11**
**Figure S9**	^{19}F NMR spectrum of **II** in C_6H_6 after 6 h exposure to 440 nm light.	**S12**
**Figure S10**	^{19}F NMR spectrum of **II**, and *N,N*-diisopropylethylamine in C_6H_6 after 6 h exposure to 440 nm light.	**S13**

Catalytic C–H Trifluoromethylation Optimization NMR Data S14-S23

Figure S11 — ^{19}F NMR spectrum of **1** with 1 eq. C_6H_6 in 1 mL CD_3CN after 6 h exposure to 440 nm light. — S14

Figure S12 — ^{19}F NMR spectrum of **1** with 10 eq. C_6H_6 in 1 mL CD_3CN after 6 h exposure to 440 nm light. — S15

Figure S13 — ^{19}F NMR spectrum of **1** with 10 eq. C_6H_6 in 1 mL CD_3CN after 6 h in the dark. — S16

Figure S14 — ^{19}F NMR spectrum of **1** with 10 eq. C_6H_6 and 5% mol **III** in 1 mL CD_3CN after 6 h in the dark. — S17

Figure S15 — ^{19}F NMR spectrum of **1** with 10 eq. C_6H_6 and 5% mol $CoCl_2$ in 1 mL CD_3CN after 6 h exposure to 440 nm light. — S18

Figure S16 — ^{19}F NMR spectrum of **1** with 1 eq. C_6H_6 and 5% mol **III** in 1 mL CD_3CN after 6 h exposure to 440 nm light. — S19

Figure S17 — ^{19}F NMR spectrum of **1** with 3 eq. C_6H_6 and 5% mol **III** in 1 mL CD_3CN after 6 h exposure to 440 nm light. — S20

Figure S18 — ^{19}F NMR spectrum of **1** with 5 eq. C_6H_6 and 5% mol **III** in 1 mL CD_3CN after 6 h exposure to 440 nm light. — S21

Figure S19 — ^{19}F NMR spectrum of **1** with 10 eq. C_6H_6 and 5% mol **III** in 1 mL CD_3CN after 6 h exposure to 440 nm light. — S22

Figure S20 — ^{19}F NMR spectrum of **1** with 20 eq. C_6H_6 and 5% mol **III** in 1 mL CD_3CN after 6 h exposure to 440 nm light. — S23

Catalytic C–H Trifluoromethylation Substrate Scope NMR Data S24-S29

Figure S21 — ^{19}F NMR spectrum of **1** with 10 eq. pyrrole and 0.5% mol **III** in 1 mL CD_3CN after 6 h exposure to 440 nm light. — S24

Figure S22 — ^{19}F NMR spectrum of **1** with 10 eq. indole and 0.5% mol **III** in 1 mL CD_3CN after 6 h exposure to 440 nm light. — S25

Figure S23 — ^{19}F NMR spectrum of **1** with 10 eq. 2-methylfuran and 0.5% mol **III** in 1 mL CD_3CN after 6 h exposure to 440 nm light. — S26

Figure S24 — ^{19}F NMR spectrum of **1** with 10 eq. mesitylene and 0.5% mol **III** in 1 mL CD_3CN after 6 h exposure to 440 nm light. — S27

**Figure S25**	^{19}F NMR spectrum of **1** with 10 eq. 1,4-dichlorobenzene and 0.5% mol **III** in 1 mL CD_3CN after 6 h exposure to 440 nm light.	**S28**
**Figure S26**	^{19}F NMR spectrum of **1** with 10 eq. C_6H_6 and 0.5% mol **II** in 1 mL CD_3CN after 6 h exposure to 440 nm light.	**S29**
**ESI-MS Data**		**S30-S31**
**Figure S27**	HR-ESI-MS of isolated **II**	**S30**
**Figure S28**	HR-ESI-MS of **II** with 10 eq. TEMPO• in 1 mL CH_2Cl_2 after 6 h exposure to 440 nm light.	**S31**
**X-Ray Crystallographic Data**		**S32-S33**
**Figure S29**	Comparison of selected bond lengths [Å] for **II** vs. the arithmetic mean of **III** and $[(OCO^0)Co^{II}(THF)_3](PF_6)_2$.	**S32**
**Figure S30**	Selected (OCO) ligand bond lengths [Å] for **II** and $[(OCO^0)Co^{II}(THF)_3](PF_6)_2$.	**S33**
**X-Ray Structure Report**		**S34-S44**
**$[(OCO•)Co^{III}(CF_3)(THF)OTf]$ II**		**S34**
**DFT ORCA Input Files**		**S45-S53**
**List S1**	DFT geometry optimization in the doublet state of **II**	**S45**
**List S2**	DFT geometry optimization in the quartet state of **II**	**S48**
**List S3**	Spin density and molecular orbital plots of **II** from DFT optimized coordinates	**S51**
**TDDFT**		**S54-S61**
**List S4**	ORCA input file for the TDDFT excited state calculation of **II** from DFT optimized geometry	**S54**
**Figure S31**	DFT molecular orbitals of **II**	**S57**
**List S5**	TDDFT transitions for **II**	**S60**
**References**		**S62**

Experimental Details

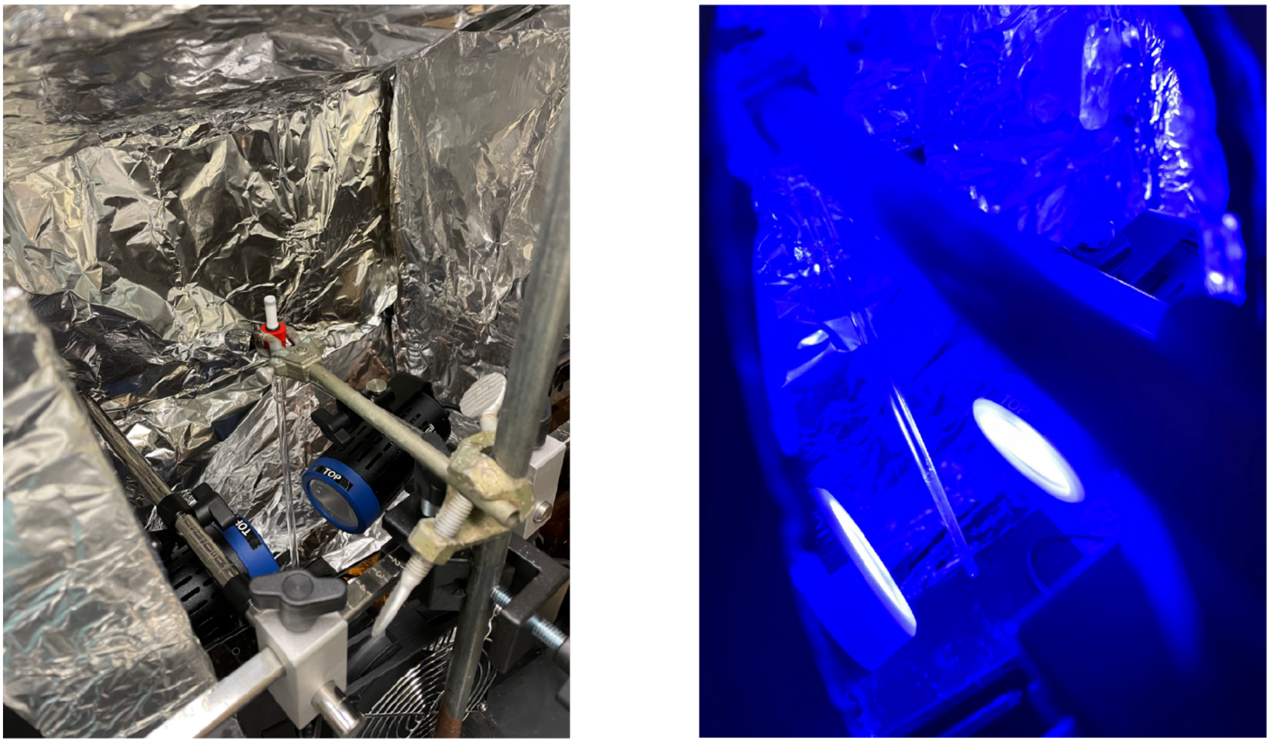

Figure S1. Left: inside ‘reactor‘ set-up; a fan introduces air through the front of the aluminum foil box to cool the reactions. Right: reaction in progress illuminated by two Kessil PR160L-440nm lamps (34 W).

UV-vis Data

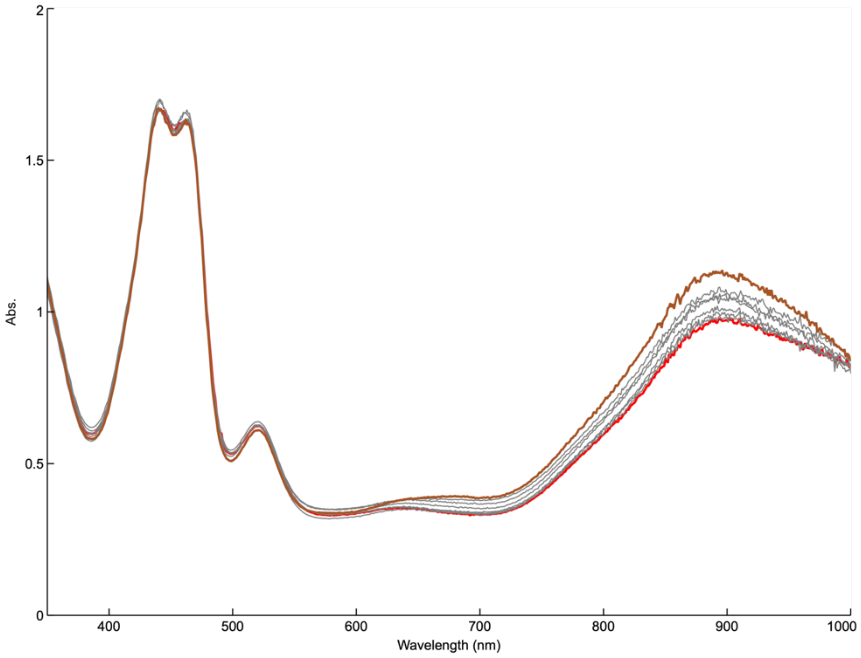

Figure S2. UV-vis absorption spectra for 2.5 x 10^{-4} M [(OCO•)Co^{III}(CF_3)(THF)OTf] (**II**) in CH_2Cl_2 at 50 °C. Spectra are shown at *t* = 0 (brown line) and 1 h intervals to *t* = 6 h (red line).

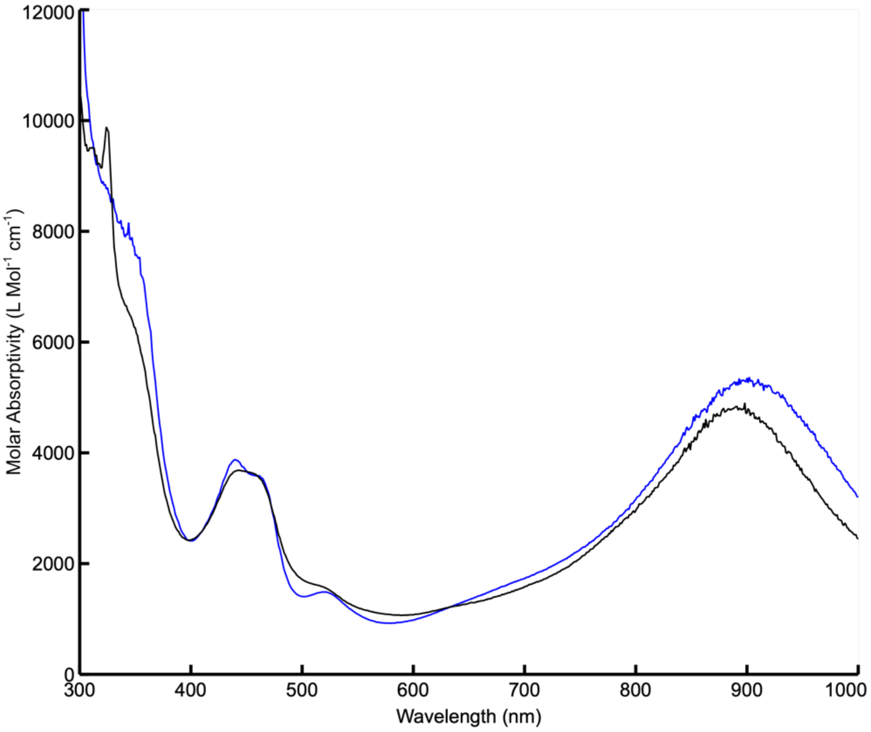

Figure S3. UV-vis absorption spectrum for 1.26 x 10^{-4} M CH_2Cl_2 solution from the crude reaction mixture of (OCO)Co^{II}(THF) (**III**) and 1 eq. of Umemoto's S-(trifluoromethyl)dibenzothiophenium triflate (**1**) in CH_2Cl_2 (black line). A 1:1 ([(OCO)$Co^{III}$$(THF)_2$]OTf (**IV**): **II** (blue line) in CH_2Cl_2 is shown for comparison.

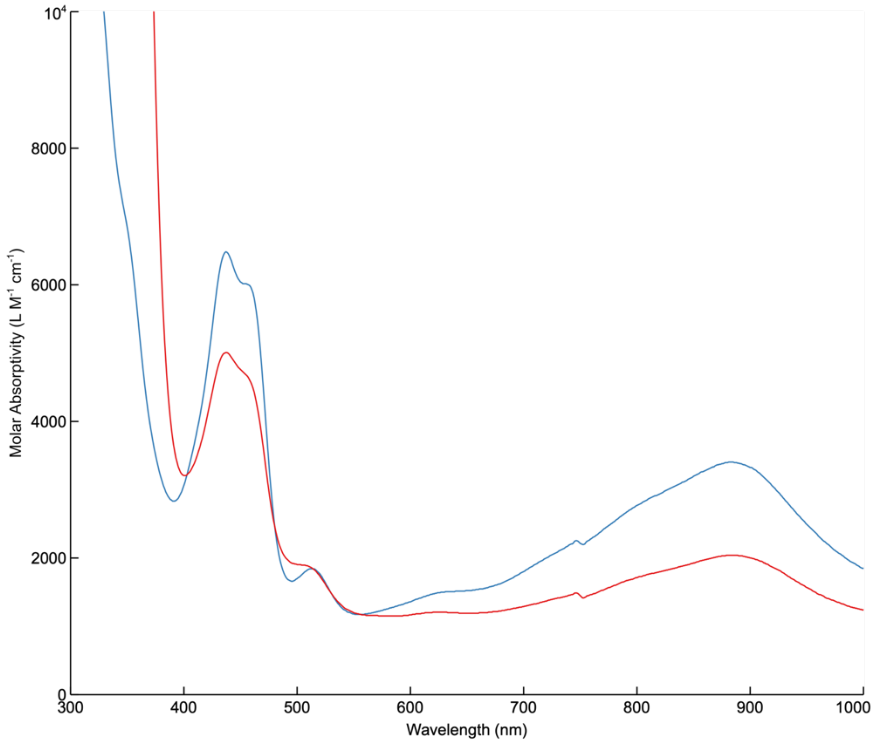

Figure S4. UV-vis absorption spectrum for a 1.25 x 10^{-4} M CH_3CN solution from the crude reaction mixture of 0.01 mmol **III**, 0.2 mmol **1**, and 2 mmol C_6H_6 in 1 mL of CH_3CN after 1 h of stirring. A spectrum of **II** (blue line) independently synthesized in CH_3CN is shown for comparison.

Stoichiometric C–H Trifluoromethylation NMR Data

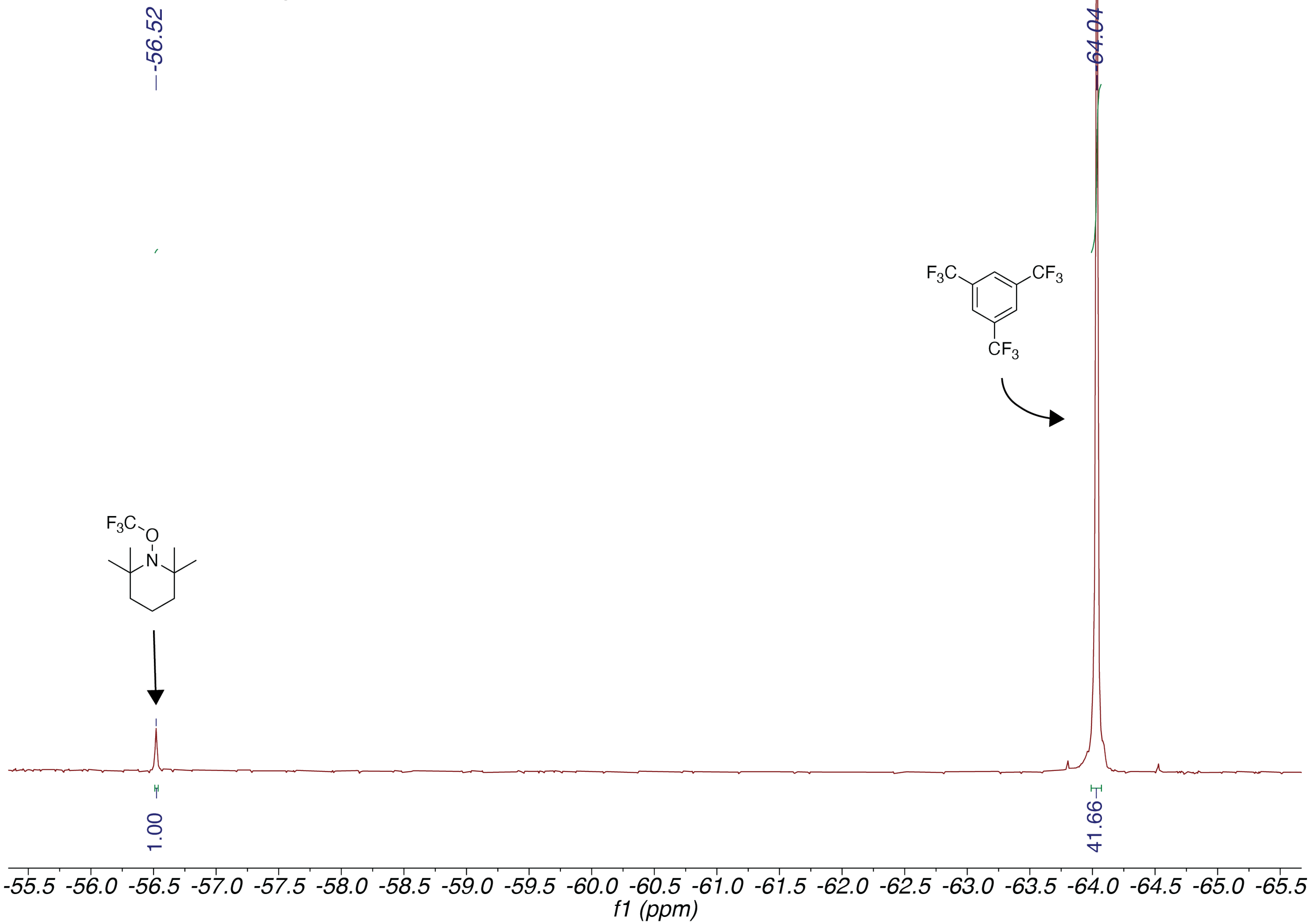

Figure S5. ^{19}F NMR spectrum of a reaction of 0.3 mmol TEMPO•, and 0.03 mmol **II** in 1 mL CH_2Cl_2 acquired following 6 h exposure to a Kessil® KSPR 160L-440 LED lamp at 25 °C. 0.05 mmol of 1,3,5-tris(trifluoromethyl)benzene was used as an internal standard.

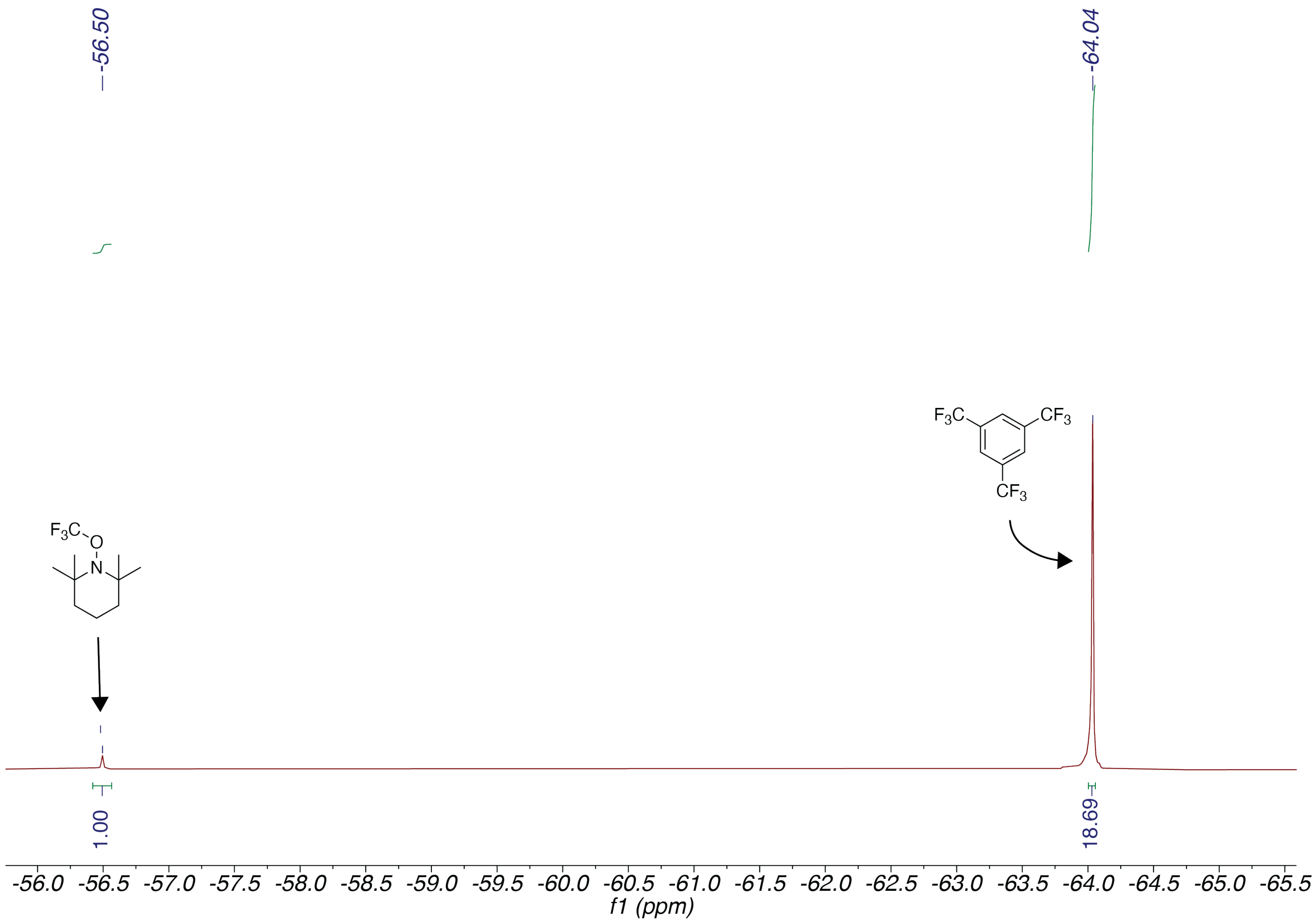

Figure S6. ^{19}F NMR spectrum of a reaction of 0.15 mmol TEMPO•, and 0.015 mmol **II** in 2 mL CH_2Cl_2 acquired following 6 h exposure to a Kessil® KSPR 160L-440 LED lamp at 25 °C. 0.016 mmol of 1,3,5-tris(trifluoromethyl)benzene was used as an internal standard.

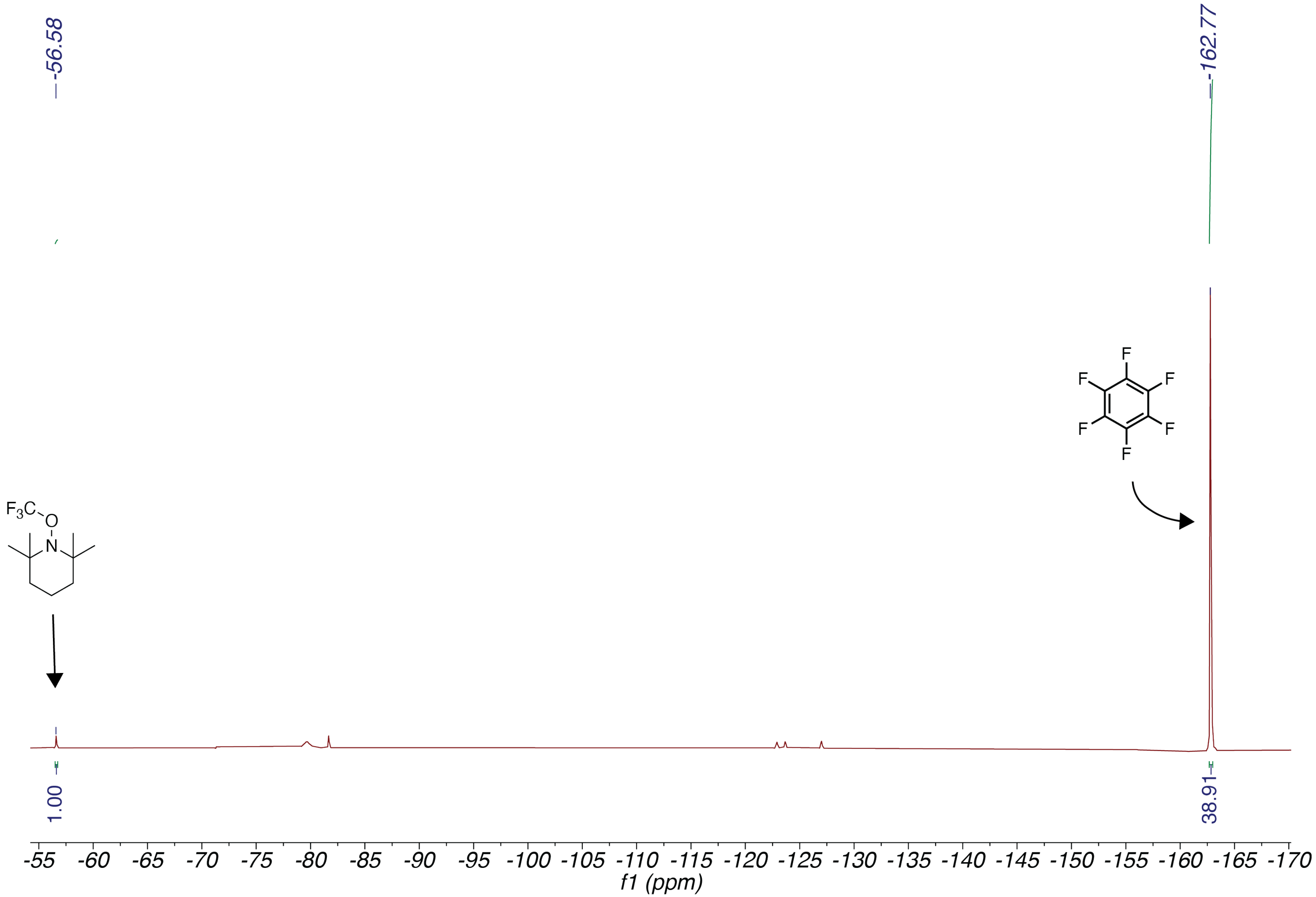

Figure S7. ^{19}F NMR spectrum of a reaction of 0.384 mmol TEMPO•, and 0.024 mmol **II** in 1 mL CH_2Cl_2 acquired following 6 h exposure to a Kessil® KSPR 160L-440 LED lamp at 25 °C. 0.1 mmol hexafluorobenzene was used as an internal standard.

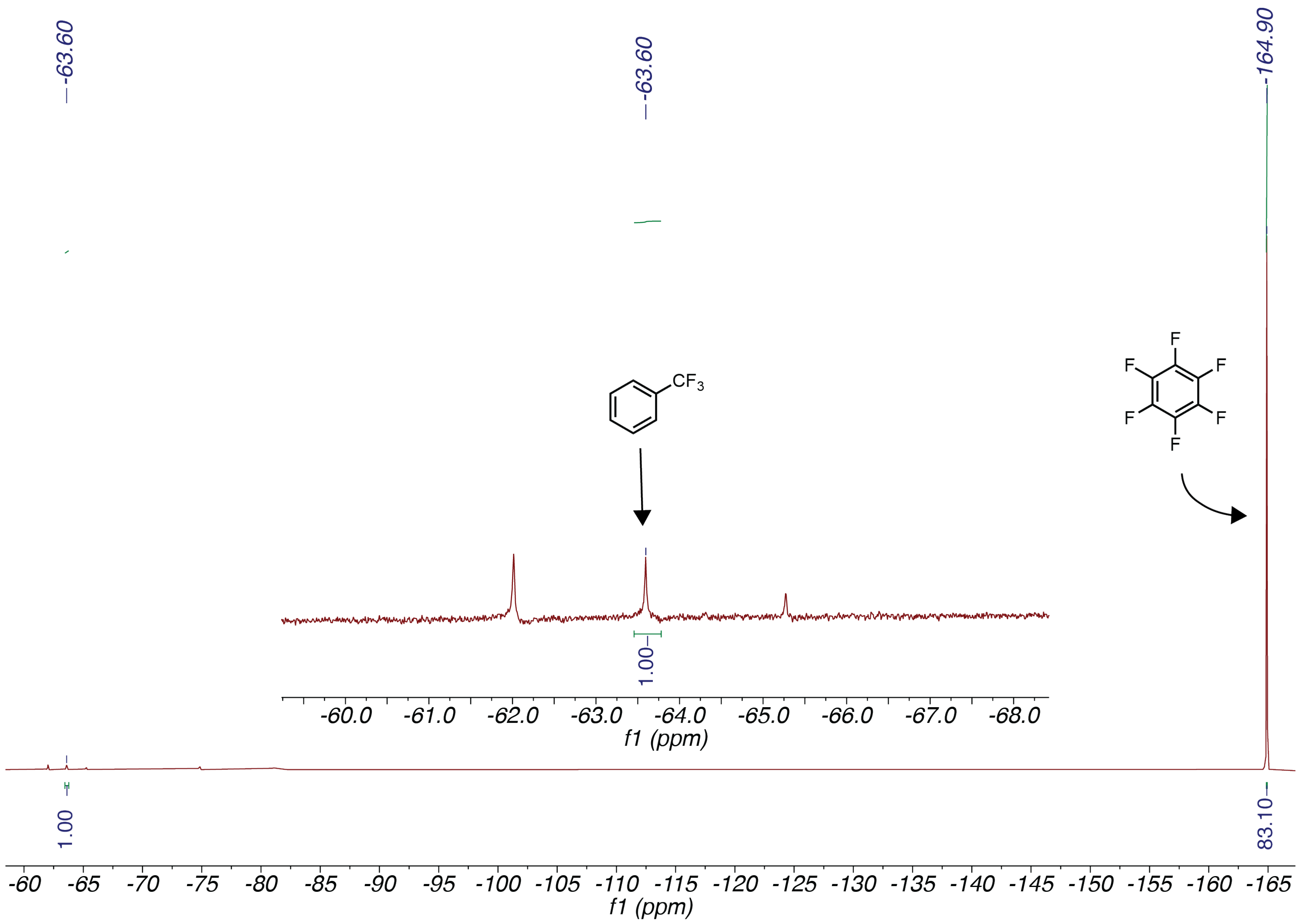

Figure S8. ^{19}F NMR spectrum of a reaction of 0.125 mmol benzene, and 0.025 mmol **II** in 1 mL CH_2Cl_2 acquired following 6 h exposure to a Kessil® KSPR 160L-440 LED lamp at 25 °C. 0.1 mmol of hexafluorobenzene was used as an internal standard.

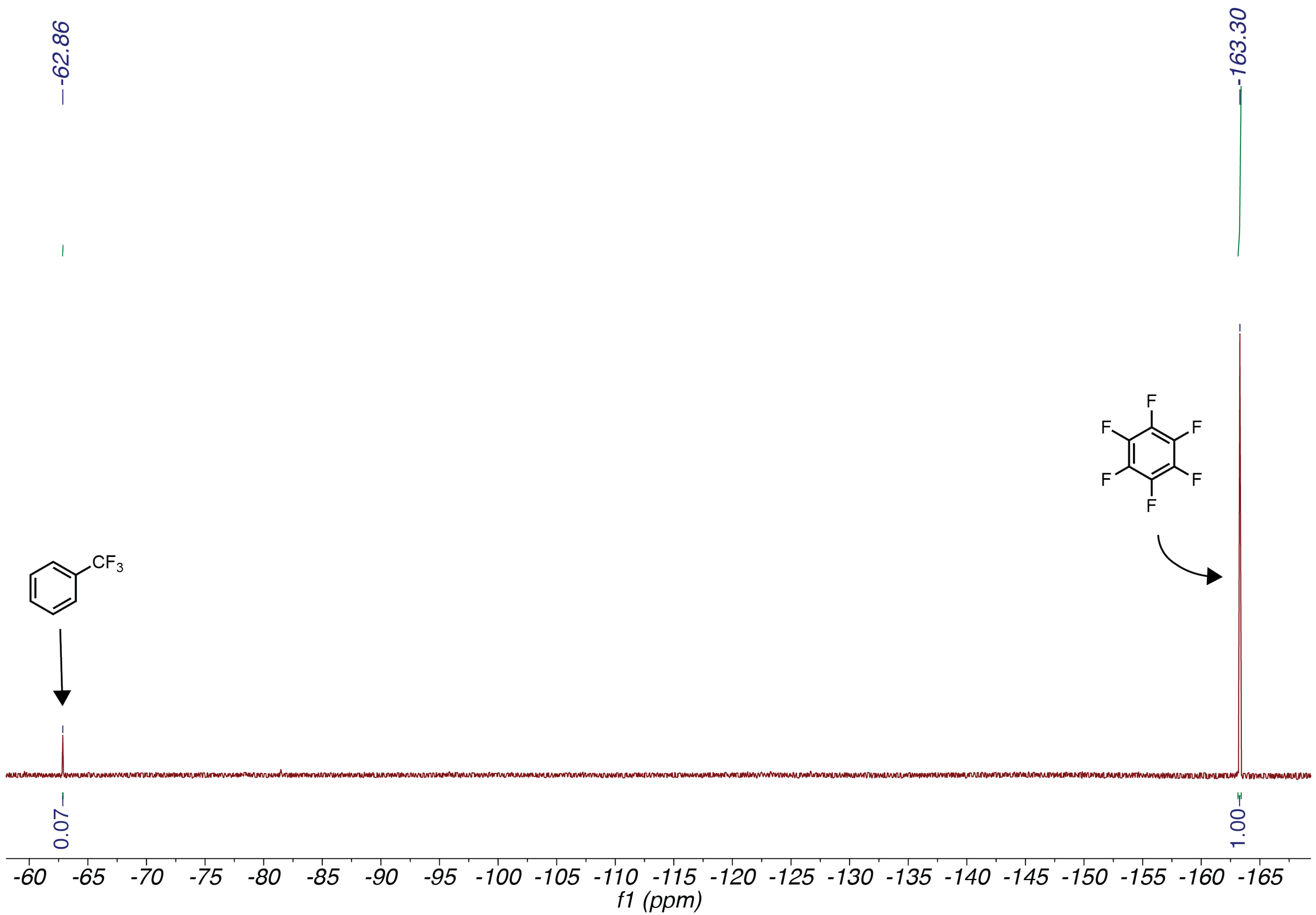

Figure S9. ^{19}F NMR spectrum of a reaction of 0.0126 mmol **II** in 1 mL C_6H_6 acquired following 6 h exposure to a Kessil® KSPR 160L-440 LED lamp at 25 °C. 0.05 mmol of hexafluorobenzene was used as an internal standard.

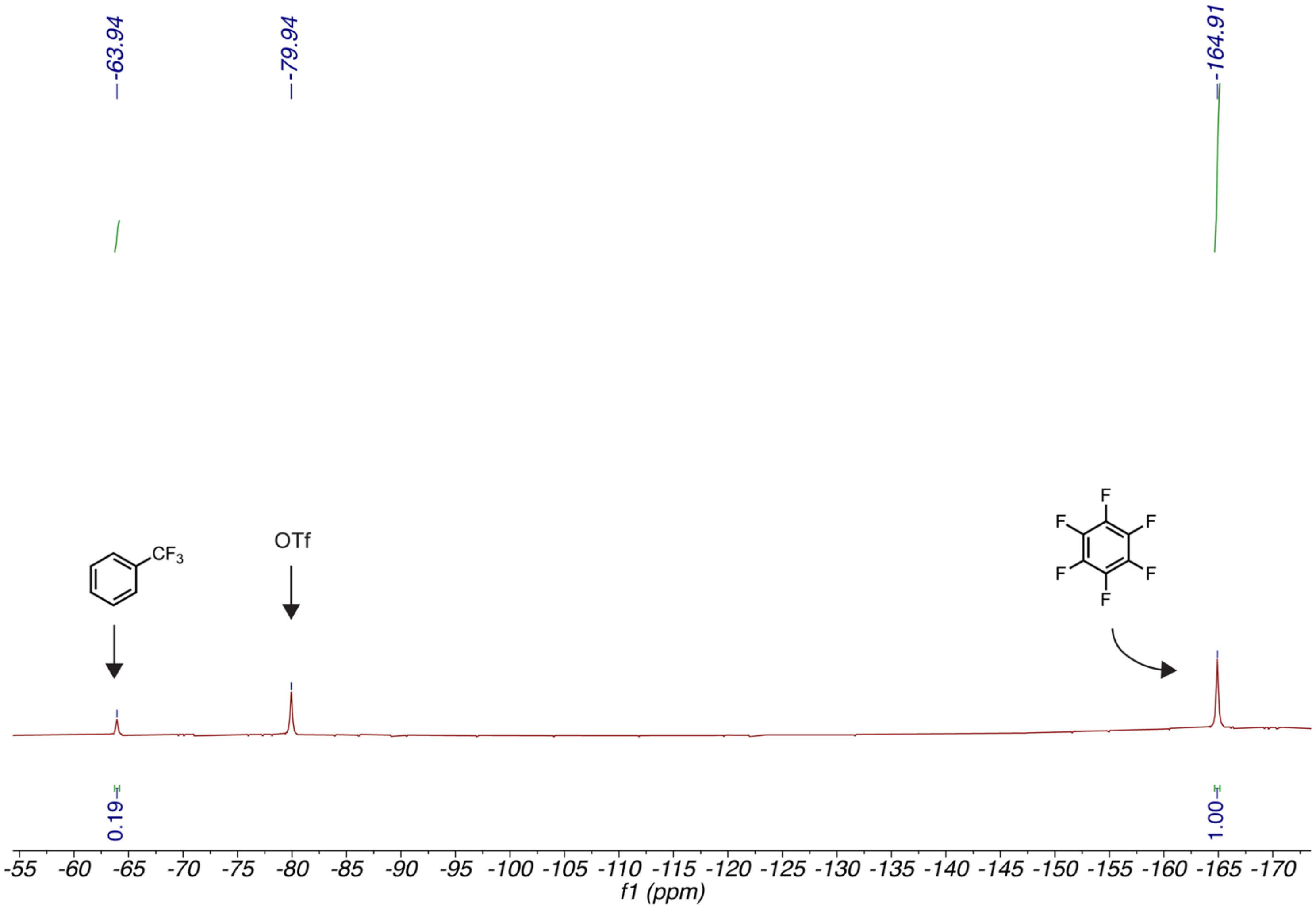

Figure S10. ^{19}F NMR spectrum of a reaction of 0.0125 mmol **II**, and 0.012 mmol *N*,*N*-diisopropylethylamine in 1 mL C_6H_6 acquired following 6 h exposure to a Kessil® KSPR 160L-440 LED lamp at 25 °C. 0.008 mmol of hexafluorobenzene was used as an internal standard.

Catalytic C–H Trifluoromethylation Optimization NMR Data

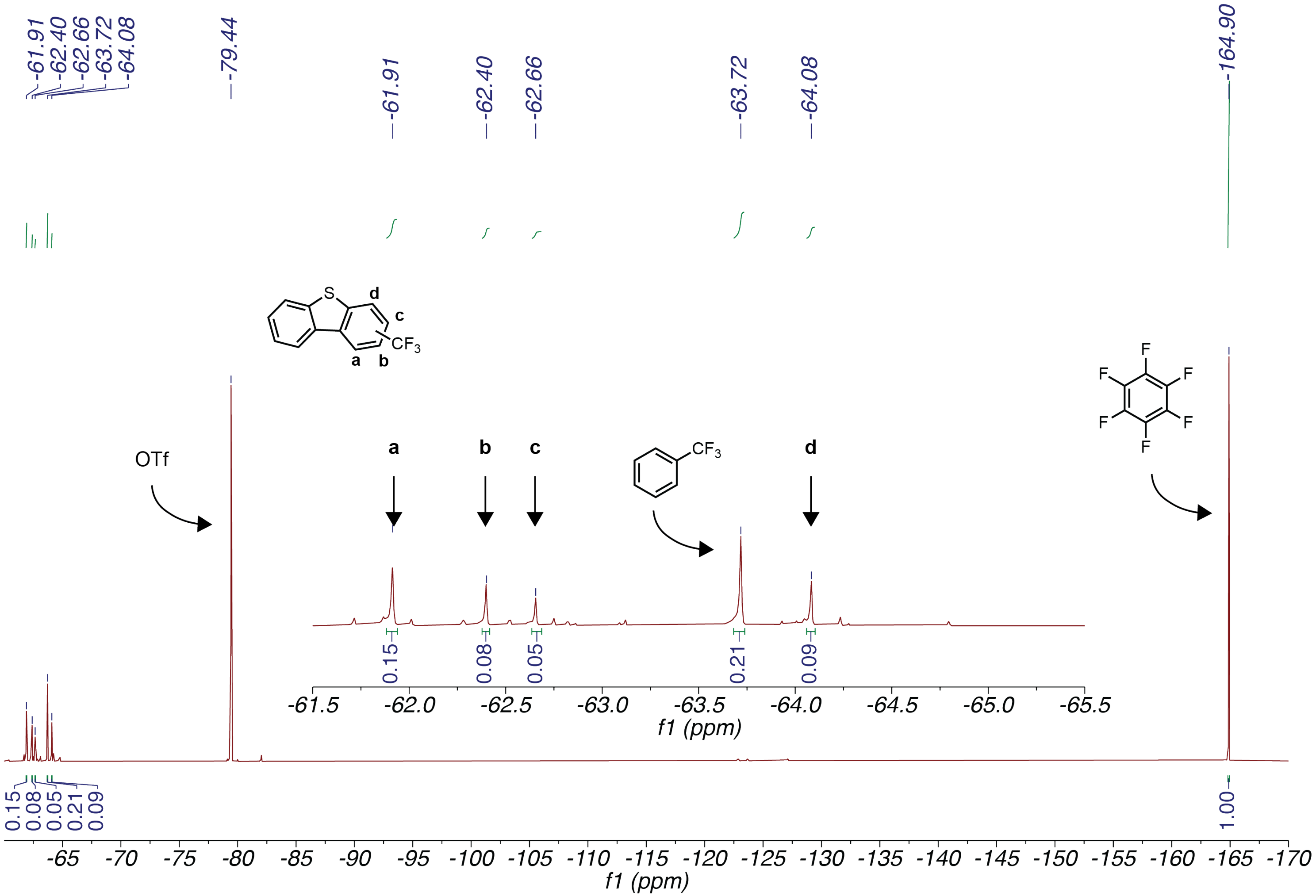

Figure S11. ^{19}F NMR spectrum of a reaction of 0.2 mmol **1** with 0.2 mmol benzene in 1 mL CD_3CN acquired following 6 h exposure to a Kessil® KSPR 160L-440 LED lamp at 25 °C. 0.1 mmol of hexafluorobenzene was used as an internal standard.[1]

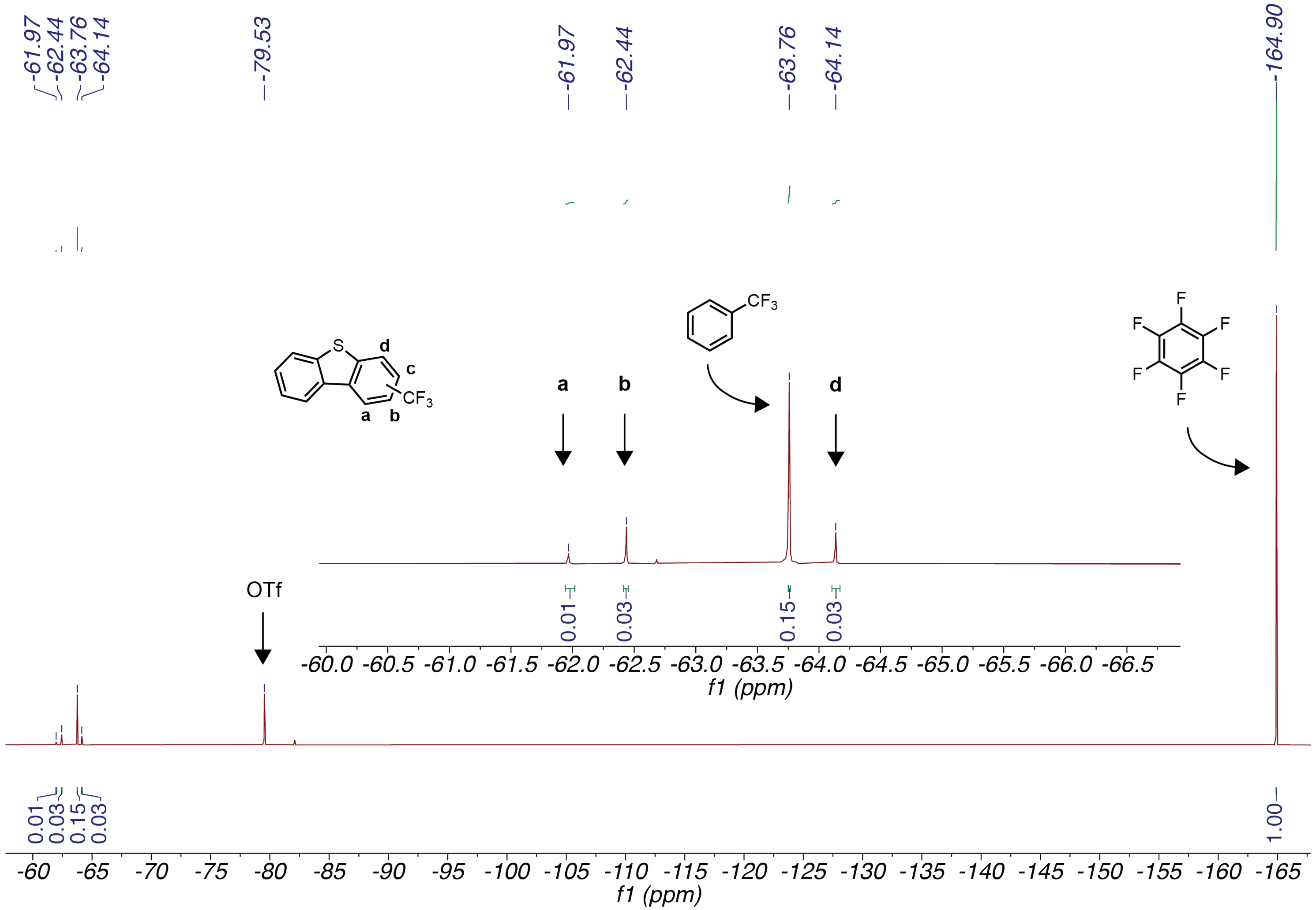

Figure S12. ^{19}F NMR spectrum of a reaction of 0.2 mmol **1** with 2 mmol benzene in 1 mL CD_3CN acquired following 6 h exposure to a Kessil® KSPR 160L-440 LED lamp at 25 °C. 0.05 mmol of hexafluorobenzene was used as an internal standard.[1]

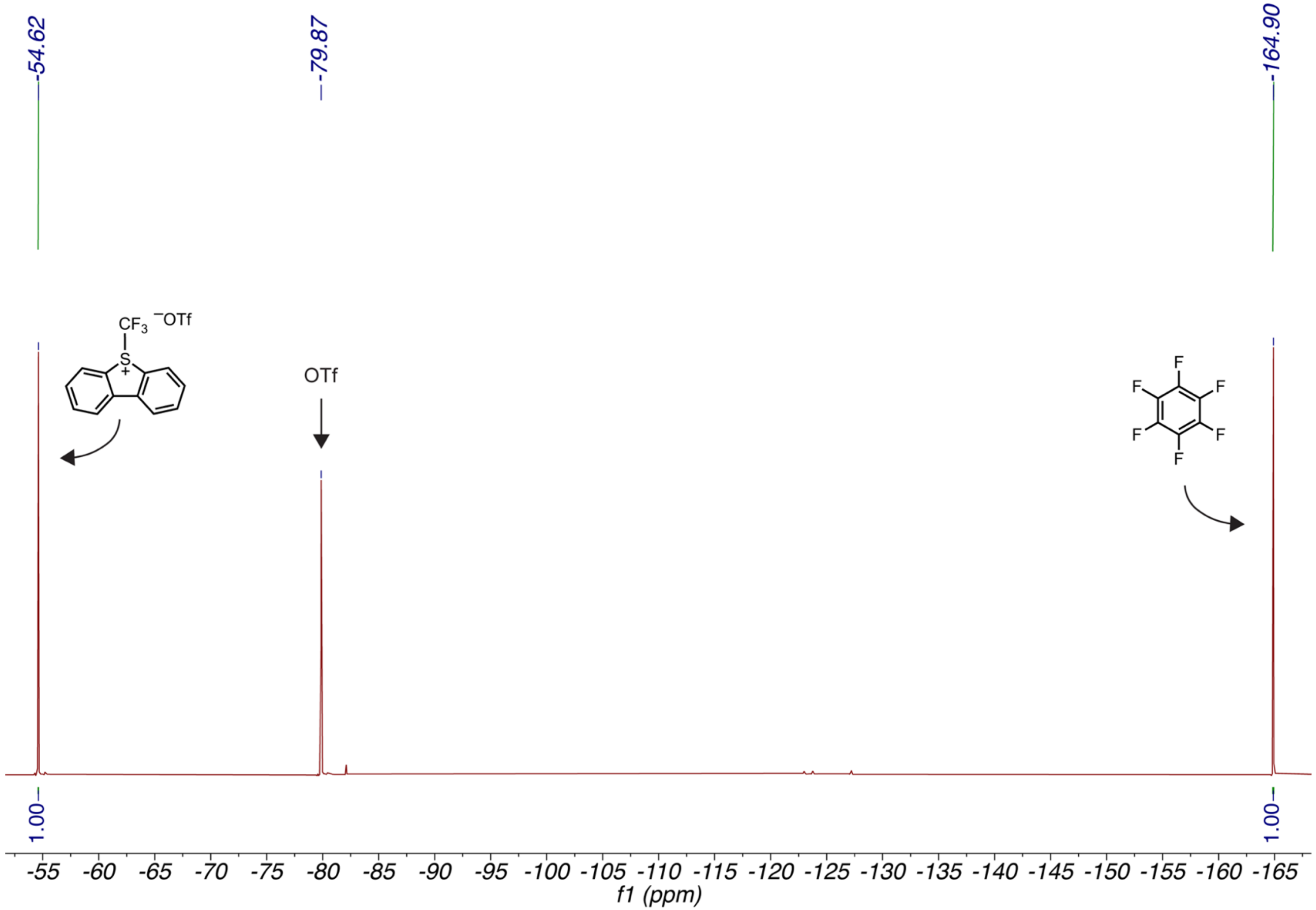

Figure S12. ^{19}F NMR spectrum of a reaction of 0.2 mmol **1** and 2 mmol benzene in 1 mL CD_3CN acquired following 6 h in the dark at 25 °C. 0.1 mmol of hexafluorobenzene was used as an internal standard.

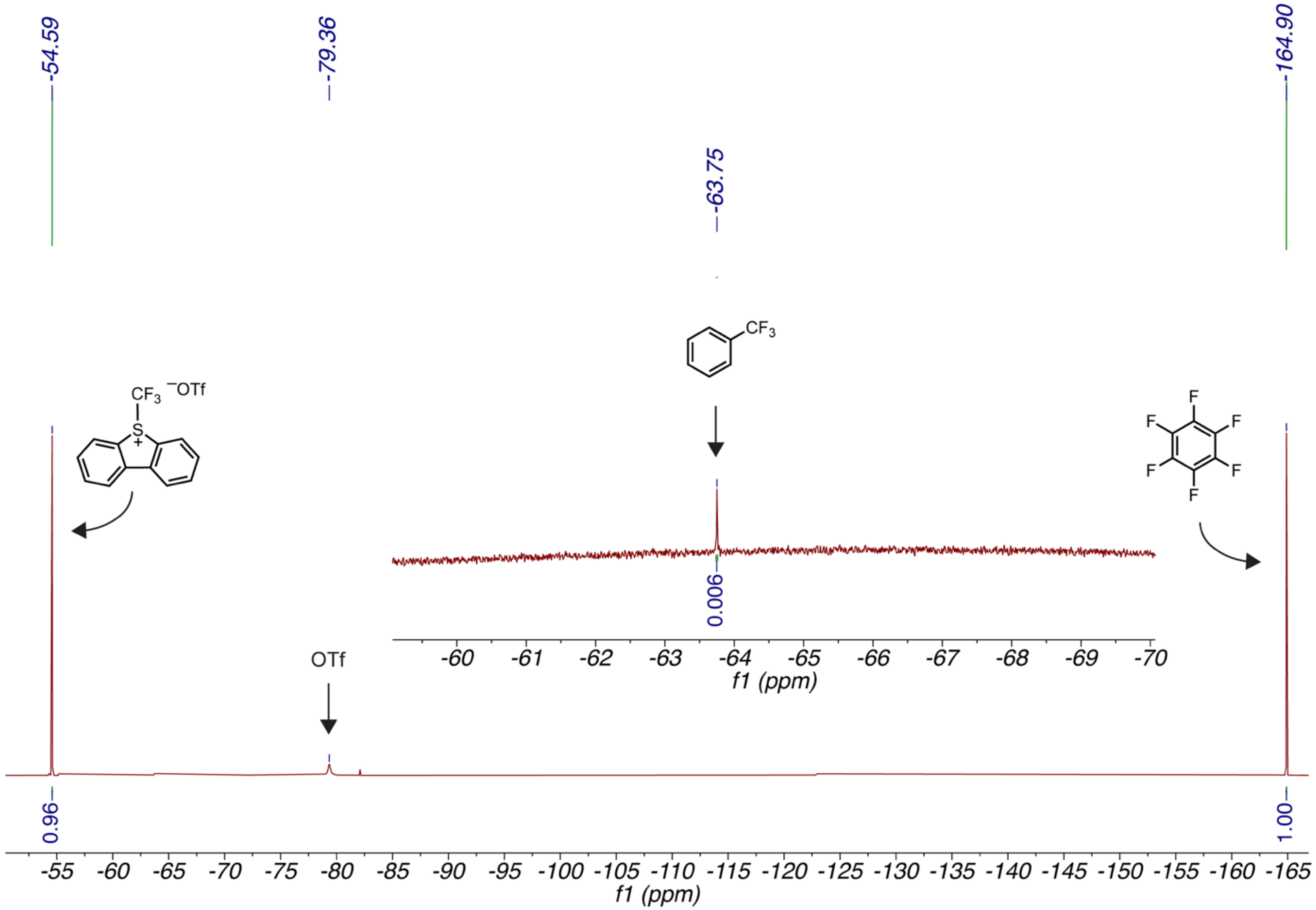

Figure S13. ^{19}F NMR spectrum of a reaction of 0.2 mmol **1** with 2 mmol benzene, and 0.01 mmol **III** in 1 mL CD_3CN acquired following 6 h in the dark at 25 °C. 0.1 mmol of hexafluorobenzene was used as an internal standard.

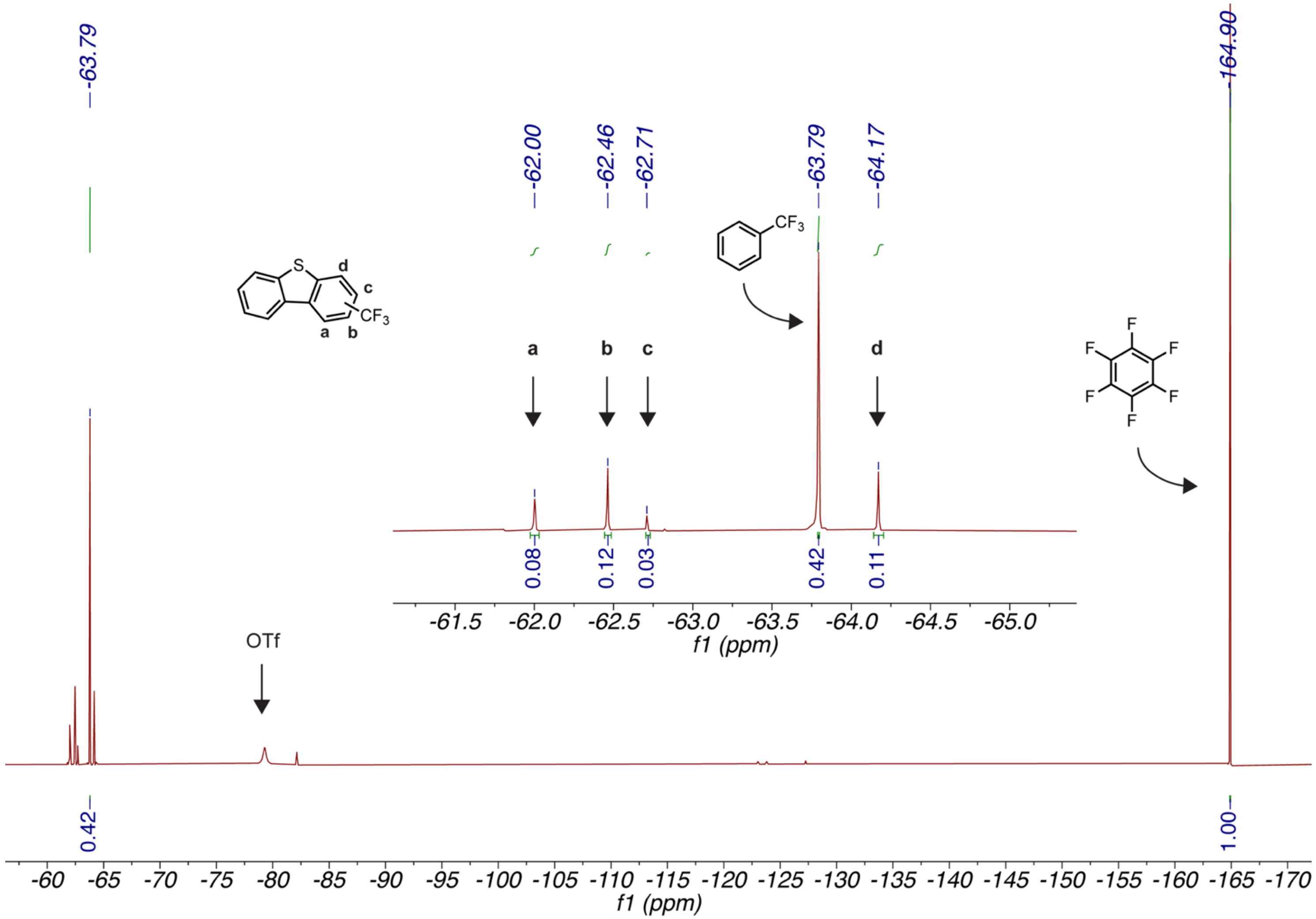

Figure S14. ^{19}F NMR spectrum of a reaction of 0.2 mmol **1** with 2 mmol benzene, and 0.01 mmol $CoCl_2$ in 1 mL CD_3CN acquired following 6 h exposure to a Kessil® KSPR 160L-440 LED lamp at 25 °C. 0.1 mmol of hexafluorobenzene was used as an internal standard.[1]

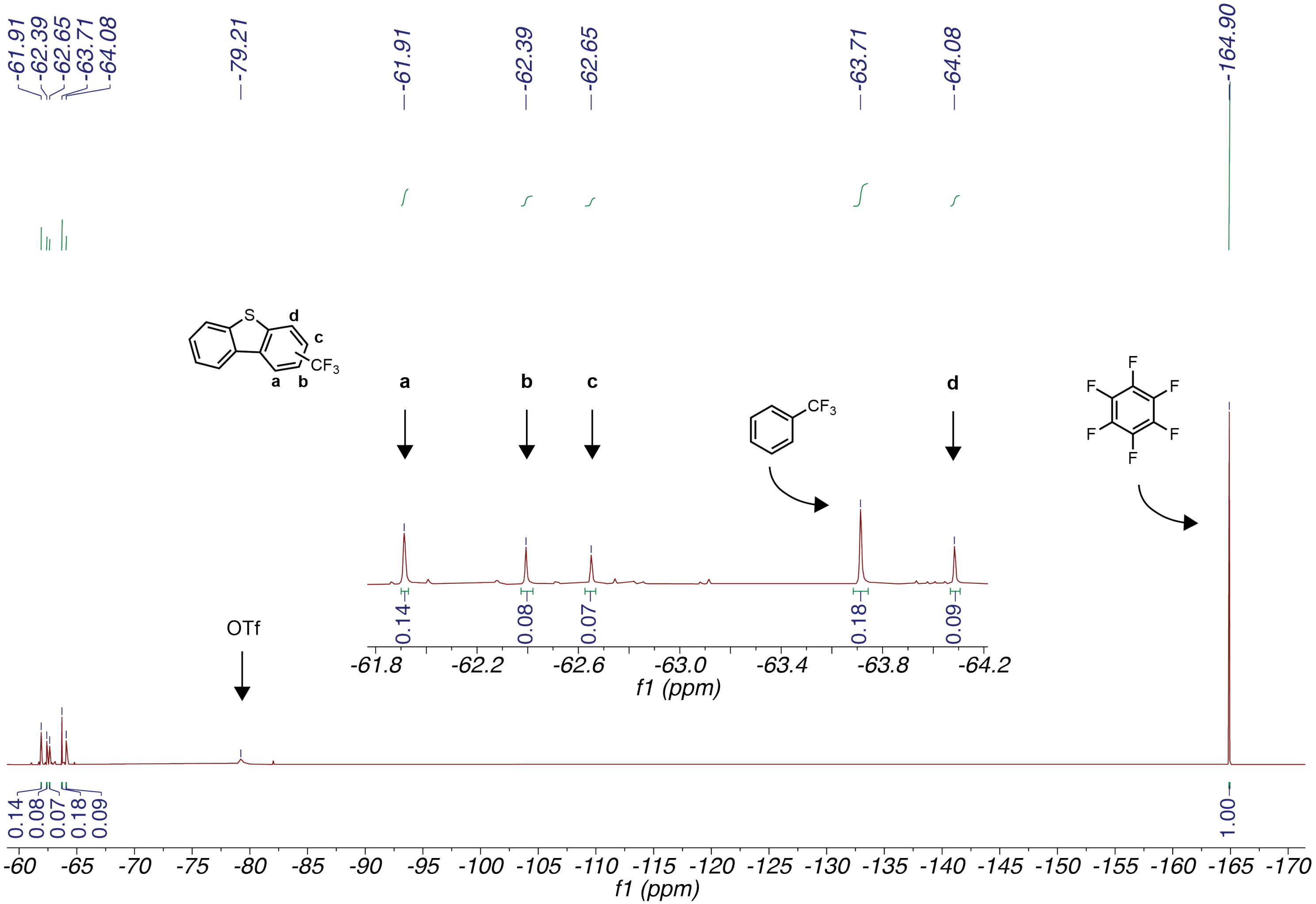

Figure S16. ^{19}F NMR spectrum of a reaction of 0.2 mmol **1** with 0.2 mmol benzene, and 0.01 mmol **III** in 1 mL CD_3CN acquired following 6 h exposure to a Kessil® KSPR 160L-440 LED lamp at 25 °C. 0.1 mmol of hexafluorobenzene was used as an internal standard.[1]

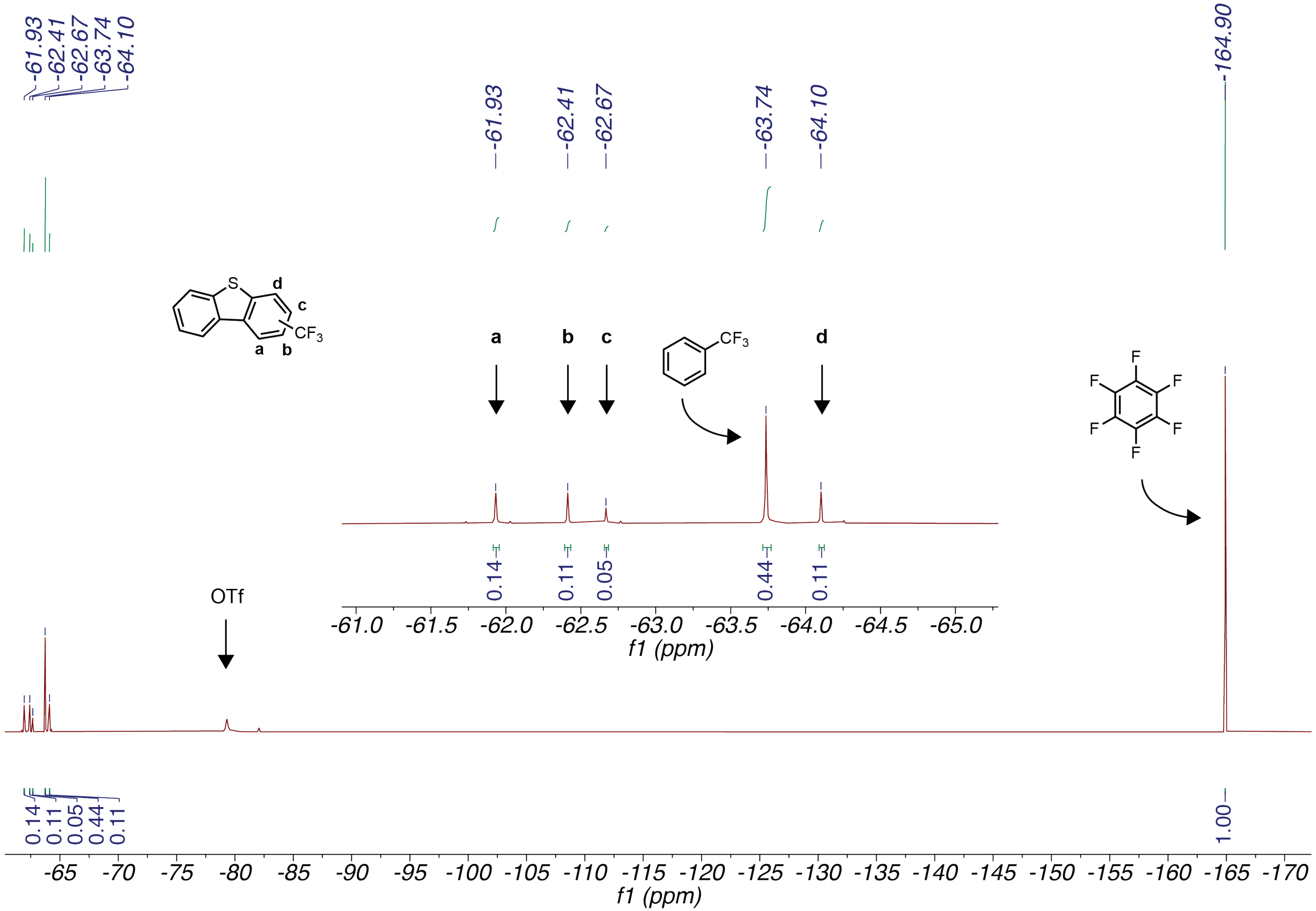

Figure S17. ^{19}F NMR spectrum of a reaction of 0.2 mmol **1** with 0.6 mmol benzene, and 0.01 mmol **III** in 1 mL CD_3CN acquired following 6 h exposure to a Kessil® KSPR 160L-440 LED lamp at 25 °C. 0.1 mmol of hexafluorobenzene was used as an internal standard.[1]

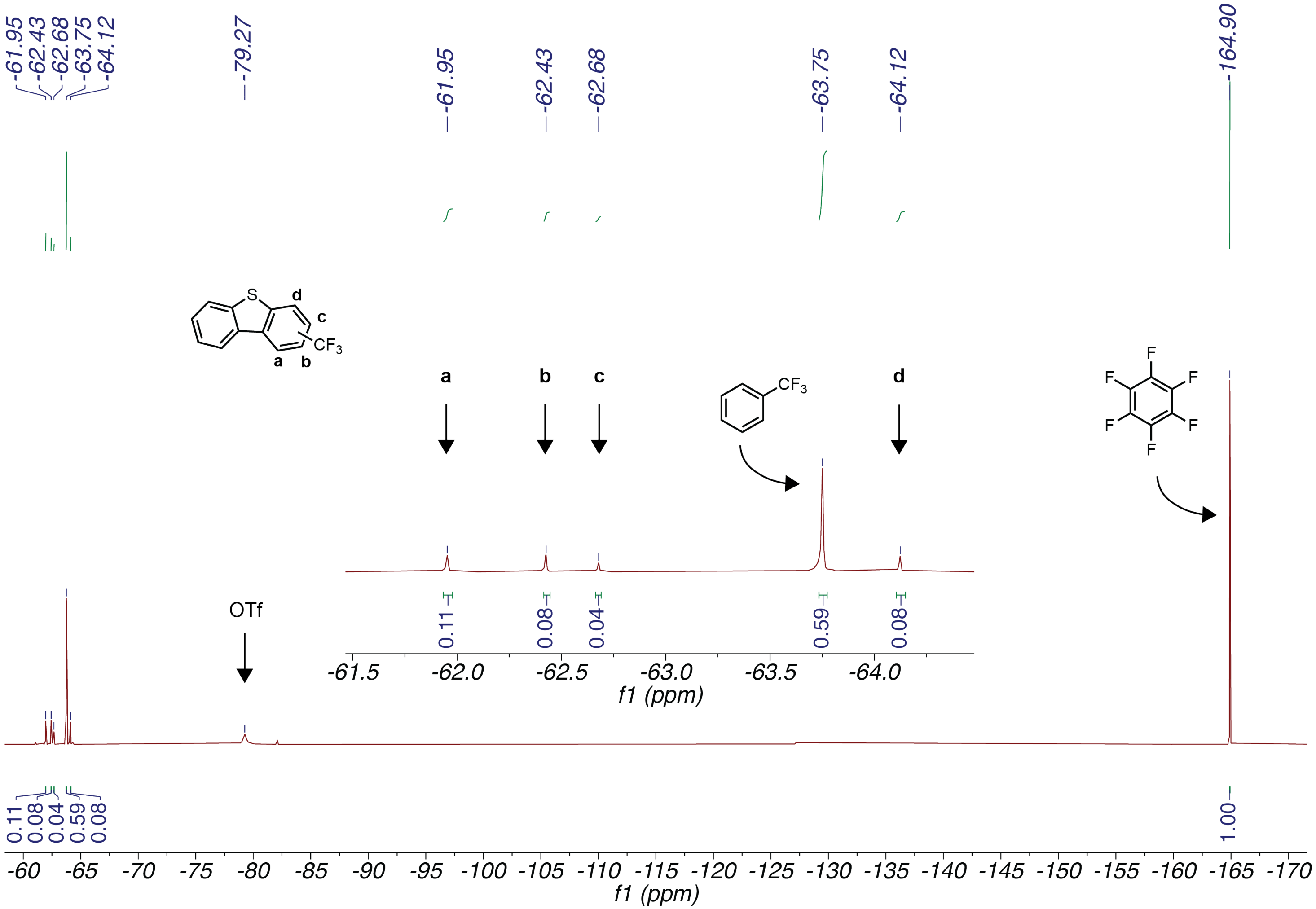

Figure S18. ^{19}F NMR spectrum of a reaction of 0.2 mmol **1** with 1 mmol benzene, and 0.01 mmol **III** in 1 mL CD_3CN acquired following 6 h exposure to a Kessil® KSPR 160L-440 LED lamp at 25 °C. 0.1 mmol of hexafluorobenzene was used as an internal standard.[1]

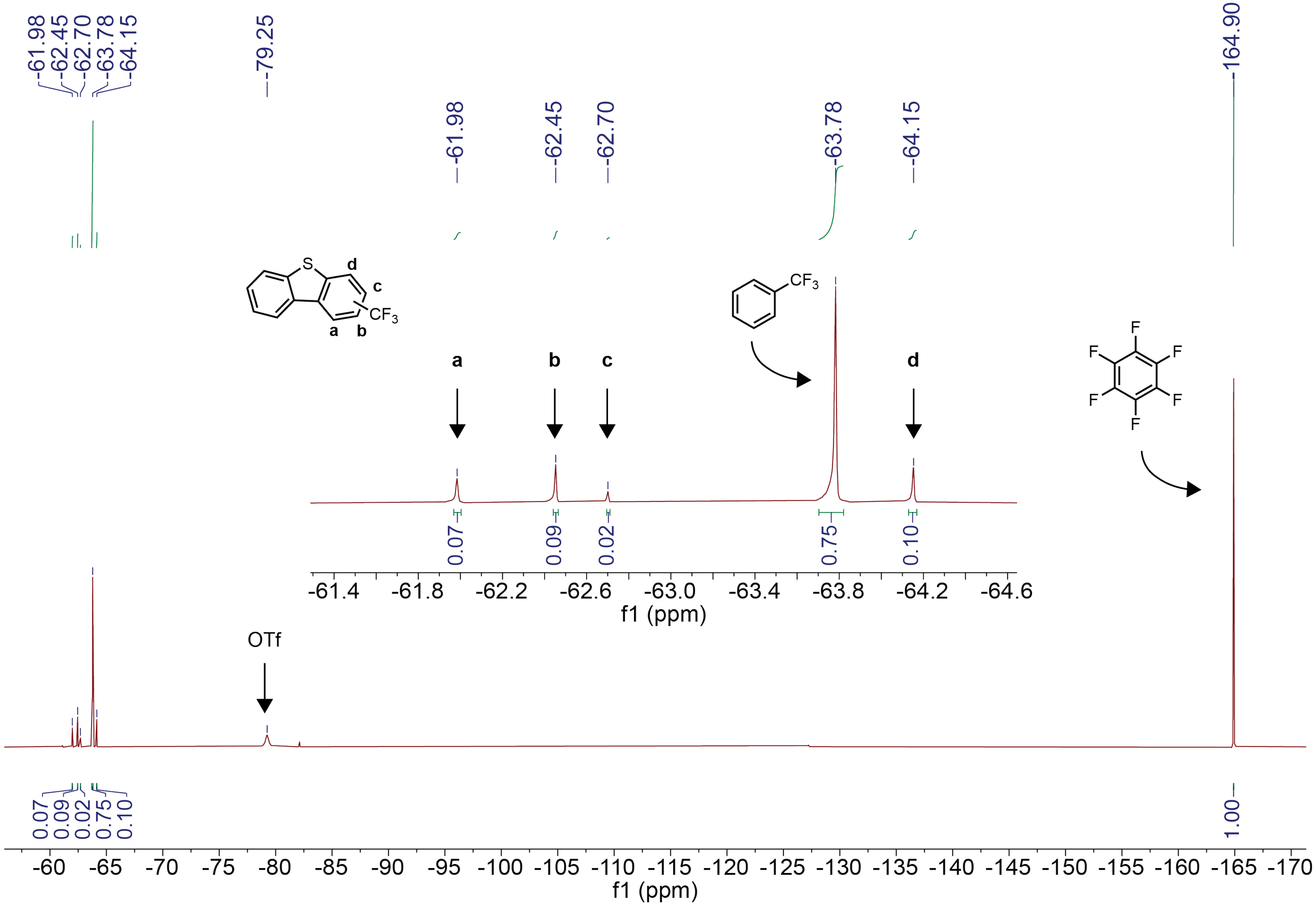

Figure S19. ^{19}F NMR spectrum of a reaction of 0.2 mmol **1** with 2 mmol benzene, and 0.01 mmol **III** in 1 mL CD_3CN acquired following 6 h exposure to a Kessil® KSPR 160L-440 LED lamp at 25 °C. 0.1 mmol of hexafluorobenzene was used as an internal standard.[1]

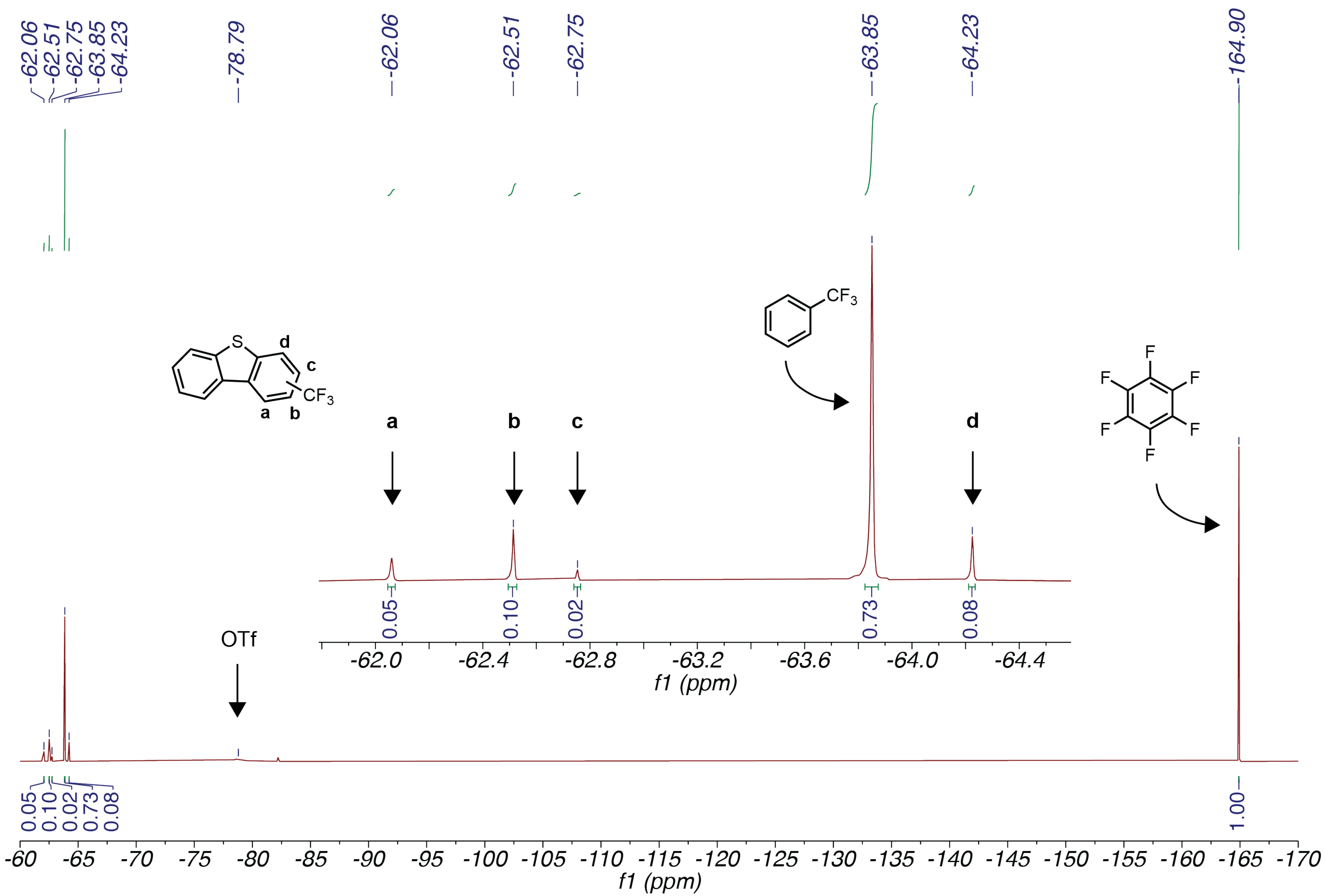

Figure S20. ^{19}F NMR spectrum of a reaction of 0.2 mmol **1** with 4 mmol benzene, and 0.01 mmol **III** in 1 mL CD_3CN acquired following 6 h exposure to a Kessil® KSPR 160L-440 LED lamp at 25 °C. 0.1 mmol of hexafluorobenzene was used as an internal standard.[1]

Catalytic C–H Trifluoromethylation Substrate Scope NMR Data

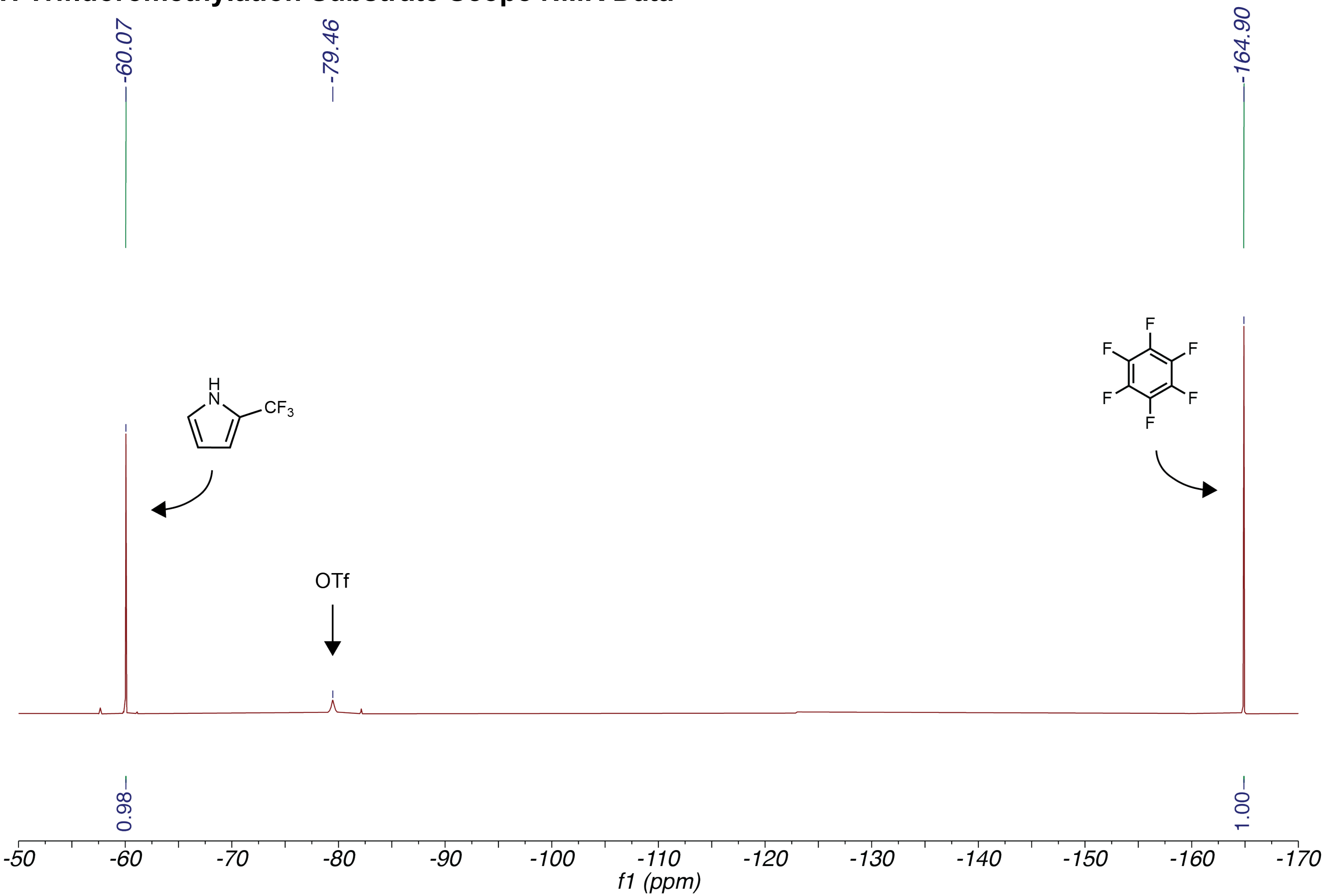

Figure S21. ^{19}F NMR spectrum of a reaction of 0.2 mmol **1** with 2 mmol pyrrole, and 0.01 mmol **III** in 1 mL CD_3CN acquired following 6 h exposure to a Kessil® KSPR 160L-440 LED lamp at 25 °C. 0.1 mmol of hexafluorobenzene was used as an internal standard.[1]

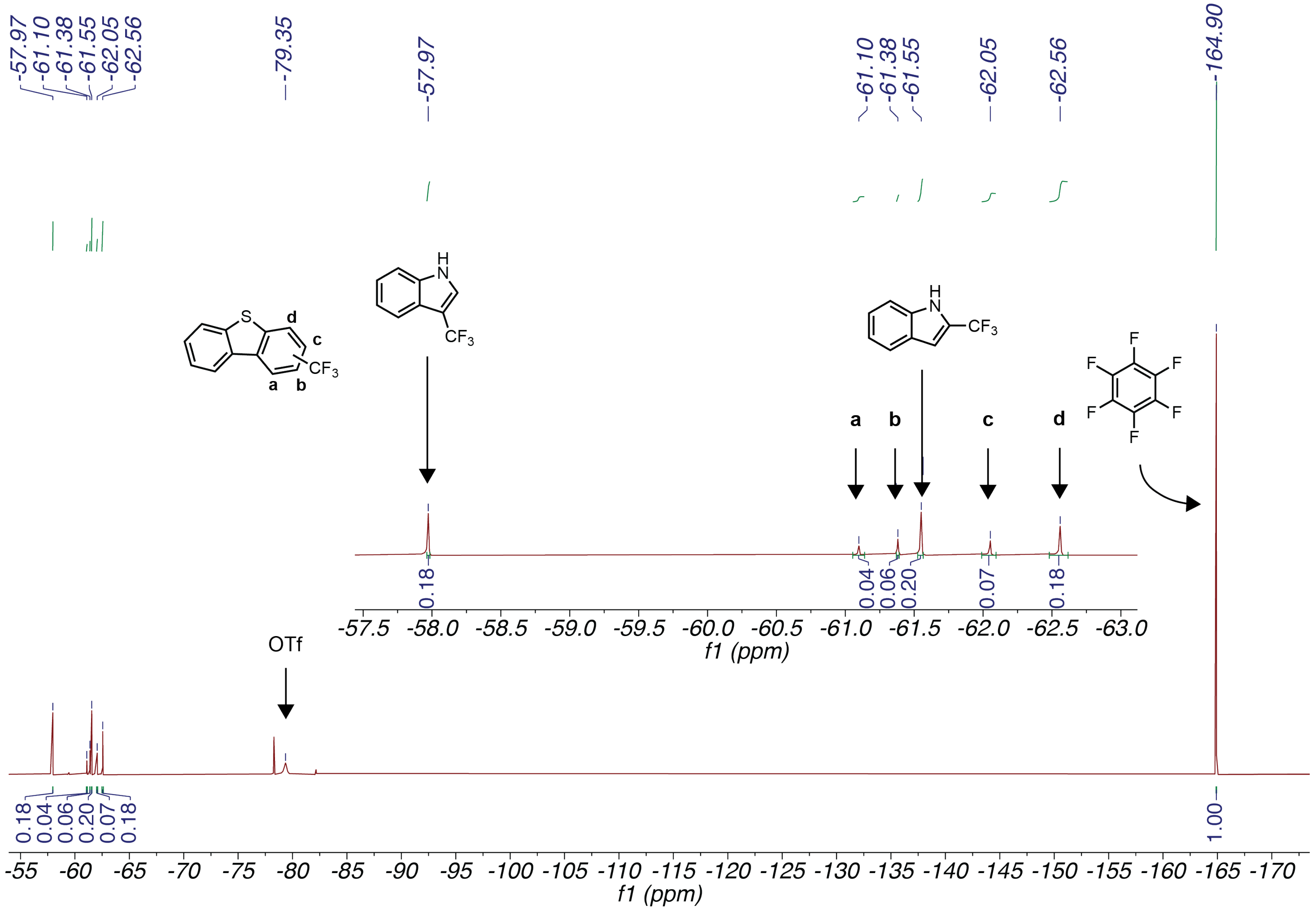

Figure S22. ^{19}F NMR spectrum of a reaction of 0.2 mmol **1** with 2 mmol indole, and 0.01 mmol **III** in 1 mL CD_3CN acquired following 6 h exposure to a Kessil® KSPR 160L-440 LED lamp at 25 °C. 0.1 mmol of hexafluorobenzene was used as an internal standard.[1]

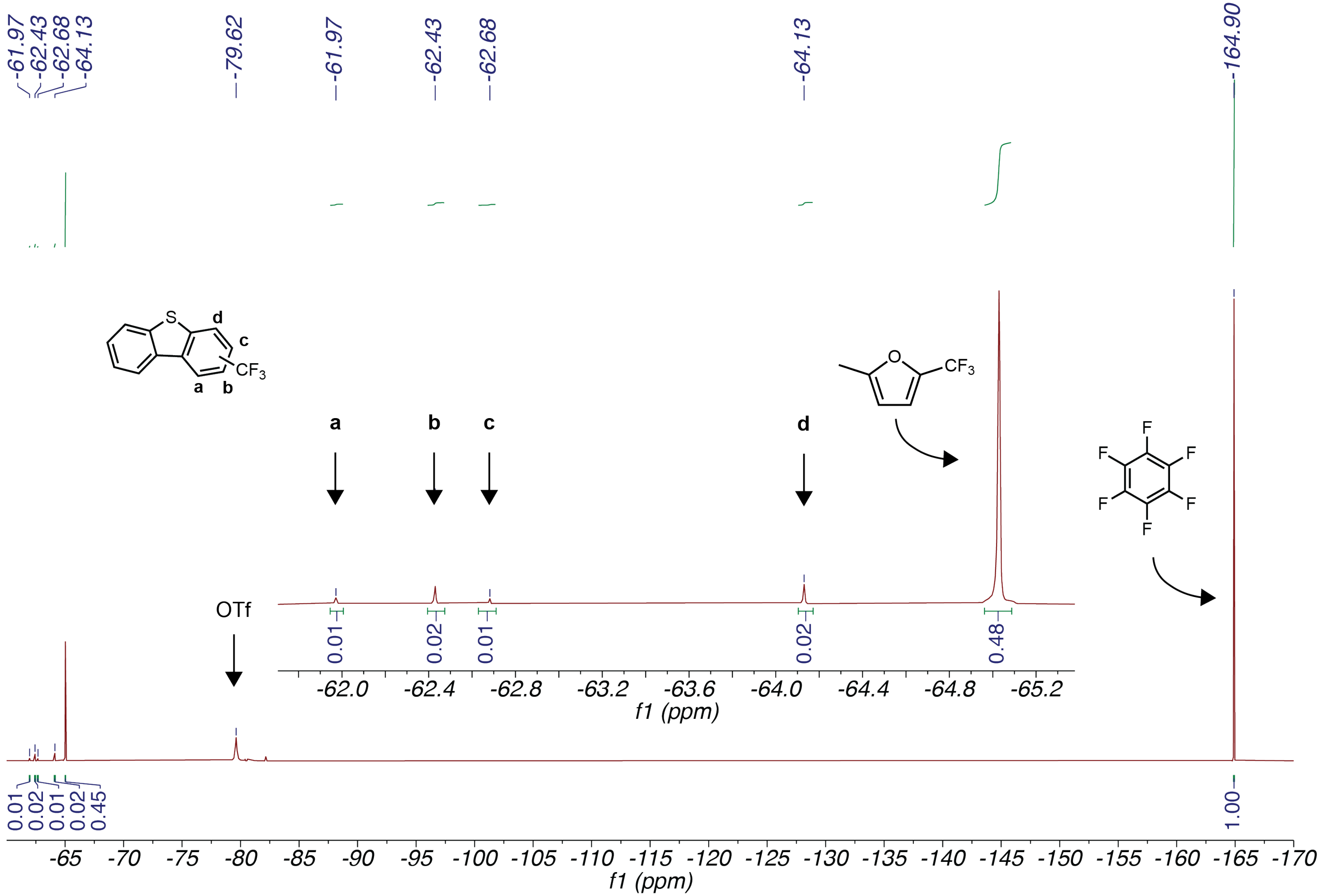

Figure S23. ^{19}F NMR spectrum of a reaction of 0.2 mmol **1** with 2 mmol 2-methylfuran, and 0.01 mmol **III** in 1 mL CD_3CN acquired following 6 h exposure to a Kessil® KSPR 160L-440 LED lamp at 25 °C. 0.1 mmol of hexafluorobenzene was used as an internal standard.[1]

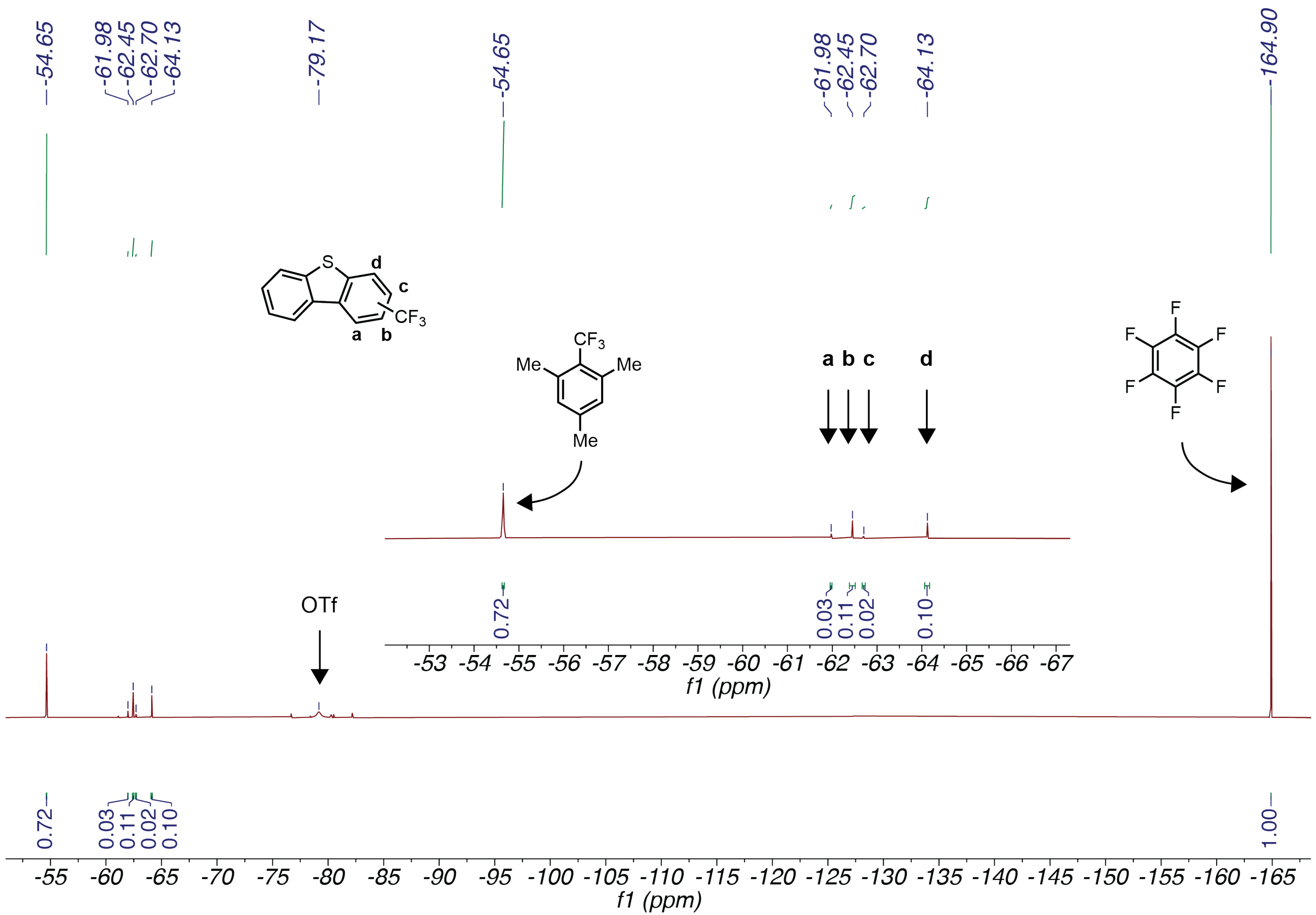

Figure S24. ^{19}F NMR spectrum of a reaction of 0.2 mmol **1** with 2 mmol mesitylene, and 0.01 mmol **III** in 1 mL CD_3CN acquired following 6 h exposure to a Kessil® KSPR 160L-440 LED lamp at 25 °C. 0.1 mmol of hexafluorobenzene was used as an internal standard.[1]

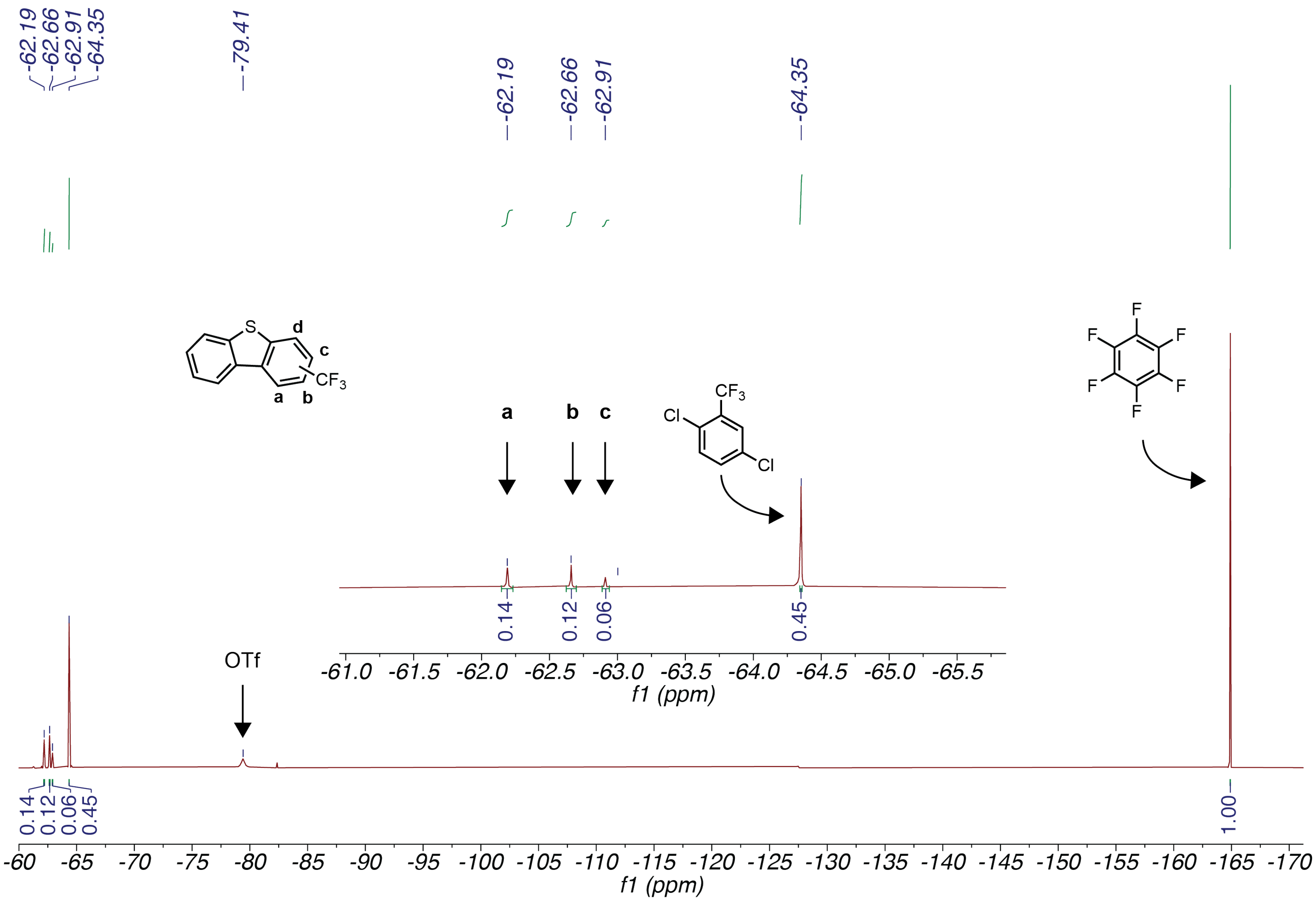

Figure S25. ^{19}F NMR spectrum of a reaction of 0.2 mmol **1** with 2 mmol 1,4-dichlorobenzene, and 0.01 mmol **III** in 1 mL CD_3CN acquired following 6 h exposure to a Kessil® KSPR 160L-440 LED lamp at 25 °C. 0.1 mmol of hexafluorobenzene was used as an internal standard.[1]

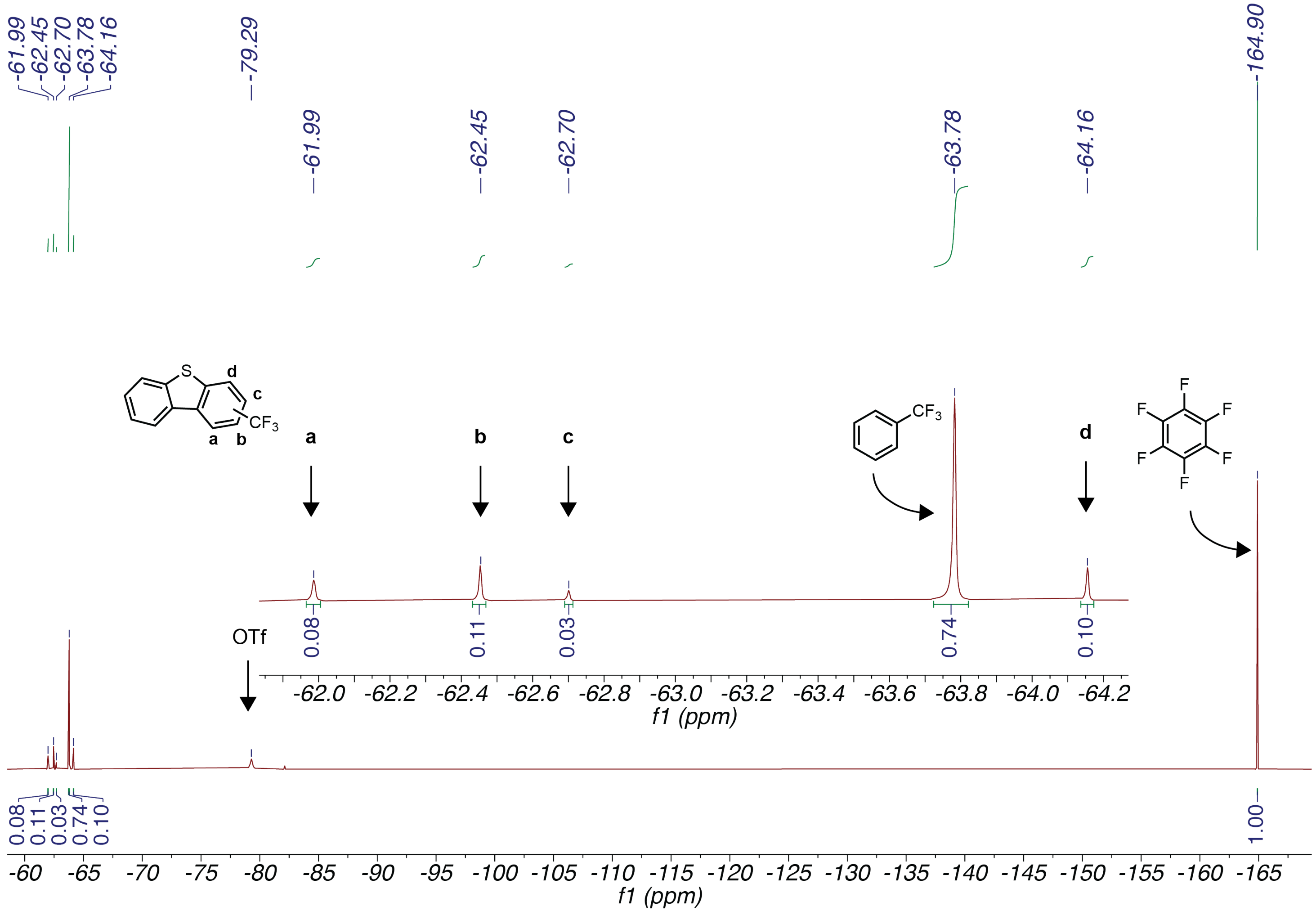

Figure S26. ^{19}F NMR spectrum of a reaction of 0.2 mmol **1** with 2 mmol benzene, and 0.01 mmol **II** in 1 mL CD_3CN acquired following 6 h exposure to a Kessil® KSPR 160L-440 LED lamp at 25 °C. 0.1 mmol of hexafluorobenzene was used as an internal standard.[1]

ESI-MS Data

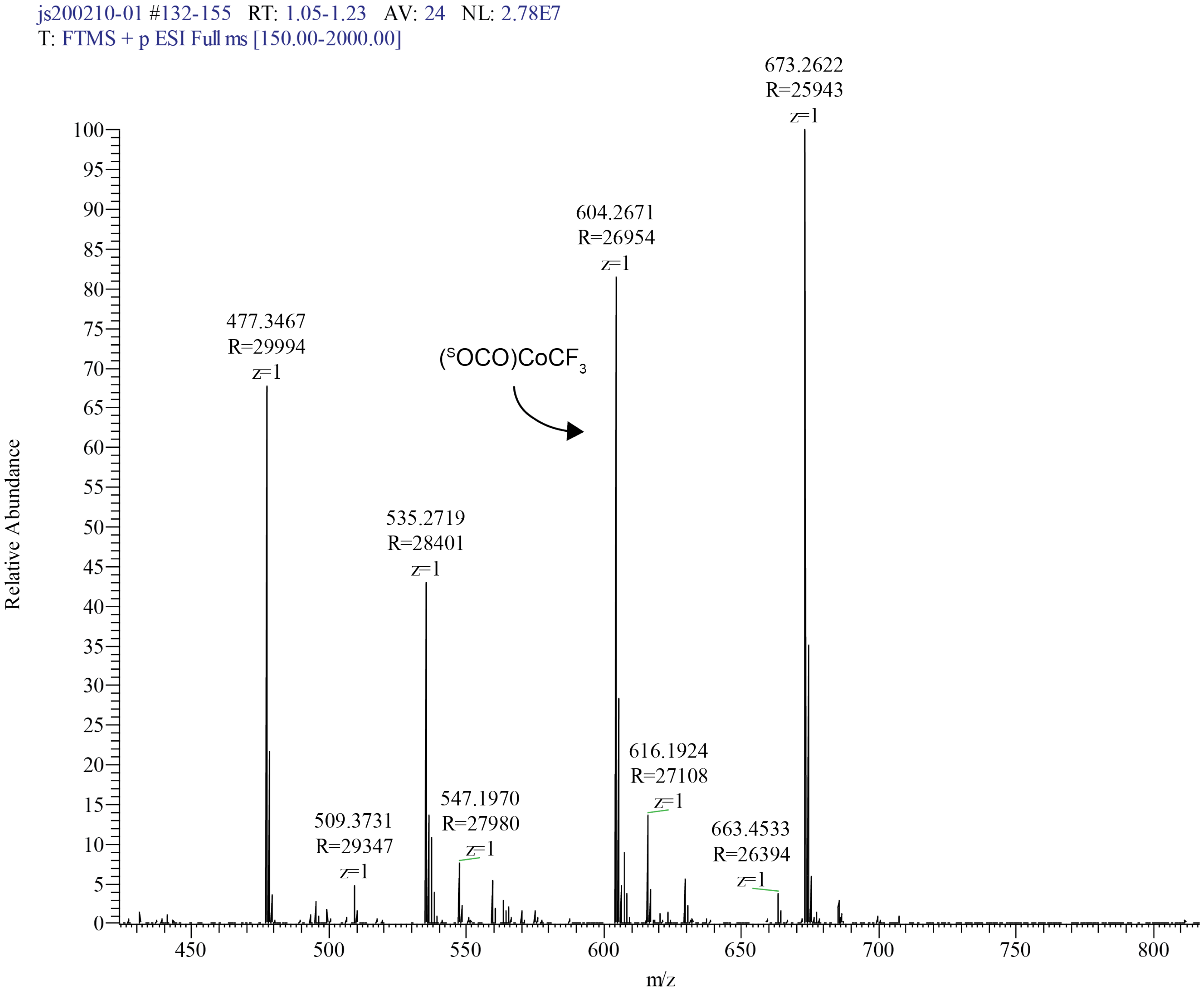

Figure S27. HR-ESI-MS of isolated **II**

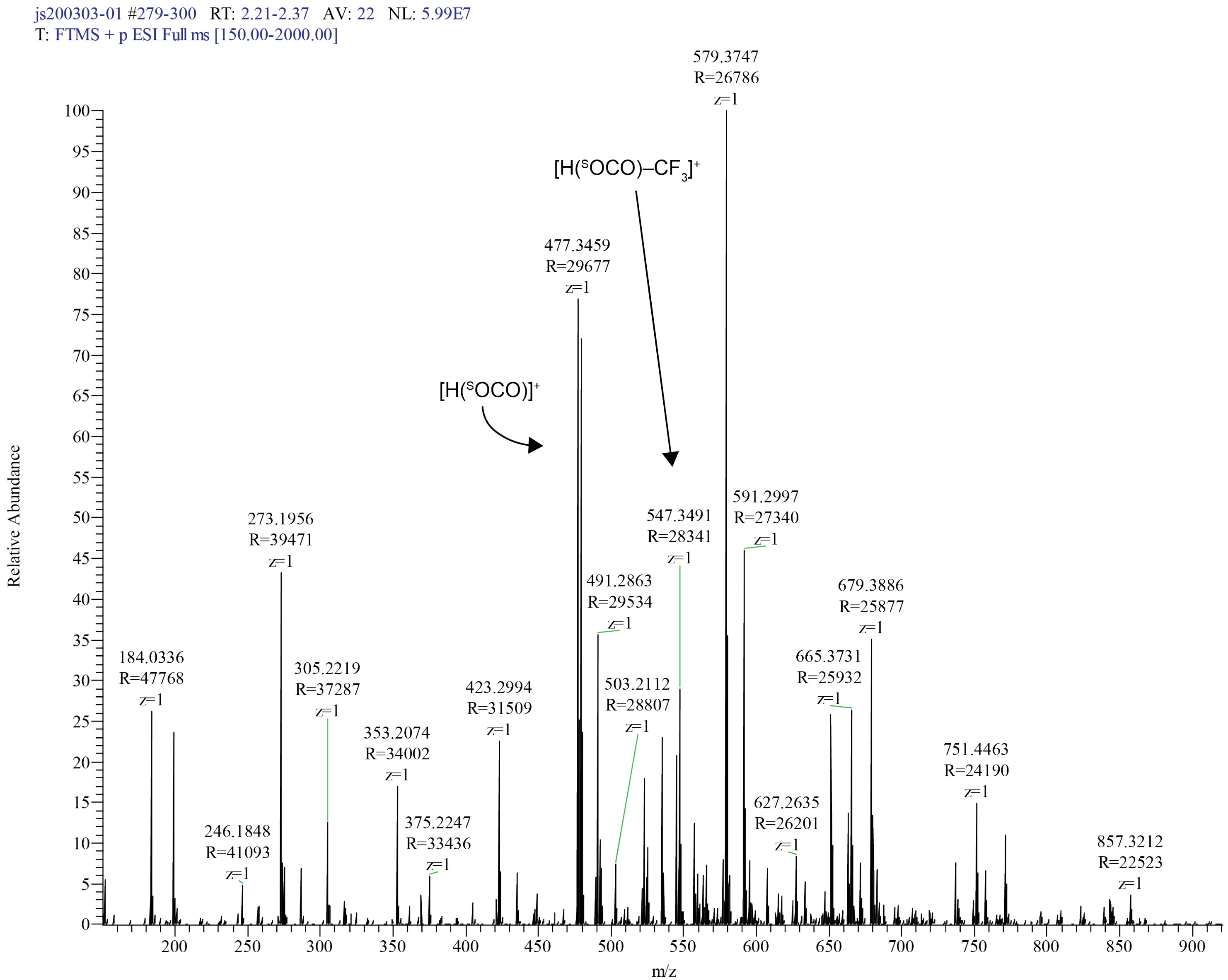

Figure S28. HR-ESI-MS spectrum of the reaction of 0.3 mmol TEMPO•, and 0.03 mmol **II** in 1 mL CH_2Cl_2 acquired following 6 h exposure to a Kessil® KSPR 160L-440 LED lamp at 25 °C.

X-Ray Crystallographic Data

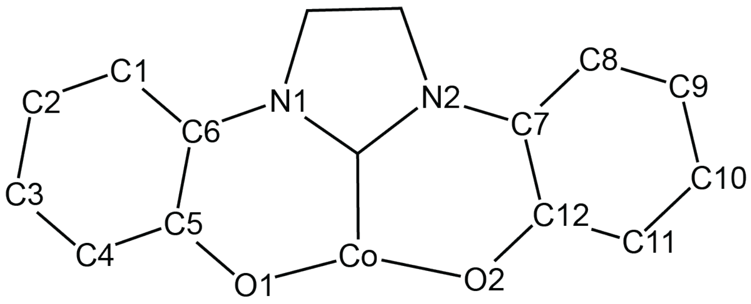

Bond	(^{S}OCO)$^{2-}$Co^{II}(MeCN)[a]	[(^{S}OCO)^{0}Co^{II}(THF)$_3$](PF$_6$)$_2$[b]	Average	(^{S}OCO)$^{-}$Co^{III}CF$_3$(MeCN)OTf	|Difference|
C1–C2	1.380(4)	1.371(3)	1.375	1.373(2)	0.002
C2–C3	1.404(4)	1.424(3)	1.414	1.415(2)	0.001
C3–C4	1.388(4)	1.359(3)	1.374	1.374(2)	0.000
C4–C5	1.422(4)	1.448(3)	1.435	1.444(2)	0.009
C5–C6	1.407(4)	1.446(3)	1.427	1.427(2)	0.000
C6–C1	1.397(4)	1.399(3)	1.398	1.408(2)	0.01
C7–C8	1.393(4)	1.407(3)	1.400	1.407(2)	0.007
C8–C9	1.387(4)	1.366(3)	1.377	1.380(2)	0.003
C9–C10	1.395(4)	1.423(3)	1.409	1.407(2)	0.002
C10–C11	1.397(4)	1.373(3)	1.385	1.386(2)	0.001
C11–C12	1.414(4)	1.434(3)	1.424	1.439(2)	0.015
C12–C7	1.413(4)	1.431(3)	1.422	1.420(2)	0.002
C5–O1	1.337(4)	1.287(3)	1.312	1.304(2)	0.008
C12–O2	1.339(4)	1.301(3)	1.320	1.312(2)	0.008
C6–N1	1.409(4)	1.376(3)	1.392	1.390(2)	0.002
C7–N2	1.406(4)	1.383(3)	1.395	1.397(2)	0.002

Figure S29. Comparison of the ligand metrical data of the OCO ligand for **II** vs. average OCO ligand bond length of the fully reduced form **III** and the fully oxidized form [(OCO^{0})Co^{II}(THF)$_3$](PF$_6$)$_2$.[a,b] Bond lengths for **III** and [(OCO^{0})Co^{II}(THF)$_3$](PF$_6$)$_2$ were taken from reference 2. Bond lengths are reported in angstroms.

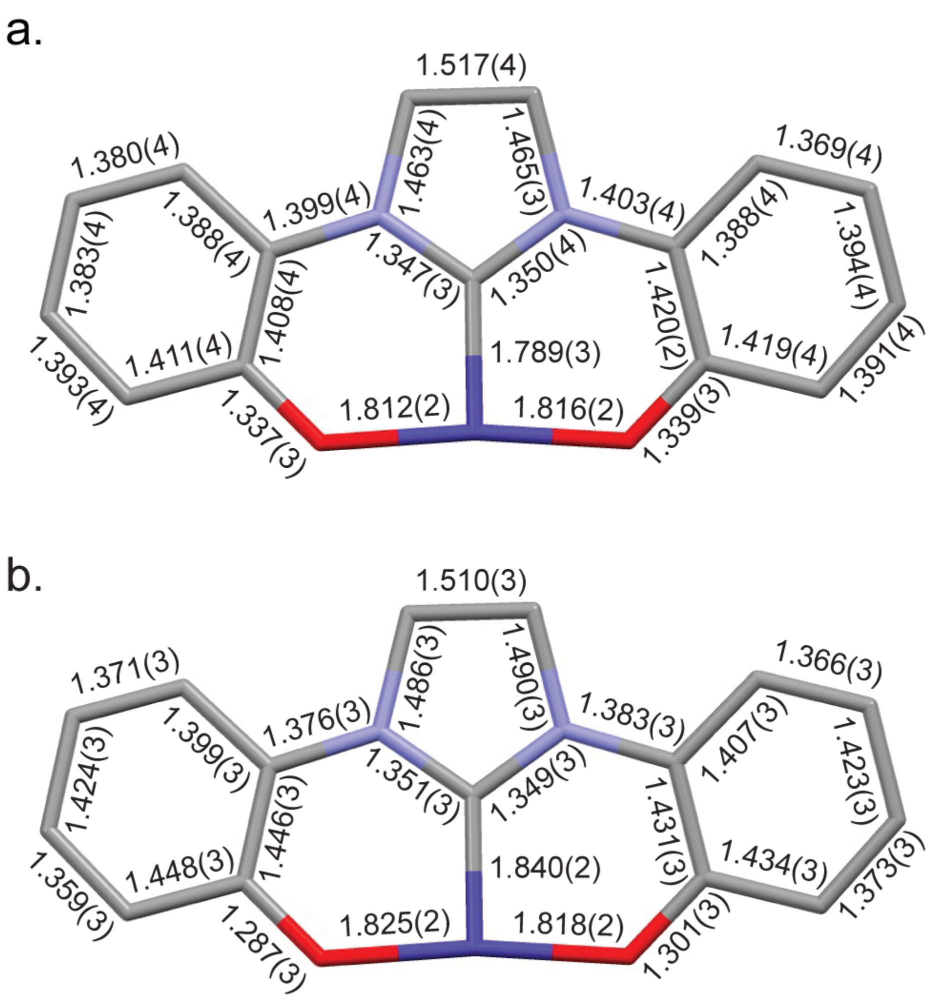

Figure S30. Schematic of selected bond lengths (Å) for a) **III** and b) $[(OCO^0)Co^{II}(THF)_3](PF_6)_2$.[2] Reproduced with permission from reference 2. Copyright 2017 American Chemical Society.

X–Ray Structure Report:

[(OCO)Co(CF_3)(THF)OTf] (II)

Experimental: The data for **II** were collected from a single crystal at 100(2) K on a Bruker D8 VENTURE dual wavelength Mo/Cu four-circle diffractometer with a microfocus sealed X-ray tube using a mirror optics as monochromator and a Bruker PHOTON II detector. The diffractometer was equipped with an Oxford Cryostream 800 low temperature device and used MoK_α radiation (λ = 0.71073 Å). All data were integrated with SAINT V8.40B and a multi-scan absorption correction using SADABS 2016/2 was applied.[3, 4] The structure was solved by dual methods with SHELXT and refined by full-matrix least-squares methods against F^2 using SHELXL-2014.[5, 6] All atoms were refined with anisotropic displacement parameters. Hydrogen atom positions were located from the electron densities and freely refined using Hirshfeld scattering factors. Refinement was by using NoSpherA2, an implementation of non-spherical atom-form-factors.[7] This report and the CIF file were generated using FinalCif.[8]

Table S1. Crystal data and structure refinement for **II**

CCDC number	
Empirical formula	$C_{80}H_{122}Co_2F_{12}N_4O_{13}S_2Si_2$
Formula weight	1814.030
Temperature [K]	100(2)
Crystal system	monoclinic
Space group (number)	$C2/c$ (15)
a [Å]	37.631(5)
b [Å]	14.3738(17)
c [Å]	18.538(2)
α [°]	90
β [°]	115.665(5)
γ [°]	90
Volume [Å^3]	9038(2)
Z	4
ρ_{calc} [gcm^{-3}]	1.333
μ [mm^{-1}]	0.523
F(000)	3831.308
Crystal size [mm^3]	0.226×0.283×0.406
Crystal colour	gold
Crystal shape	prism
Radiation	MoK_α (λ=0.71073 Å)
2θ range [°]	4.24 to 74.12 (0.59 Å)
Index ranges	−63 ≤ h ≤ 63<br>−24 ≤ k ≤ 24<br>−31 ≤ l ≤ 30
Reflections collected	133469
Independent reflections	23022<br>R_{int} = 0.0642<br>R_{sigma} = 0.0442
Completeness to θ = 25.2417°	99.7 %
Data / Restraints / Parameters	23022 / 870 / 1068
Goodness-of-fit on F^2	1.0607
Final *R* indexes [I≥2σ(I)]	R_1 = 0.0463<br>wR_2 = 0.1039
Final *R* indexes [all data]	R_1 = 0.0705<br>wR_2 = 0.1206
Largest peak/hole [eÅ^{-3}]	0.91/−0.80

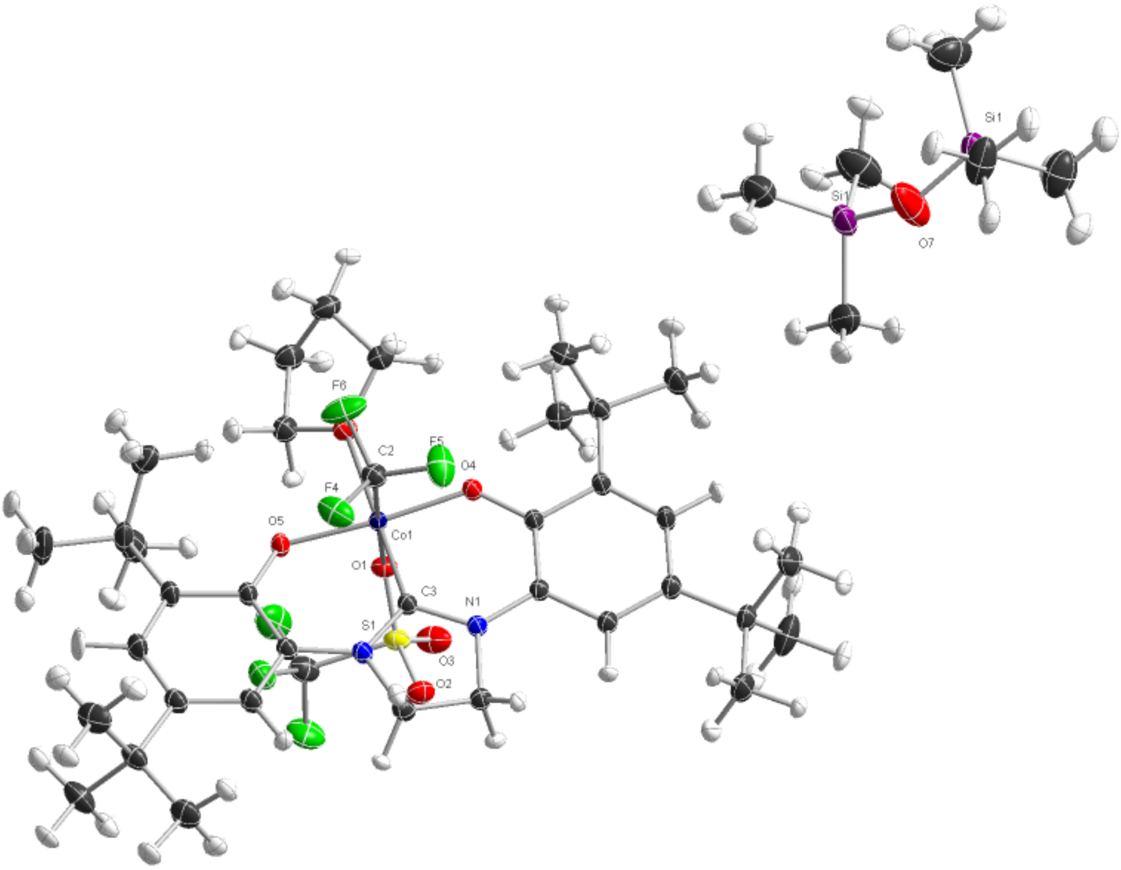

Table S2. Atomic coordinates and Ueq [Å^2] for **II**

Atom	*x*	*y*	*z*	U_{eq}
Co1	0.210080(4)	0.394442(10)	0.326509(9)	0.01407(3)
S1	0.228355(9)	0.37257(2)	0.166967(18)	0.01951(5)
F1	0.15399(3)	0.31971(7)	0.09679(5)	0.03223(18)
F2	0.16550(3)	0.45194(7)	0.05651(6)	0.0443(2)
F3	0.18278(3)	0.32423(8)	0.01924(5)	0.0417(2)
F4	0.17952(3)	0.29846(7)	0.41625(5)	0.0355(2)
F5	0.23659(3)	0.35373(9)	0.48623(5)	0.0422(2)
F6	0.18610(4)	0.44327(7)	0.44075(7)	0.0447(3)
O1	0.21958(3)	0.42659(6)	0.22404(5)	0.02045(15)
O2	0.23849(3)	0.27693(6)	0.18924(6)	0.02469(17)
O3	0.25216(4)	0.42190(7)	0.13673(7)	0.0335(2)
O4	0.26168(2)	0.43242(6)	0.38597(5)	0.01781(14)
O5	0.15723(2)	0.37212(6)	0.26224(5)	0.01831(14)
O6	0.19522(3)	0.53478(6)	0.31840(6)	0.02320(17)
N1	0.26085(3)	0.23744(6)	0.36299(6)	0.01635(15)
N2	0.19796(3)	0.20063(6)	0.30060(6)	0.01709(16)
C1	0.17985(4)	0.36664(10)	0.08009(8)	0.0274(2)
C37	0.20266(4)	0.37126(9)	0.42108(7)	0.0219(2)
C4	0.22363(3)	0.27136(7)	0.32985(6)	0.01573(17)
C19	0.21824(3)	0.10971(8)	0.31945(7)	0.01970(19)
H19B	0.2114(5)	0.0841(13)	0.3651(11)	0.031(4)
H19A	0.2073(5)	0.0661(13)	0.2677(11)	0.032(4)

C20	0.26121(3)	0.13646(8)	0.34791(7)	0.01888(18)
H20B	0.2718(6)	0.1253(13)	0.3000(11)	0.030(4)
H20A	0.2792(5)	0.0991(12)	0.3993(11)	0.030(3)
C5	0.15693(3)	0.20576(8)	0.26412(7)	0.01787(18)
C6	0.13772(3)	0.29377(8)	0.24566(7)	0.01791(18)
C7	0.09525(3)	0.29434(9)	0.20669(8)	0.0229(2)
C8	0.07569(3)	0.20959(9)	0.18927(8)	0.0249(2)
H8	0.0436(6)	0.2151(15)	0.1596(17)	0.073(8)
C9	0.09467(4)	0.12238(9)	0.20822(8)	0.0215(2)
C10	0.13536(3)	0.12205(8)	0.24597(7)	0.02024(19)
H10	0.1515(5)	0.0570(12)	0.2631(12)	0.035(4)
C11	0.07001(4)	0.03309(9)	0.18685(8)	0.0247(2)
C12	0.09576(5)	−0.05472(10)	0.20786(11)	0.0340(3)
H12C	0.1158(6)	−0.0612(15)	0.2735(13)	0.045(4)
H12B	0.0748(6)	−0.1157(14)	0.1870(14)	0.046(4)
H12A	0.1142(6)	−0.0583(15)	0.1754(14)	0.046(4)
C13	0.04305(5)	0.03122(12)	0.09672(9)	0.0349(3)
H13B	0.0241(6)	−0.0320(15)	0.0806(13)	0.047(4)
H13A	0.0229(7)	0.0912(15)	0.0793(14)	0.051(4)
H13C	0.0608(7)	0.0339(16)	0.0633(13)	0.051(5)
C14	0.04479(5)	0.03001(12)	0.23313(11)	0.0364(3)
H14B	0.0275(7)	−0.0333(15)	0.2207(13)	0.048(4)
H14A	0.0637(7)	0.0323(16)	0.2973(14)	0.053(4)
H14C	0.0261(7)	0.0899(15)	0.2198(15)	0.050(4)
C15	0.07241(4)	0.38622(10)	0.18444(10)	0.0300(3)
C16	0.08226(5)	0.43797(12)	0.12302(10)	0.0362(3)
H16A	0.1128(6)	0.4467(14)	0.1398(12)	0.043(4)
H16C	0.0721(8)	0.3990(16)	0.0667(14)	0.059(5)
H16B	0.0678(6)	0.5050(15)	0.1120(13)	0.049(4)
C18	0.02762(4)	0.37002(13)	0.14614(17)	0.0546(6)
H18C	0.0157(7)	0.3308(17)	0.1835(17)	0.063(5)
H18A	0.0196(7)	0.3322(17)	0.0898(18)	0.069(5)
H18B	0.0126(6)	0.4388(16)	0.1303(17)	0.064(5)
C17	0.08218(4)	0.44559(11)	0.25988(11)	0.0335(3)
H17B	0.0683(6)	0.5114(15)	0.2418(14)	0.052(4)
H17C	0.1140(6)	0.4560(14)	0.2926(13)	0.042(4)
H17A	0.0716(6)	0.4119(15)	0.2994(14)	0.045(4)
C3	0.29557(3)	0.28713(8)	0.40223(6)	0.01647(17)
C2	0.29433(3)	0.38524(7)	0.41417(7)	0.01668(17)
C24	0.33141(3)	0.43230(8)	0.45886(7)	0.01834(18)
C23	0.36561(3)	0.38086(8)	0.48664(8)	0.0220(2)
H23	0.3935(5)	0.4174(13)	0.5172(13)	0.042(5)
C22	0.36685(3)	0.28379(8)	0.47510(7)	0.02028(19)
C21	0.33165(3)	0.23862(8)	0.43204(7)	0.01894(19)
H21	0.3307(5)	0.1643(12)	0.4236(11)	0.033(4)
C25	0.33200(3)	0.53689(8)	0.47400(7)	0.0208(2)
C26	0.31433(4)	0.58795(9)	0.39327(9)	0.0262(2)
H26B	0.3116(6)	0.6614(14)	0.4002(13)	0.043(4)
H26C	0.2867(5)	0.5622(13)	0.3548(11)	0.033(3)
H26A	0.3326(6)	0.5791(15)	0.3617(12)	0.040(4)
C28	0.30990(4)	0.55822(10)	0.52497(8)	0.0282(2)
H28C	0.3072(6)	0.6325(14)	0.5277(13)	0.045(4)
H28A	0.3267(6)	0.5303(15)	0.5870(12)	0.040(4)

H28B	0.2806(6)	0.5282(14)	0.5010(12)	0.038(4)
C27	0.37416(4)	0.57438(10)	0.51998(10)	0.0320(3)
H27C	0.3900(6)	0.5635(15)	0.4821(13)	0.041(4)
H27B	0.3728(7)	0.6500(14)	0.5288(14)	0.051(4)
H27A	0.3895(6)	0.5448(15)	0.5803(13)	0.044(4)
C29	0.40662(3)	0.23375(9)	0.51223(8)	0.0235(2)
C31	0.40216(4)	0.12811(11)	0.50366(11)	0.0347(3)
H31B	0.3870(7)	0.1073(15)	0.4401(14)	0.049(4)
H31A	0.4318(6)	0.0956(14)	0.5320(13)	0.042(4)
H31C	0.3844(6)	0.1025(13)	0.5335(13)	0.038(4)
C30	0.43223(5)	0.26691(14)	0.47180(12)	0.0407(4)
H30B	0.4619(6)	0.2353(17)	0.4972(14)	0.052(4)
H30A	0.4164(7)	0.2472(18)	0.4069(14)	0.058(4)
H30C	0.4369(6)	0.3432(17)	0.4792(15)	0.053(4)
C32	0.42728(5)	0.25659(12)	0.60221(9)	0.0353(3)
H32A	0.4105(6)	0.2459(16)	0.6342(12)	0.047(4)
H32B	0.4337(6)	0.3301(15)	0.6113(13)	0.050(4)
H32C	0.4557(6)	0.2188(16)	0.6320(13)	0.049(4)
C33	0.21399(4)	0.60749(9)	0.37898(9)	0.0270(2)
H33B	0.2177(6)	0.5774(15)	0.4367(12)	0.044(4)
H33A	0.2439(6)	0.6233(14)	0.3794(13)	0.041(4)
C34	0.18618(5)	0.68934(10)	0.35132(9)	0.0294(3)
H34A	0.1993(6)	0.7590(14)	0.3747(12)	0.038(4)
H34B	0.1609(6)	0.6802(15)	0.3688(13)	0.042(4)
C35	0.16990(4)	0.68169(9)	0.26077(9)	0.0278(2)
H35A	0.1898(6)	0.7119(14)	0.2391(12)	0.039(4)
H35B	0.1444(6)	0.7196(15)	0.2311(12)	0.044(4)
C36	0.16550(4)	0.57724(9)	0.24704(8)	0.0252(2)
H36B	0.1723(6)	0.5537(15)	0.1975(12)	0.043(4)
H36A	0.1354(6)	0.5542(15)	0.2419(13)	0.045(4)
Si1	0.467826(13)	0.77576(3)	0.66123(3)	0.03664(10)
O7	0.5	0.74450(16)	0.75	0.0743(8)
C38	0.46919(6)	0.68825(16)	0.58858(17)	0.0562(6)
H38A	0.4967(7)	0.6897(19)	0.5936(18)	0.067(5)
H38C	0.4652(8)	0.6171(19)	0.6056(18)	0.063(5)
H38B	0.4453(8)	0.707(2)	0.5260(18)	0.070(5)
C40	0.48007(9)	0.89202(16)	0.63633(16)	0.0616(6)
H40A	0.4825(9)	0.9492(19)	0.6803(17)	0.075(6)
H40C	0.5085(9)	0.8826(19)	0.6245(17)	0.069(6)
H40B	0.4590(9)	0.9134(19)	0.5788(18)	0.072(5)
C39	0.41855(7)	0.77939(18)	0.66182(16)	0.0568(6)
H39A	0.4084(8)	0.711(2)	0.6721(17)	0.068(5)
H39C	0.4178(9)	0.827(2)	0.7061(18)	0.078(5)
H39B	0.3953(9)	0.805(2)	0.6014(18)	0.075(5)

U_{eq} is defined as 1/3 of the trace of the orthogonalized U_{ij} tensor.

Table S3. Anisotropic displacement parameters (Å^2) for **II**. The anisotropic displacement factor exponent takes the form: $-2\pi^2[h^2(a^*)^2U_{11} + k^2(b^*)^2U_{22} + \ldots + 2hka^*b^*U_{12}]$

Atom	U_{11}	U_{22}	U_{33}	U_{23}	U_{13}	U_{12}
Co1	0.01220(6)	0.01333(6)	0.01529(6)	−0.00015(5)	0.00466(5)	−0.00015(4)
S1	0.02414(12)	0.01556(11)	0.02165(12)	−0.00120(9)	0.01257(11)	−0.00233(9)
F1	0.0279(4)	0.0334(4)	0.0299(4)	−0.0040(3)	0.0074(3)	−0.0040(3)
F2	0.0535(6)	0.0314(5)	0.0341(5)	0.0112(4)	0.0059(5)	0.0091(4)
F3	0.0560(6)	0.0468(6)	0.0214(4)	−0.0067(4)	0.0161(4)	−0.0052(5)
F4	0.0464(5)	0.0390(5)	0.0290(4)	−0.0056(4)	0.0236(4)	−0.0174(4)
F5	0.0301(4)	0.0750(8)	0.0198(4)	0.0090(4)	0.0094(3)	−0.0013(5)
F6	0.0729(7)	0.0322(5)	0.0518(6)	0.0032(4)	0.0483(6)	0.0115(5)
O1	0.0250(4)	0.0168(3)	0.0209(4)	−0.0013(3)	0.0112(3)	−0.0004(3)
O2	0.0293(4)	0.0181(4)	0.0270(4)	−0.0002(3)	0.0125(4)	0.0025(3)
O3	0.0446(6)	0.0267(5)	0.0437(6)	−0.0052(4)	0.0326(5)	−0.0102(4)
O4	0.0150(3)	0.0144(3)	0.0205(4)	−0.0006(3)	0.0044(3)	−0.0001(3)
O5	0.0132(3)	0.0165(3)	0.0219(4)	0.0008(3)	0.0045(3)	−0.0009(3)
O6	0.0235(4)	0.0184(4)	0.0242(4)	−0.0009(3)	0.0070(3)	0.0033(3)
N1	0.0150(3)	0.0146(3)	0.0176(4)	0.0002(3)	0.0054(3)	0.0000(3)
N2	0.0159(4)	0.0151(4)	0.0186(4)	−0.0004(3)	0.0058(3)	−0.0017(3)
C1	0.0348(7)	0.0249(5)	0.0190(5)	0.0012(4)	0.0085(5)	0.0004(5)
C37	0.0234(5)	0.0235(5)	0.0210(5)	−0.0010(4)	0.0117(4)	−0.0012(4)
C4	0.0143(4)	0.0144(4)	0.0173(4)	−0.0001(3)	0.0058(3)	−0.0011(3)
C19	0.0198(4)	0.0158(4)	0.0216(5)	−0.0002(4)	0.0071(4)	−0.0016(3)
H19B	0.028(7)	0.037(7)	0.026(4)	0.004(2)	0.011(2)	−0.006(3)
H19A	0.034(7)	0.028(6)	0.027(4)	−0.008(2)	0.008(2)	−0.002(3)
C20	0.0180(4)	0.0157(4)	0.0215(5)	−0.0002(4)	0.0073(4)	0.0008(3)
H20B	0.035(7)	0.028(8)	0.033(4)	−0.001(3)	0.020(3)	0.002(4)
H20A	0.028(6)	0.027(6)	0.028(4)	0.006(2)	0.0066(19)	0.004(3)
C5	0.0158(4)	0.0175(4)	0.0193(4)	−0.0015(3)	0.0066(4)	−0.0033(3)
C6	0.0135(4)	0.0180(4)	0.0200(5)	−0.0007(4)	0.0052(4)	−0.0021(3)
C7	0.0132(4)	0.0218(5)	0.0305(6)	−0.0026(4)	0.0064(4)	−0.0029(3)
C8	0.0148(4)	0.0236(5)	0.0334(6)	−0.0033(4)	0.0078(4)	−0.0048(4)
H8	0.016(2)	0.044(9)	0.13(2)	0.003(5)	0.0056(13)	−0.0041(13)
C9	0.0187(4)	0.0213(5)	0.0241(5)	−0.0032(4)	0.0087(4)	−0.0063(3)
C10	0.0182(4)	0.0180(5)	0.0232(5)	−0.0015(4)	0.0077(4)	−0.0044(3)
H10	0.030(6)	0.022(3)	0.050(11)	0.003(3)	0.014(4)	0.002(2)
C11	0.0236(5)	0.0251(5)	0.0252(5)	−0.0050(4)	0.0104(4)	−0.0107(4)
C12	0.0320(7)	0.0229(6)	0.0434(8)	−0.0047(5)	0.0128(6)	−0.0086(5)
H12C	0.043(6)	0.038(9)	0.046(3)	−0.0032(16)	0.0109(15)	−0.008(3)
H12B	0.044(6)	0.030(4)	0.058(9)	−0.006(3)	0.016(3)	−0.016(2)
H12A	0.047(6)	0.035(9)	0.061(7)	−0.005(4)	0.028(3)	−0.008(3)
C13	0.0350(7)	0.0348(7)	0.0279(6)	−0.0048(5)	0.0070(5)	−0.0147(6)
H13B	0.048(7)	0.042(4)	0.047(9)	−0.010(3)	0.016(3)	−0.023(2)
H13A	0.045(6)	0.041(4)	0.057(9)	−0.000(3)	0.014(3)	−0.008(2)
H13C	0.052(6)	0.057(10)	0.045(7)	−0.006(4)	0.023(3)	−0.017(3)
C14	0.0383(7)	0.0371(8)	0.0429(8)	−0.0081(6)	0.0261(6)	−0.0165(6)
H14B	0.054(7)	0.044(4)	0.056(8)	−0.011(3)	0.033(4)	−0.025(2)
H14A	0.060(7)	0.055(9)	0.045(2)	−0.0089(15)	0.0240(14)	−0.016(3)
H14C	0.049(6)	0.044(4)	0.069(9)	−0.003(3)	0.036(3)	−0.009(2)
C15	0.0139(4)	0.0242(6)	0.0440(8)	−0.0007(5)	0.0050(4)	0.0001(4)
C16	0.0305(7)	0.0322(7)	0.0325(7)	0.0040(5)	0.0010(5)	0.0028(6)
H16A	0.033(2)	0.035(8)	0.047(8)	0.004(4)	0.0059(15)	0.0022(15)
H16C	0.065(9)	0.055(8)	0.044(4)	−0.009(2)	0.013(2)	−0.009(4)

H16B	0.046(8)	0.037(3)	0.054(9)	0.008(2)	0.011(4)	0.009(2)
C18	0.0155(5)	0.0321(8)	0.0957(17)	−0.0061(9)	0.0049(7)	−0.0002(5)
H18C	0.032(8)	0.036(8)	0.106(8)	−0.008(3)	0.018(4)	−0.005(4)
H18A	0.034(9)	0.049(8)	0.098(4)	−0.012(3)	0.003(2)	0.009(4)
H18B	0.025(7)	0.036(3)	0.115(11)	−0.001(2)	0.015(4)	0.005(2)
C17	0.0257(6)	0.0267(6)	0.0517(9)	−0.0026(6)	0.0202(6)	0.0008(5)
H17B	0.045(7)	0.033(3)	0.083(9)	0.005(2)	0.030(4)	0.009(2)
H17C	0.027(2)	0.039(9)	0.059(7)	−0.004(4)	0.0195(14)	−0.0011(14)
H17A	0.044(8)	0.038(8)	0.064(6)	−0.001(3)	0.032(3)	−0.003(3)
C3	0.0145(4)	0.0160(4)	0.0169(4)	0.0005(3)	0.0049(3)	0.0006(3)
C2	0.0141(4)	0.0152(4)	0.0181(4)	−0.0001(3)	0.0046(3)	−0.0005(3)
C24	0.0142(4)	0.0158(4)	0.0204(5)	−0.0006(3)	0.0032(3)	−0.0006(3)
C23	0.0134(4)	0.0191(5)	0.0265(5)	−0.0011(4)	0.0020(4)	−0.0004(3)
H23	0.018(3)	0.028(7)	0.062(13)	−0.007(4)	0.001(2)	−0.005(2)
C22	0.0148(4)	0.0190(5)	0.0228(5)	0.0011(4)	0.0042(4)	0.0019(3)
C21	0.0155(4)	0.0172(4)	0.0212(5)	0.0003(4)	0.0052(4)	0.0020(3)
H21	0.032(8)	0.017(2)	0.041(10)	−0.0024(14)	0.009(5)	0.0024(13)
C25	0.0187(4)	0.0166(4)	0.0230(5)	−0.0021(4)	0.0052(4)	−0.0020(3)
C26	0.0253(5)	0.0214(5)	0.0299(6)	0.0045(4)	0.0102(5)	−0.0012(4)
H26B	0.047(8)	0.024(2)	0.058(9)	0.0018(14)	0.023(4)	−0.0003(14)
H26C	0.028(3)	0.036(7)	0.034(5)	0.000(3)	0.0113(16)	−0.0050(19)
H26A	0.033(5)	0.051(9)	0.038(6)	0.003(3)	0.017(3)	0.001(3)
C28	0.0284(6)	0.0287(6)	0.0253(6)	−0.0078(5)	0.0096(5)	−0.0023(5)
H28C	0.048(8)	0.031(2)	0.056(9)	−0.0089(13)	0.023(4)	−0.0010(13)
H28A	0.038(7)	0.047(8)	0.029(3)	−0.0017(19)	0.0106(17)	0.000(3)
H28B	0.032(3)	0.043(8)	0.036(7)	−0.010(4)	0.0121(18)	−0.008(2)
C27	0.0220(5)	0.0229(6)	0.0414(8)	−0.0064(5)	0.0047(5)	−0.0051(4)
H27C	0.034(6)	0.033(9)	0.053(6)	−0.008(3)	0.016(3)	−0.006(4)
H27B	0.051(9)	0.025(2)	0.074(9)	−0.0100(14)	0.023(4)	−0.0049(14)
H27A	0.036(7)	0.039(8)	0.045(3)	−0.001(2)	0.0068(19)	−0.001(3)
C29	0.0152(4)	0.0244(5)	0.0258(5)	0.0022(4)	0.0040(4)	0.0043(4)
C31	0.0228(6)	0.0260(6)	0.0447(8)	−0.0017(5)	0.0046(6)	0.0081(5)
H31B	0.037(7)	0.049(9)	0.048(3)	−0.0080(17)	0.0051(16)	0.005(4)
H31A	0.026(3)	0.034(7)	0.054(8)	−0.001(3)	0.0053(19)	0.011(2)
H31C	0.028(6)	0.025(8)	0.054(7)	0.002(3)	0.011(3)	0.011(3)
C30	0.0239(6)	0.0512(10)	0.0494(9)	0.0175(8)	0.0183(6)	0.0119(6)
H30B	0.029(3)	0.065(8)	0.062(8)	0.015(4)	0.019(2)	0.018(2)
H30A	0.045(7)	0.073(10)	0.052(3)	0.0123(19)	0.0187(16)	0.009(4)
H30C	0.034(8)	0.053(3)	0.079(10)	0.0139(16)	0.031(4)	0.0091(14)
C32	0.0263(6)	0.0375(8)	0.0283(6)	−0.0010(5)	−0.0012(5)	0.0096(6)
H32A	0.039(6)	0.056(9)	0.040(6)	0.003(3)	0.010(3)	0.012(3)
H32B	0.049(9)	0.041(2)	0.048(9)	−0.0047(15)	0.009(4)	0.0050(15)
H32C	0.033(4)	0.054(8)	0.044(8)	0.003(3)	0.001(2)	0.017(2)
C33	0.0259(5)	0.0244(5)	0.0293(6)	−0.0031(4)	0.0108(5)	0.0023(4)
H33B	0.055(9)	0.041(8)	0.035(3)	0.002(2)	0.020(2)	0.002(4)
H33A	0.033(3)	0.035(8)	0.061(9)	−0.007(4)	0.024(2)	−0.002(2)
C34	0.0332(6)	0.0256(6)	0.0307(6)	−0.0015(5)	0.0151(5)	0.0061(5)
H34A	0.052(8)	0.028(3)	0.038(8)	−0.006(2)	0.025(4)	−0.001(2)
H34B	0.042(4)	0.042(9)	0.051(8)	0.005(4)	0.028(3)	0.008(3)
C35	0.0321(6)	0.0219(5)	0.0295(6)	0.0031(4)	0.0134(5)	0.0051(4)
H35A	0.047(5)	0.034(8)	0.045(7)	0.001(3)	0.027(3)	−0.001(3)
H35B	0.043(4)	0.051(7)	0.041(7)	0.016(3)	0.021(2)	0.021(2)
C36	0.0232(5)	0.0232(5)	0.0265(6)	0.0006(4)	0.0083(4)	0.0017(4)

H36B	0.047(8)	0.049(9)	0.034(4)	0.000(3)	0.020(3)	0.013(4)
H36A	0.031(3)	0.046(9)	0.060(9)	−0.003(4)	0.021(2)	−0.005(2)
Si1	0.02485(18)	0.0290(2)	0.0434(2)	−0.00419(17)	0.00290(17)	−0.00323(15)
O7	0.0640(14)	0.0460(12)	0.0629(13)	0.000000	−0.0194(10)	−0.000000
C38	0.0343(8)	0.0488(11)	0.0848(16)	−0.0263(10)	0.0253(9)	−0.0069(8)
H38A	0.038(3)	0.066(10)	0.098(11)	−0.024(4)	0.030(2)	−0.007(2)
H38C	0.041(9)	0.053(3)	0.092(10)	−0.024(2)	0.025(4)	−0.009(2)
H38B	0.043(7)	0.073(11)	0.087(4)	−0.022(2)	0.024(2)	−0.002(4)
C40	0.0768(16)	0.0405(10)	0.0591(14)	−0.0031(9)	0.0214(12)	−0.0201(10)
H40A	0.105(13)	0.046(6)	0.067(7)	−0.007(3)	0.031(4)	−0.019(4)
H40C	0.079(5)	0.054(11)	0.066(11)	−0.004(5)	0.025(3)	−0.020(3)
H40B	0.090(7)	0.052(10)	0.061(4)	−0.003(2)	0.020(2)	−0.009(3)
C39	0.0440(10)	0.0569(13)	0.0709(14)	−0.0263(11)	0.0262(9)	−0.0122(8)
H39A	0.065(10)	0.062(4)	0.083(10)	−0.027(2)	0.037(4)	−0.019(2)
H39C	0.080(11)	0.073(7)	0.086(7)	−0.036(3)	0.041(4)	−0.010(4)
H39B	0.058(7)	0.084(11)	0.077(4)	−0.020(3)	0.025(2)	−0.003(3)

Table S4. Bond lengths and angles for **II**

Atom–Atom	Length [Å]
Co1–O1	2.1302(9)
Co1–O4	1.8518(8)
Co1–O5	1.8506(8)
Co1–O6	2.0812(9)
Co1–C37	1.9201(12)
Co1–C4	1.8347(11)
S1–O1	1.4610(9)
S1–O2	1.4385(10)
S1–O3	1.4332(10)
S1–C1	1.8402(15)
F1–C1	1.3261(17)
F2–C1	1.3348(17)
F3–C1	1.3282(16)
F4–C37	1.3390(15)
F5–C37	1.3480(16)
F6–C37	1.3376(15)
O4–C2	1.2986(13)
O5–C6	1.3063(13)
O6–C33	1.4716(16)
O6–C36	1.4462(16)
N1–C4	1.3533(14)
N1–C20	1.4793(15)
N1–C3	1.3869(14)
N2–C4	1.3444(14)
N2–C19	1.4769(15)
N2–C5	1.3935(14)
C19–H19B	1.052(18)
C19–H19A	1.068(17)
C19–C20	1.5173(16)
C20–H20B	1.130(18)
C20–H20A	1.046(17)
C5–C6	1.4232(16)
C5–C10	1.4084(16)
C6–C7	1.4409(16)
C7–C8	1.3871(17)
C7–C15	1.5318(18)
C8–H8	1.092(19)
C8–C9	1.4100(18)
C9–C10	1.3804(17)
C9–C11	1.5320(16)
C10–H10	1.086(18)
C11–C12	1.535(2)
C11–C13	1.534(2)
C11–C14	1.531(2)
C12–H12C	1.12(2)
C12–H12B	1.130(19)
C12–H12A	1.10(2)
C13–H13B	1.11(2)
C13–H13A	1.10(2)
C13–H13C	1.09(2)
C14–H14B	1.08(2)
C14–H14A	1.09(2)
C14–H14C	1.07(2)
C15–C16	1.534(2)
C15–C18	1.537(2)
C15–C17	1.541(2)
C16–H16A	1.06(2)
C16–H16C	1.10(2)
C16–H16B	1.08(2)
C18–H18C	1.12(3)
C18–H18A	1.10(3)
C18–H18B	1.11(2)
C17–H17B	1.06(2)
C17–H17C	1.093(19)
C17–H17A	1.09(2)
C3–C2	1.4314(15)
C3–C21	1.4089(15)

C2–C24	1.4456(15)
C24–C23	1.3764(16)
C24–C25	1.5278(16)
C23–H23	1.088(17)
C23–C22	1.4153(17)
C22–C21	1.3775(16)
C22–C29	1.5291(16)
C21–H21	1.078(17)
C25–C26	1.5358(18)
C25–C28	1.5362(19)
C25–C27	1.5369(18)
C26–H26B	1.074(19)
C26–H26C	1.043(18)
C26–H26A	1.087(19)
C28–H28C	1.08(2)
C28–H28A	1.120(19)
C28–H28B	1.083(19)
C27–H27C	1.11(2)
C27–H27B	1.10(2)
C27–H27A	1.10(2)
C29–C31	1.528(2)
C29–C30	1.531(2)
C29–C32	1.540(2)
C31–H31B	1.11(2)
C31–H31A	1.110(19)
C31–H31C	1.10(2)
C30–H30B	1.11(2)
C30–H30A	1.12(2)
C30–H30C	1.11(2)
C32–H32A	1.05(2)
C32–H32B	1.08(2)
C32–H32C	1.11(2)
C33–H33B	1.11(2)
C33–H33A	1.144(19)
C33–C34	1.5090(19)
C34–H34A	1.12(2)
C34–H34B	1.14(2)
C34–C35	1.521(2)
C35–H35A	1.08(2)
C35–H35B	1.03(2)
C35–C36	1.5198(19)
C36–H36B	1.108(19)
C36–H36A	1.144(19)
Si1–O7[#1]	1.6279(8)
Si1–C38	1.860(2)
Si1–C40	1.844(2)
Si1–C39	1.860(2)
C38–H38A	1.00(2)
C38–H38C	1.10(3)
C38–H38B	1.15(3)
C40–H40A	1.13(3)
C40–H40C	1.19(3)
C40–H40B	1.06(3)
C39–H39A	1.10(3)
C39–H39C	1.08(3)
C39–H39B	1.14(3)

**Atom–Atom–Atom**	**Angle [°]**
O4–Co1–O1	85.94(4)
O5–Co1–O1	90.69(4)
O5–Co1–O4	172.59(4)
O6–Co1–O1	82.47(4)
O6–Co1–O4	86.39(4)
O6–Co1–O5	86.63(4)
C37–Co1–O1	177.18(4)
C37–Co1–O4	92.14(5)
C37–Co1–O5	90.97(5)
C37–Co1–O6	95.36(5)
C4–Co1–O1	95.03(4)
C4–Co1–O4	93.42(4)
C4–Co1–O5	93.44(4)
C4–Co1–O6	177.50(4)
C4–Co1–C37	87.14(5)
O2–S1–O1	114.52(5)
O3–S1–O1	113.33(6)
O3–S1–O2	116.88(6)
C1–S1–O1	102.05(6)
C1–S1–O2	104.25(6)
C1–S1–O3	103.39(7)
S1–O1–Co1	135.10(5)
C2–O4–Co1	130.61(7)
C6–O5–Co1	129.70(7)
C33–O6–Co1	127.45(8)
C36–O6–Co1	123.52(8)
C36–O6–C33	108.89(10)
C20–N1–C4	111.11(9)
C3–N1–C4	127.39(9)
C3–N1–C20	121.38(9)
C19–N2–C4	111.49(9)
C5–N2–C4	127.39(10)
C5–N2–C19	120.80(9)
F1–C1–S1	111.67(9)
F2–C1–S1	110.57(10)
F2–C1–F1	108.05(13)
F3–C1–S1	110.42(11)
F3–C1–F1	107.70(12)
F3–C1–F2	108.31(11)
F4–C37–Co1	114.96(9)
F5–C37–Co1	113.24(8)
F5–C37–F4	104.48(11)
F6–C37–Co1	112.57(9)
F6–C37–F4	104.88(10)
F6–C37–F5	105.82(11)
N1–C4–Co1	125.50(8)
N2–C4–Co1	125.09(8)
N2–C4–N1	109.40(9)
H19B–C19–N2	102.7(11)
H19A–C19–N2	110.4(10)
H19A–C19–H19B	112.6(15)

C20–C19–N2	102.82(9)
C20–C19–H19B	113.8(10)
C20–C19–H19A	113.5(10)
C19–C20–N1	102.63(9)
H20B–C20–N1	108.9(9)
H20B–C20–C19	112.1(10)
H20A–C20–N1	112.8(10)
H20A–C20–C19	111.0(10)
H20A–C20–H20B	109.4(14)
C6–C5–N2	120.29(10)
C10–C5–N2	118.27(10)
C10–C5–C6	121.44(10)
C5–C6–O5	122.31(10)
C7–C6–O5	120.10(10)
C7–C6–C5	117.59(10)
C8–C7–C6	118.25(11)
C15–C7–C6	120.74(10)
C15–C7–C8	121.01(11)
H8–C8–C7	114.4(12)
C9–C8–C7	124.21(11)
C9–C8–H8	121.3(12)
C10–C9–C8	117.41(11)
C11–C9–C8	119.70(11)
C11–C9–C10	122.89(12)
C9–C10–C5	121.08(11)
H10–C10–C5	118.3(10)
H10–C10–C9	120.6(10)
C12–C11–C9	112.20(11)
C13–C11–C9	109.70(11)
C13–C11–C12	108.00(12)
C14–C11–C9	109.42(11)
C14–C11–C12	108.08(13)
C14–C11–C13	109.38(12)
H12C–C12–C11	113.4(11)
H12B–C12–C11	106.2(11)
H12B–C12–H12C	110.4(16)
H12A–C12–C11	112.2(12)
H12A–C12–H12C	107.5(16)
H12A–C12–H12B	106.9(16)
H13B–C13–C11	110.5(11)
H13A–C13–C11	110.6(12)
H13A–C13–H13B	106.4(16)
H13C–C13–C11	109.8(12)
H13C–C13–H13B	110.8(17)
H13C–C13–H13A	108.6(17)
H14B–C14–C11	111.5(11)
H14A–C14–C11	110.0(12)
H14A–C14–H14B	107.7(17)
H14C–C14–C11	110.6(12)
H14C–C14–H14B	110.6(17)
H14C–C14–H14A	106.3(18)
C16–C15–C7	109.27(12)
C18–C15–C7	111.65(12)
C18–C15–C16	107.44(15)
C17–C15–C7	110.35(12)
C17–C15–C16	111.46(12)
C17–C15–C18	106.62(14)
H16A–C16–C15	114.8(11)
H16C–C16–C15	111.2(13)
H16C–C16–H16A	103.0(18)
H16B–C16–C15	108.0(12)
H16B–C16–H16A	110.1(16)
H16B–C16–H16C	109.6(17)
H18C–C18–C15	116.1(13)
H18A–C18–C15	108.0(13)
H18A–C18–H18C	108.6(19)
H18B–C18–C15	108.4(11)
H18B–C18–H18C	108.5(18)
H18B–C18–H18A	107(2)
H17B–C17–C15	108.3(13)
H17C–C17–C15	110.7(11)
H17C–C17–H17B	108.9(16)
H17A–C17–C15	110.6(12)
H17A–C17–H17B	109.6(16)
H17A–C17–H17C	108.6(16)
C2–C3–N1	120.13(9)
C21–C3–N1	118.67(10)
C21–C3–C2	121.16(10)
C3–C2–O4	122.75(10)
C24–C2–O4	119.76(10)
C24–C2–C3	117.48(9)
C23–C24–C2	118.50(10)
C25–C24–C2	119.97(10)
C25–C24–C23	121.53(10)
H23–C23–C24	118.1(10)
C22–C23–C24	124.03(11)
C22–C23–H23	117.8(10)
C21–C22–C23	117.78(10)
C29–C22–C23	119.04(10)
C29–C22–C21	123.17(11)
C22–C21–C3	121.03(11)
H21–C21–C3	117.9(10)
H21–C21–C22	121.0(10)
C26–C25–C24	109.00(10)
C28–C25–C24	109.92(10)
C28–C25–C26	112.50(11)
C27–C25–C24	112.11(10)
C27–C25–C26	106.27(11)
C27–C25–C28	107.03(11)
H26B–C26–C25	112.1(12)
H26C–C26–C25	112.4(10)
H26C–C26–H26B	108.2(15)
H26A–C26–C25	111.2(11)
H26A–C26–H26B	107.1(16)
H26A–C26–H26C	105.5(15)
H28C–C28–C25	108.3(12)
H28A–C28–C25	110.2(11)
H28A–C28–H28C	109.3(16)
H28B–C28–C25	113.2(10)
H28B–C28–H28C	108.4(16)

H28B–C28–H28A	107.5(15)
H27C–C27–C25	108.0(11)
H27B–C27–C25	108.9(12)
H27B–C27–H27C	107.7(16)
H27A–C27–C25	112.9(11)
H27A–C27–H27C	113.6(16)
H27A–C27–H27B	105.6(16)
C31–C29–C22	112.25(10)
C30–C29–C22	109.52(11)
C30–C29–C31	108.80(13)
C32–C29–C22	108.91(11)
C32–C29–C31	107.79(12)
C32–C29–C30	109.53(13)
H31B–C31–C29	111.2(12)
H31A–C31–C29	109.1(11)
H31A–C31–H31B	109.9(16)
H31C–C31–C29	110.1(10)
H31C–C31–H31B	107.5(16)
H31C–C31–H31A	109.1(15)
H30B–C30–C29	113.5(12)
H30A–C30–C29	106.9(12)
H30A–C30–H30B	108.8(17)
H30C–C30–C29	110.3(12)
H30C–C30–H30B	106.1(17)
H30C–C30–H30A	111.3(18)
H32A–C32–C29	115.9(12)
H32B–C32–C29	110.6(12)
H32B–C32–H32A	101.7(17)
H32C–C32–C29	111.1(11)
H32C–C32–H32A	109.5(16)
H32C–C32–H32B	107.4(16)
H33B–C33–O6	106.2(11)
H33A–C33–O6	107.0(10)
H33A–C33–H33B	111.1(16)
C34–C33–O6	105.15(11)
C34–C33–H33B	114.3(11)
C34–C33–H33A	112.5(10)
H34A–C34–C33	116.2(11)
H34B–C34–C33	110.8(11)
H34B–C34–H34A	106.1(15)
C35–C34–C33	101.95(11)
C35–C34–H34A	113.0(10)
C35–C34–H34B	108.6(11)
H35A–C35–C34	111.6(11)
H35B–C35–C34	112.8(12)
H35B–C35–H35A	103.0(16)
C36–C35–C34	102.64(11)
C36–C35–H35A	112.1(11)
C36–C35–H35B	115.0(13)
C35–C36–O6	106.18(11)
H36B–C36–O6	105.4(11)
H36B–C36–C35	113.1(11)
H36A–C36–O6	107.4(11)
H36A–C36–C35	109.4(11)
H36A–C36–H36B	114.8(16)
C38–Si1–O7$^{\#1}$	107.68(11)
C40–Si1–O7$^{\#1}$	110.24(11)
C40–Si1–C38	110.08(13)
C39–Si1–O7$^{\#1}$	107.98(10)
C39–Si1–C38	111.31(9)
C39–Si1–C40	109.51(13)
Si1–O7–Si1$^{\#1}$	147.96(16)
H38A–C38–Si1	105.7(15)
H38C–C38–Si1	111.8(15)
H38C–C38–H38A	105(2)
H38B–C38–Si1	108.5(14)
H38B–C38–H38A	114(2)
H38B–C38–H38C	111(2)
H40A–C40–Si1	115.3(14)
H40C–C40–Si1	106.6(13)
H40C–C40–H40A	115(2)
H40B–C40–Si1	110.8(15)
H40B–C40–H40A	108(2)
H40B–C40–H40C	101(2)
H39A–C39–Si1	113.5(15)
H39C–C39–Si1	112.3(16)
H39C–C39–H39A	108(2)
H39B–C39–Si1	110.2(15)
H39B–C39–H39A	106(2)
H39B–C39–H39C	106(2)

Symmetry transformations used to generate equivalent atoms:
#1: 1-X, +Y, 1.5-Z;

Table S5. Torsion angles for **II**

Atom–Atom–Atom–Atom	Torsion Angle [°]
Co1–O1–S1–O2	−10.55(10)
Co1–O1–S1–O3	−148.10(8)
Co1–O1–S1–C1	101.41(8)
Co1–O4–C2–C3	0.24(11)
Co1–O4–C2–C24	179.45(10)
Co1–O5–C6–C5	−12.70(11)
Co1–O5–C6–C7	167.86(10)
Co1–O6–C33–C34	−166.89(12)
Co1–O6–C36–C35	−168.68(11)
Co1–C4–N1–C20	−174.28(9)
Co1–C4–N1–C3	1.83(12)
Co1–C4–N2–C19	−174.75(9)
Co1–C4–N2–C5	−1.28(12)
O4–C2–C3–N1	−3.69(13)
O4–C2–C3–C21	178.40(11)
O4–C2–C24–C23	−178.70(11)

O4–C2–C24–C25	1.09(13)
O5–C6–C5–N2	−0.52(13)
O5–C6–C5–C10	179.81(11)
O5–C6–C7–C8	179.22(12)
O5–C6–C7–C15	−0.21(14)
O6–C33–C34–C35	−34.49(12)
O6–C36–C35–C34	−28.69(11)
N1–C4–N2–C19	4.39(11)
N1–C4–N2–C5	177.86(9)
N1–C20–C19–N2	15.24(9)
N1–C3–C2–C24	177.08(10)
N1–C3–C21–C22	−176.57(10)
N2–C5–C6–C7	178.93(11)
N2–C5–C10–C9	−178.53(11)
C5–C6–C7–C8	−0.24(13)
C5–C6–C7–C15	−179.67(12)
C5–C10–C9–C8	−0.51(14)
C5–C10–C9–C11	179.61(11)
C6–C7–C8–C9	0.90(15)
C6–C7–C15–C16	63.19(14)
C6–C7–C15–C18	−178.09(17)
C6–C7–C15–C17	−59.70(13)
C7–C8–C9–C10	−0.53(16)
C7–C8–C9–C11	179.36(13)
C8–C9–C11–C12	176.86(13)
C8–C9–C11–C13	56.81(14)
C8–C9–C11–C14	−63.19(14)
C3–C2–C24–C23	0.56(13)
C3–C2–C24–C25	−179.66(10)
C3–C21–C22–C23	−1.54(14)
C3–C21–C22–C29	176.94(11)
C2–C24–C23–C22	−0.83(14)
C2–C24–C25–C26	−60.73(12)
C2–C24–C25–C28	62.99(12)
C2–C24–C25–C27	−178.10(12)
C24–C23–C22–C21	1.32(16)
C24–C23–C22–C29	−177.23(13)
C23–C22–C29–C31	172.35(13)
C23–C22–C29–C30	−66.69(15)
C23–C22–C29–C32	53.07(14)
C33–C34–C35–C36	38.29(12)
Si1[#1]–O7–Si1–C38	146.12(8)
Si1[#1]–O7–Si1–C40	26.01(11)
Si1[#1]–O7–Si1–C39	−93.58(8)

Symmetry transformations used to generate equivalent atoms:
#1: 1-X, +Y, 1.5-Z;

DFT ORCA Input Files

List S1. ORCA (v 4.2.1) input files for the DFT geometry optimized form of **II** in the doublet state using crystal geometry as an input.

ORCA input files
Geometry optimization, doublet

```
! BP86 def2-TZVP def2/J Opt
%pal
nprocs 6
end

*xyz 0 2
Co       24.30376       5.66721       11.24965
S        22.33485       5.35286       13.91412
F        24.57073       4.59284       15.08751
F        23.81340       6.49432       15.75914
F        22.86284       4.65735       16.38283
F        26.17617       4.28668        9.75139
F        24.58871       5.08218        8.57968
F        26.12436       6.37018        9.34200
O        23.12451       6.13083       12.96088
O        22.13281       3.97693       13.54181
O        21.19353       6.06216       14.41822
O        22.84268       6.21502       10.25633
O        25.77584       5.35056       12.32416
O        24.79887       7.67974       11.38478
N        22.68913       3.41044       10.64066
N        24.55126       2.88274       11.68076
C        23.45715       5.26838       15.35943
C        21.83818       5.53216        9.78263
C        21.69544       4.12620        9.98407
C        23.82236       3.90208       11.19554
C        25.80499       2.95601       12.29326
C        26.37736       4.21844       12.60176
C        27.66189       4.22749       13.25034
C        28.25709       3.00960       13.54031
H        29.08792       3.08747       13.96373
C        27.69417       1.75953       13.22546
C        26.46692       1.75191       12.59542
H        26.06169       0.93960       12.37193
C        28.45131       0.47397       13.58073
C        28.74038       0.45012       15.09035
H        29.29010       1.20395       15.38182
H        27.91114       0.47698       15.60564
C        29.77229       0.43546       12.80621
C        28.33927       5.55098       13.62366
C        27.47960       6.29016       14.64822
H        26.54971       6.40625       14.37295
H        27.89069       7.15620       14.84231
```

H	27.37817	5.81145	15.49373
C	28.57971	6.40323	12.36224
H	29.22393	5.97955	11.76394
H	28.90991	7.26108	12.69096
H	27.75484	6.54704	11.85915
C	29.71973	5.31478	14.26438
H	29.59803	4.82588	15.10121
H	30.14238	6.15913	14.51660
H	30.38036	4.81726	13.73991
C	23.94073	1.57462	11.36791
H	23.89876	0.99420	12.15312
H	24.50997	1.22694	10.65486
C	22.55157	1.95880	10.89472
H	22.28326	1.48555	10.08362
H	21.87787	1.83898	11.59190
C	20.57741	3.43026	9.48598
H	20.52965	2.50848	9.63932
C	19.60122	4.07664	8.76875
C	19.74144	5.47110	8.57567
H	19.09428	5.93070	8.07925
C	20.80315	6.21085	9.03834
C	20.90301	7.71436	8.78679
C	20.91982	8.44722	10.13590
H	20.05364	8.33142	10.57469
H	21.11093	9.39889	10.01847
H	21.60418	8.10586	10.74506
C	19.68465	8.25183	8.01779
H	19.63098	7.85444	7.12718
H	19.80400	9.21068	7.87047
H	18.87265	8.11592	8.54026
C	22.14250	8.02052	7.93377
H	22.01363	7.69209	7.02195
H	22.96142	7.64324	8.31310
H	22.22829	8.99374	7.90554
C	18.40243	3.35886	8.14523
C	17.11527	3.83585	8.82438
H	17.17068	3.57451	9.76459
H	16.31393	3.42366	8.44839
H	17.01131	4.79858	8.69560
C	18.50288	1.84113	8.29071
H	17.73870	1.41659	7.85543
H	18.53484	1.59330	9.23510
H	19.32144	1.53440	7.85710
C	18.35149	3.68772	6.64179
H	19.19026	3.49405	6.18179
H	18.19966	4.63767	6.47409
H	17.61859	3.20528	6.21186
C	24.57663	8.73025	10.37224
H	23.62769	8.96070	10.37759

H	24.88057	8.35154	9.52407
C	25.40134	9.90360	10.83575
H	25.13246	10.77956	10.49451
H	26.33057	9.80117	10.55297
C	25.28652	9.79499	12.34521
H	24.41571	10.14167	12.61914
H	25.99347	10.25804	12.83460
C	25.34175	8.29565	12.57771
H	26.27287	8.02110	12.67593
H	24.81653	7.98805	13.34238
C	25.33904	5.33317	9.67439
C	27.99750	-0.40556	13.09592
H	27.86997	-0.21097	12.05152
H	27.04499	-0.60595	13.54030
H	28.63693	-1.25368	13.22523
H	30.39933	1.23963	13.13031
H	29.57506	0.53656	11.75941
H	30.25142	-0.29628	12.73873
H	29.19155	-0.17776	15.04814
*			

List S2. ORCA (v 4.2.1) input files for the DFT geometry optimized form of **II** in the quartet state using crystal geometry as an input.

Geometry optimization, quartet

```
! BP86 def2-TZVP def2/J Opt
%pal
nprocs 12
end
%scf
maxiter 125
ConvForced false
end


*xyz 0 4
  Co     24.580246      5.728418     11.036616
  S      22.228195      5.145754     13.381102
  F      23.750467      5.460987     15.572352
  F      22.287516      7.060328     15.260084
  F      21.630394      5.097393     15.976864
  F      26.782367      4.547158      9.652107
  F      25.105578      5.018574      8.341360
  F      26.374184      6.619154      9.116277
  O      23.238689      5.989829     12.652434
  O      22.562100      3.720728     13.417347
  O      20.850376      5.503485     13.083196
  O      23.109478      6.255352      9.819262
  O      26.007435      5.303371     12.319223
  O      25.082456      7.799520     11.351914
  N      22.933545      3.459406     10.420212
  N      24.839433      2.878780     11.398251
  C      22.492300      5.726051     15.151731
  C      22.026471      5.606054      9.534355
  C      21.885587      4.199216      9.847459
  C      24.121962      3.902981     10.890463
  C      26.075998      2.929909     12.066108
  C      26.617701      4.181279     12.540358
  C      27.847535      4.142470     13.302004
  C      28.455132      2.911818     13.507911
  H      29.393325      2.886847     14.060006
  C      27.930814      1.684104     13.043745
  C      26.732701      1.721145     12.334872
  H      26.301354      0.791971     11.971921
  C      28.680657      0.376079     13.333994
  C      28.817463      0.195168     14.864361
  H      29.379636      1.019328     15.323360
  H      27.829828      0.152186     15.345556
  C      30.090101      0.442184     12.698482
  C      28.465600      5.440633     13.851351
  C      27.480446      6.102040     14.845256
```

H	26.518752	6.328847	14.373965
H	27.912875	7.039064	15.229091
H	27.294928	5.439509	15.703014
C	28.791872	6.413347	12.692464
H	29.500994	5.956639	11.985679
H	29.258064	7.324911	13.096901
H	27.890779	6.696473	12.138437
C	29.777371	5.177381	14.614845
H	29.623644	4.520068	15.483771
H	30.166612	6.133869	14.992043
H	30.553890	4.735619	13.972720
C	24.101053	1.598980	11.291274
H	24.016822	1.144465	12.286071
H	24.636753	0.911476	10.621950
C	22.747217	2.019960	10.723898
H	22.483950	1.472667	9.810047
H	21.942752	1.918800	11.465205
C	20.694221	3.524729	9.544801
H	20.598944	2.474899	9.804945
C	19.616602	4.170770	8.946574
C	19.775960	5.533082	8.604770
H	18.941835	6.036121	8.119014
C	20.925063	6.263418	8.857970
C	21.032579	7.744096	8.455530
C	21.242096	8.617894	9.714576
H	20.383723	8.529741	10.396507
H	21.338747	9.674899	9.420895
H	22.139129	8.318925	10.266681
C	19.755381	8.252507	7.759002
H	19.540069	7.706920	6.828317
H	19.891946	9.311234	7.495531
H	18.873538	8.187409	8.414196
C	22.204287	7.930611	7.461077
H	22.026251	7.355407	6.540546
H	23.157131	7.598884	7.887550
H	22.292045	8.992395	7.184238
C	18.281945	3.466027	8.670601
C	17.164157	4.178465	9.470484
H	17.371437	4.145570	10.549813
H	16.197094	3.684952	9.288376
H	17.061488	5.233027	9.179725
C	18.301429	1.985391	9.087301
H	17.322329	1.529812	8.880534
H	18.498018	1.866253	10.163331
H	19.055655	1.412347	8.527637
C	17.959441	3.541078	7.159792
H	18.728299	3.026629	6.565146
H	17.897145	4.579257	6.806419
H	16.989554	3.062176	6.955327

C	25.052721	8.883172	10.370829
H	24.299910	8.614536	9.621288
H	26.035474	8.940342	9.884391
C	24.704184	10.165331	11.149126
H	23.934005	10.754885	10.634840
H	25.595012	10.800240	11.260811
C	24.236594	9.652302	12.523383
H	23.165466	9.406375	12.514150
H	24.420233	10.373562	13.331329
C	25.053979	8.381232	12.686537
H	26.090469	8.596596	12.998166
H	24.609956	7.638099	13.353826
C	25.764766	5.440875	9.464162
C	27.954384	-0.856336	12.768038
H	27.849670	-0.804669	11.673903
H	26.954338	-0.981912	13.209178
H	28.531853	-1.762431	13.001800
H	30.679691	1.275666	13.104370
H	30.023640	0.574562	11.608823
H	30.643333	-0.488410	12.899249
H	29.349931	-0.741857	15.089665

*

List S3. ORCA (v 4.2.1) input files for generating spin density and molecular orbital plots of **II** using DFT optimized coordinates as an input.

```
Spin density and molecular orbital plots doublet
! BP86 def2-TZVP def2/J NormalPrint Printbasis PrintMOs keepdens
%plots
dim1 160
dim2 160
dim3 160

Format CUBE

SpinDens("spindensity");

ElDens("electrondensity");

end
%pal
nprocs 6
end

%output
           print[p_mos] true
end

*xyz 0 2
Co        24.50795        5.63882        11.07358
S         22.30602        5.07689        13.63946
F         24.13689        4.71282        15.57011
F         23.00358        6.57838        15.75222
F         22.06087        4.66959        16.26470
F         26.49520        4.44938         9.51220
F         24.74356        5.03929         8.36196
F         26.15095        6.55516         9.06225
O         23.36688        5.83419        12.89262
O         22.34220        3.62929        13.41489
O         21.00598        5.72501        13.62334
O         23.03128        6.25754        10.13489
O         26.01377        5.32943        12.10634
O         25.05176        7.71929        11.40239
N         22.87187        3.43781        10.46665
N         24.72844        2.87579        11.53824
C         22.91321        5.27183        15.40983
C         21.99569        5.59581         9.67810
C         21.86004        4.17803         9.84991
C         24.02614        3.89695        10.99879
C         25.96975        2.93317        12.17676
C         26.59046        4.19958        12.43070
C         27.87899        4.20904        13.07982
C         28.43489        2.98740        13.43585
```

H	29.40633	2.99146	13.92678
C	27.82338	1.73441	13.20140
C	26.58790	1.73329	12.56663
H	26.09246	0.78846	12.36145
C	28.52973	0.44625	13.64852
C	28.70411	0.47198	15.18563
H	29.29877	1.33510	15.51468
H	27.72846	0.52328	15.68979
C	29.91952	0.36031	12.97544
C	28.60349	5.53023	13.39487
C	27.77700	6.31884	14.43817
H	26.76347	6.51528	14.07620
H	28.26436	7.28170	14.65643
H	27.70223	5.75479	15.37900
C	28.79593	6.37202	12.11083
H	29.41349	5.82951	11.37962
H	29.31681	7.30918	12.36126
H	27.84130	6.62238	11.63739
C	30.00383	5.29511	13.99418
H	29.96525	4.76763	14.95879
H	30.47957	6.26949	14.17422
H	30.65647	4.73006	13.31151
C	23.99591	1.60032	11.42602
H	23.84052	1.18071	12.42789
H	24.57290	0.88965	10.81783
C	22.68099	2.00383	10.75930
H	22.49495	1.44849	9.83095
H	21.82509	1.89577	11.43880
C	20.70873	3.50224	9.41012
H	20.62596	2.43207	9.57731
C	19.67087	4.17612	8.78189
C	19.82504	5.56727	8.58953
H	19.01908	6.10238	8.09171
C	20.93158	6.29723	8.99905
C	21.01591	7.81042	8.73097
C	21.15284	8.58354	10.06406
H	20.27377	8.40855	10.70079
H	21.21675	9.66429	9.86092
H	22.03760	8.27494	10.62953
C	19.75296	8.35053	8.03101
H	19.58386	7.88380	7.04944
H	19.87427	9.43053	7.86323
H	18.85002	8.21190	8.64496
C	22.21182	8.09253	7.79011
H	22.03751	7.64029	6.80246
H	23.14883	7.68368	8.18254
H	22.33524	9.17749	7.64800
C	18.39211	3.47428	8.30519
C	17.16999	4.09682	9.02169

H	17.25248	3.98136	10.11233
H	16.24488	3.60097	8.69033
H	17.06981	5.16913	8.80251
C	18.40622	1.96527	8.60319
H	17.47279	1.51070	8.24156
H	18.47549	1.76190	9.68238
H	19.23830	1.45425	8.09664
C	18.24113	3.66443	6.77749
H	19.08824	3.21489	6.23889
H	18.19071	4.72685	6.50076
H	17.31681	3.18260	6.42364
C	24.93168	8.81054	10.43832
H	24.16504	8.51584	9.71583
H	25.89306	8.91834	9.91660
C	24.55088	10.06867	11.24118
H	23.73706	10.62688	10.76038
H	25.41452	10.74394	11.33068
C	24.15749	9.51707	12.62250
H	23.10324	9.20620	12.64219
H	24.32347	10.23862	13.43384
C	25.05265	8.29479	12.73984
H	26.08860	8.57435	12.99906
H	24.68631	7.52833	13.42647
C	25.51861	5.40301	9.42955
C	27.73313	-0.81686	13.27812
H	27.59203	-0.90846	12.19056
H	26.74614	-0.83279	13.76228
H	28.27716	-1.71048	13.61645
H	30.55248	1.22061	13.23342
H	29.82353	0.32765	11.88027
H	30.44435	-0.55068	13.30231
H	29.21894	-0.43999	15.52508
*			

TDDFT

List S4. ORCA (v 4.2.1) input files for the TDDFT excited state calculation of **II** using DFT optimized geometry as the input.

```
TD-DFT doublet
!BP86 def2-TZVP def2/J TightSCF
%maxcore 10000
%TDDFT
maxdim 300
nroots 50
end
%pal
nprocs 12
end

*xyz 0 2
  Co    24.507950    5.638820   11.073580
  S     22.306020    5.076890   13.639460
  F     24.136890    4.712820   15.570110
  F     23.003580    6.578380   15.752220
  F     22.060870    4.669590   16.264700
  F     26.495200    4.449380    9.512200
  F     24.743560    5.039290    8.361960
  F     26.150950    6.555160    9.062250
  O     23.366880    5.834190   12.892620
  O     22.342200    3.629290   13.414890
  O     21.005980    5.725010   13.623340
  O     23.031280    6.257540   10.134890
  O     26.013770    5.329430   12.106340
  O     25.051760    7.719290   11.402390
  N     22.871870    3.437810   10.466650
  N     24.728440    2.875790   11.538240
  C     22.913210    5.271830   15.409830
  C     21.995690    5.595810    9.678100
  C     21.860040    4.178030    9.849910
  C     24.026140    3.896950   10.998790
  C     25.969750    2.933170   12.176760
  C     26.590460    4.199580   12.430700
  C     27.878990    4.209040   13.079820
  C     28.434890    2.987400   13.435850
  H     29.406330    2.991460   13.926780
  C     27.823380    1.734410   13.201400
  C     26.587900    1.733290   12.566630
  H     26.092460    0.788460   12.361450
  C     28.529730    0.446250   13.648520
  C     28.704110    0.471980   15.185630
  H     29.298770    1.335100   15.514680
  H     27.728460    0.523280   15.689790
  C     29.919520    0.360310   12.975440
```

C	28.603490	5.530230	13.394870
C	27.777000	6.318840	14.438170
H	26.763470	6.515280	14.076200
H	28.264360	7.281700	14.656430
H	27.702230	5.754790	15.379000
C	28.795930	6.372020	12.110830
H	29.413490	5.829510	11.379620
H	29.316810	7.309180	12.361260
H	27.841300	6.622380	11.637390
C	30.003830	5.295110	13.994180
H	29.965250	4.767630	14.958790
H	30.479570	6.269490	14.174220
H	30.656470	4.730060	13.311510
C	23.995910	1.600320	11.426020
H	23.840520	1.180710	12.427890
H	24.572900	0.889650	10.817830
C	22.680990	2.003830	10.759300
H	22.494950	1.448490	9.830950
H	21.825090	1.895770	11.438800
C	20.708730	3.502240	9.410120
H	20.625960	2.432070	9.577310
C	19.670870	4.176120	8.781890
C	19.825040	5.567270	8.589530
H	19.019080	6.102380	8.091710
C	20.931580	6.297230	8.999050
C	21.015910	7.810420	8.730970
C	21.152840	8.583540	10.064060
H	20.273770	8.408550	10.700790
H	21.216750	9.664290	9.860920
H	22.037600	8.274940	10.629530
C	19.752960	8.350530	8.031010
H	19.583860	7.883800	7.049440
H	19.874270	9.430530	7.863230
H	18.850020	8.211900	8.644960
C	22.211820	8.092530	7.790110
H	22.037510	7.640290	6.802460
H	23.148830	7.683680	8.182540
H	22.335240	9.177490	7.648000
C	18.392110	3.474280	8.305190
C	17.169990	4.096820	9.021690
H	17.252480	3.981360	10.112330
H	16.244880	3.600970	8.690330
H	17.069810	5.169130	8.802510
C	18.406220	1.965270	8.603190
H	17.472790	1.510700	8.241560
H	18.475490	1.761900	9.682380
H	19.238300	1.454250	8.096640
C	18.241130	3.664430	6.777490
H	19.088240	3.214890	6.238890

H	18.190710	4.726850	6.500760
H	17.316810	3.182600	6.423640
C	24.931680	8.810540	10.438320
H	24.165040	8.515840	9.715830
H	25.893060	8.918340	9.916600
C	24.550880	10.068670	11.241180
H	23.737060	10.626880	10.760380
H	25.414520	10.743940	11.330680
C	24.157490	9.517070	12.622500
H	23.103240	9.206200	12.642190
H	24.323470	10.238620	13.433840
C	25.052650	8.294790	12.739840
H	26.088600	8.574350	12.999060
H	24.686310	7.528330	13.426470
C	25.518610	5.403010	9.429550
C	27.733130	-0.816860	13.278120
H	27.592030	-0.908460	12.190560
H	26.746140	-0.832790	13.762280
H	28.277160	-1.710480	13.616450
H	30.552480	1.220610	13.233420
H	29.823530	0.327650	11.880270
H	30.444350	-0.550680	13.302310
H	29.218940	-0.439990	15.525080

*

Figure S31. DFT Molecular Orbitals of **II** from HOMO-18 to LUMO+2. Plotted in IboView (isovalue = 0.2)

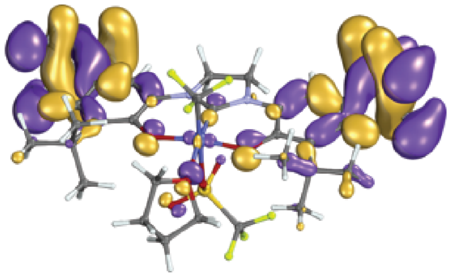

HOMO-18

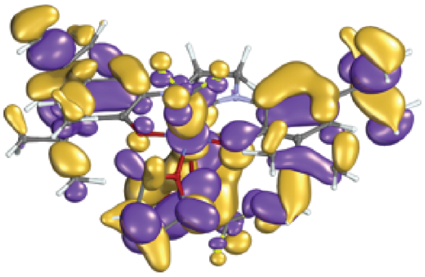

HOMO-17

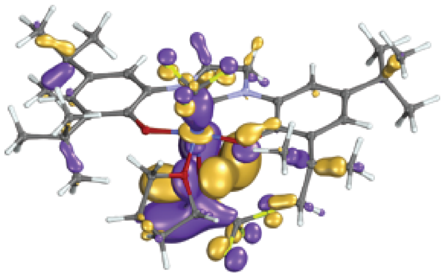

HOMO-16

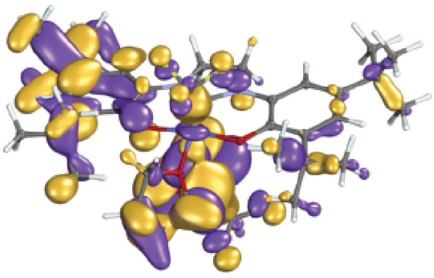

HOMO-15

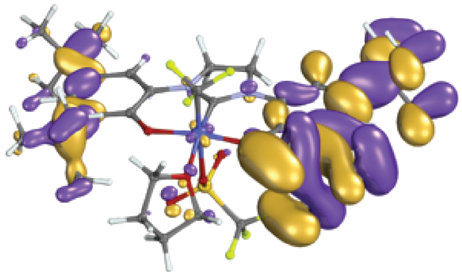

HOMO-14

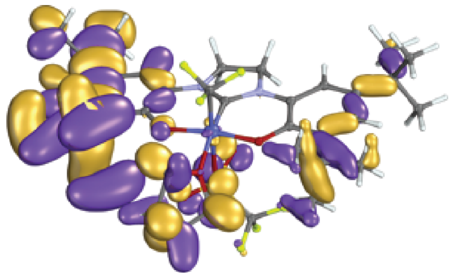

HOMO-13

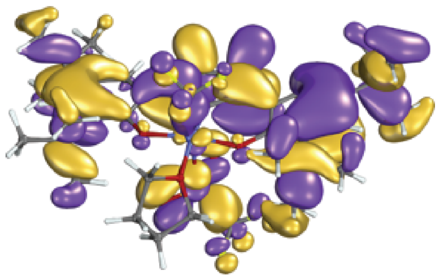

HOMO-12

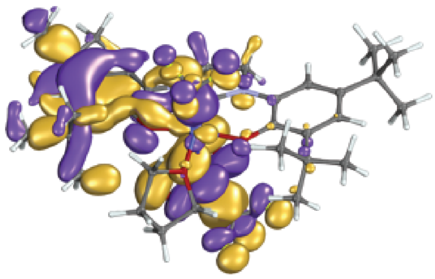

HOMO-11

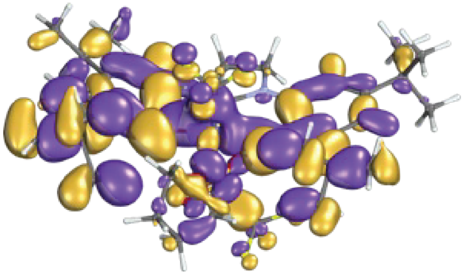

HOMO-10

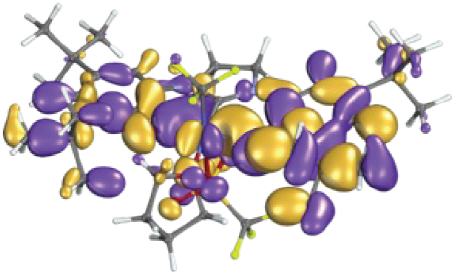

HOMO-9

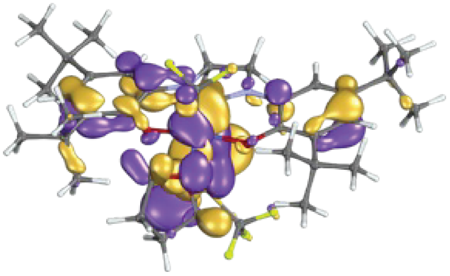

HOMO-8

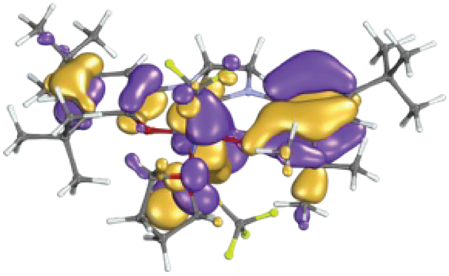

HOMO-7

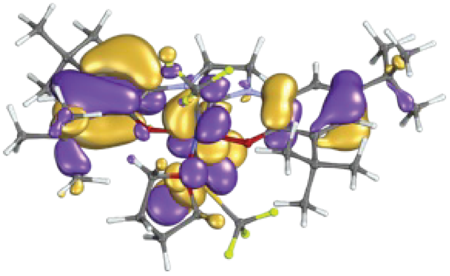

HOMO-6

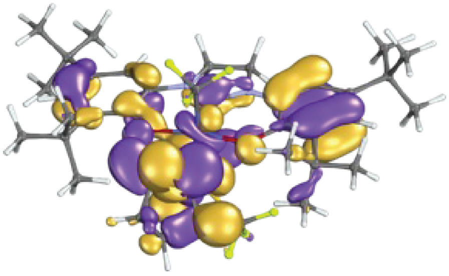

HOMO-5

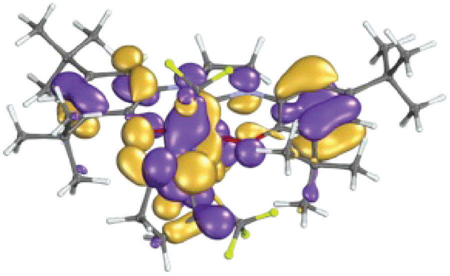

HOMO-4

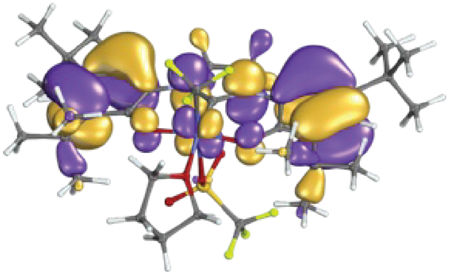

HOMO-3

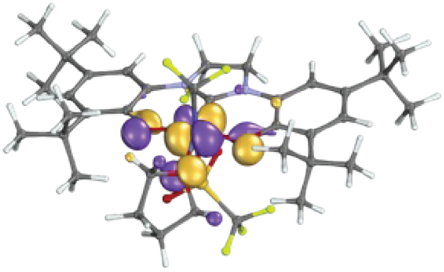

HOMO-2

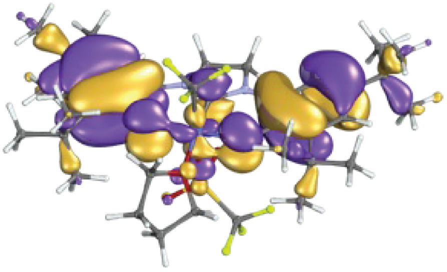

HOMO-1

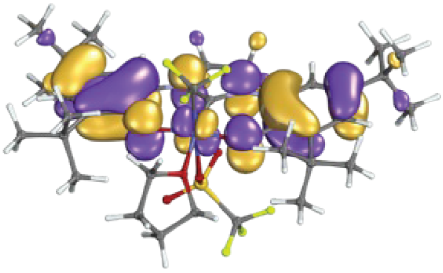

HOMO

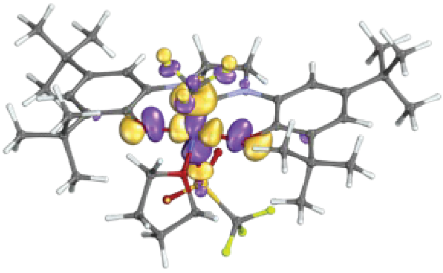

LUMO

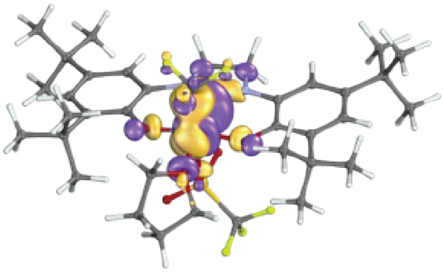

LUMO+1

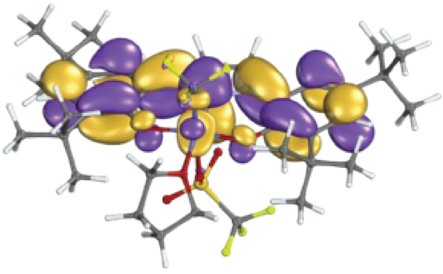

LUMO+2

List S5. TDDFT Transitions of **II**. Up to three largest orbital contributions are listed, minimum of 10%. Contributions are expressed as a sum of α and β orbitals.

STATE 1: 1.204 eV 1030 nm H-2→HOMO (83%), H-1→HOMO (12%)
STATE 2: 1.308 eV 948 nm H-1→HOMO (54%), H-2→HOMO (13%), HOMO→LUMO (12%)
STATE 3: 1.355 eV 915 nm H-4→HOMO (46%), H-3→HOMO (38%)
STATE 4: 1.481 eV 837 nm H-4→HOMO (45%), H-3→HOMO (43%)
STATE 5: 1.576 eV 787 nm HOMO→LUMO (79%), H-1→HOMO (10%)
STATE 6: 1.657 eV 748 nm H-5→HOMO (91%)
STATE 7: 1.845 eV 672 nm HOMO→L+1 (93%)
STATE 8: 1.969 eV 630 nm H-6→HOMO (66%)
STATE 9: 1.992 eV 622 nm H-7→HOMO (85%)
STATE 10: 2.047 eV 606 nm H-2→LUMO (56%), H-1→LUMO (19%), H-3→ LUMO(14%)
STATE 11: 2.151 eV 576 nm H-1→LUMO (76%), H-2→LUMO (18%)
STATE 12: 2.246 eV 552 nm H-8→HOMO (92%)
STATE 13: 2.350 eV 527 nm H-9→HOMO (96%)
STATE 14: 2.384 eV 520 nm H-1→L+2 (36%), H-2→L+1 (16%), H-3→LUMO (13%)
STATE 15: 2.394 eV 518 nm H-1→LUMO (42%), H-3→LUMO (30%)
STATE 16: 2.408 eV 515 nm H-1→L+1 (25%), H-3→LUMO (23%), H-1→LUMO (20%)
STATE 17: 2.453 eV 505 nm H-2→L+1 (50%), H-1→L+1 (32%)
STATE 18: 2.496 eV 497 nm H-4→LUMO (42%), H-5→LUMO (24%)
STATE 19: 2.556 eV 485 nm H-10→HOMO (86%), H-1→L+1 (10%)
STATE 20: 2.604 eV 476 nm H-1→L+1 (70%)
STATE 21: 2.617 eV 474 nm HOMO→L+2 (73%)
STATE 22: 2.669 eV 464 nm H-3→L+1 (38%), H-11→HOMO (28%)
STATE 23: 2.682 eV 462 nm H-11→HOMO (56%), H-3→L+1 (17%)
STATE 24: 2.722 eV 455 nm H-4→LUMO (37%), H-3→LUMO (37%)
STATE 25: 2.772 eV 447 nm H-4→LUMO (49%), H-5→LUMO (17%), H-3→LUMO (15%)
STATE 26: 2.783 eV 456 nm H-12→HOMO (33%), H-6→L+1 (24%), H-5→L+1 (15%)
STATE 27: 2.809 eV 441 nm H-12→HOMO (56%), H-6→L+1 (16%), H-5→L+1 (12%)
STATE 28: 2.841 eV 436 nm H-5→LUMO (45%), H-4→LUMO (39%)
STATE 29: 2.871 eV 432 nm H-13→HOMO (86%)
STATE 30: 2.890 eV 429 nm H-2→LUMO (47%), H-3→LUMO (12%), H-2→L+1 (12%)
STATE 31: 2.997 eV 414 nm H-1→L+2 (89%)
STATE 32: 3.002 eV 413 nm H-6→LUMO (47%), H-7→LUMO (14%)
STATE 33: 3.008 eV 412 nm H-3→L+1 (43%), H-2→L+1 (28%)
STATE 34: 3.038 eV 408 nm H-4→L+1 (74%), H-3→L+1 (11%)
STATE 35: 3.093 eV 401 nm H-5→L+1 (44%), H-4→L+1 (25%)
STATE 36: 3.112 eV 398 nm H-15→HOMO (64%), H-14→HOMO (11%)
STATE 37: 3.133 eV 386 nm H-5→LUMO (31%), H-4→L+1 (16%), H-3→L+1 (11%)
STATE 38: 3.138 eV 395 nm H-15→ HOMO (77%), H-14→HOMO (12%)
STATE 39: 3.220 eV 385 nm H-16→HOMO (94%)
STATE 40: 3.232 eV 384 nm H-5→LUMO (24%), H-6→LUMO (21%), H-17→HOMO (15%)
STATE 41: 3.237 eV 383 nm H-17→HOMO (68%), H-6→LUMO (22%)
STATE 42: 3.238 eV 382 nm H-6→LUMO (30%), H-7→LUMO (20%), H-17→HOMO (15%)
STATE 43: 3.292 eV 377 nm H-6→L+1 (30%), H-7→LUMO (22%), H-5→L+1 (18%)
STATE 44: 3.328 eV 372 nm H-7→LUMO (64%), H-6→LUMO (15%), H-7→L+1 (12%)
STATE 45: 3.350 eV 370 nm H-7→LUMO (31%), H-5→L+1 (29%), H-6→L+1 (18%)
STATE 46: 3.355 eV 369 nm H-7→LUMO (31%), H-5→LUMO (30%), H-5→L+1 (14%)
STATE 47: 3.387 eV 366 nm H-1→L+2 (76%)
STATE 48: 3.426 eV 362 nm H-8→LUMO (66%)
STATE 49: 3.436 eV 361 nm H-4→L+2 (44%), H-3→L+2 (37%)

STATE 50: 3.445 eV 360 nm H-18→HOMO (74%)

References

1. Wang, B.; Xiong, D.-C.; Ye, X.-S. Direct C–H trifluoromethylation of glycals by photoredox catalysis. *Org. Lett.* **2015**, *17* (22), 5698-5701.
2. Harris, C. F.; Bayless, M. B.; van Leest, N. P.; Bruch, Q. J.; Livesay, B. N.; Bacsa, J.; Hardcastle, K. I.; Shores, M. P.; de Bruin, B.; Soper, J. D. Redox-Active Bis(phenolate) N-Heterocyclic Carbene [OCO] Pincer Ligands Support Cobalt Electron Transfer Series Spanning Four Oxidation States. *Inorg. Chem.* **2017**, *56* (20), 12421-12435.
3. APEX5 suite for crystallographic software; Bruker axs, Madison, WI (2014).
4. Krause, L.; Herbst-Irmer, R.; Sheldrick, G. M.; Stalke, D. Comparison of silver and molybdenum microfocus X-ray sources for single-crystal structure determination. *J. Appl. Cryst.* **2015**, *48*, 3-10. DOI: 10.1107/S1600576714022985.
5. Sheldrick, G. M. SHELXT–Integrated space-group and crystal-structure determination. *Acta Crystallogr., Sect. A* **2015**, *71* (1), 3-8.
6. Sheldrick, G. M. Crystal structure refinement with SHELXL. *Acta Crystallogr., Sect. C* **2015**, *71* (1), 3-8.
7. Kleemiss, F.; Dolomanov, O. V.; Bodensteiner, M.; Peyerimhoff, N.; Midgley, L.; Bourhis, L. J.; Genoni, A.; Malaspina, L. A.; Jayatilaka, D.; Spencer, J. L. Accurate crystal structures and chemical properties from NoSpherA2. *Chem. Sci.* **2021**, *12* (5), 1675-1692.
8. Kratzert, D. *FinalCif*. https://dkratzert.de/finalcif.html (accessed 2023).
